# Neutrophils insert elastase into hepatocytes to regulate calcium signaling in alcohol-associated hepatitis

**DOI:** 10.1172/JCI171691

**Published:** 2024-06-25

**Authors:** Noriyoshi Ogino, M. Fatima Leite, Mateus T. Guerra, Emma Kruglov, Hiromitsu Asashima, David A. Hafler, Takeshi Ito, João P. Pereira, Brandon J. Peiffer, Zhaoli Sun, Barbara E. Ehrlich, Michael H. Nathanson

**Affiliations:** 1 Yale Liver Center, Yale University School of Medicine, New Haven, Connecticut, USA.; 2 INCT - NanoBiofar – Department of Physiology and Biophysics, Federal University of Minas Gerais, Belo Horizonte, Brazil.; 3 Department of Neurology,; 4 Department of Immunobiology, and; 5 Department of Immunobiology and Yale Stem Cell Center, Yale University School of Medicine, New Haven, Connecticut, USA.; 6 Department of Surgery, Johns Hopkins University School of Medicine, Baltimore, Maryland, USA.; 7 Department of Pharmacology, Yale University School of Medicine, New Haven, Connecticut, USA.; 8 Department of Pathology, New York University School of Medicine, New York, New York, USA.

**Keywords:** Hepatology, Calcium signaling, Hepatitis, Neutrophils

## Abstract

Neutrophil infiltration occurs in a variety of liver diseases, but it is unclear how neutrophils and hepatocytes interact. Neutrophils generally use granule proteases to digest phagocytosed bacteria and foreign substances or neutralize them in neutrophil extracellular traps. In certain pathological states, granule proteases play a destructive role against the host as well. More recently, nondestructive actions of neutrophil granule proteins have been reported, such as modulation of tissue remodeling and metabolism. Here, we report a completely different mechanism by which neutrophils act nondestructively, by inserting granules directly into hepatocytes. Specifically, elastase-containing granules were transferred to hepatocytes where elastase selectively degraded intracellular calcium channels to reduce cell proliferation without cytotoxicity. In response, hepatocytes increased expression of Serpin E2 and A3, which inhibited elastase activity. Elastase insertion was seen in patient specimens of alcohol-associated hepatitis, and the relationship between elastase-mediated ITPR2 degradation and reduced cell proliferation was confirmed in mouse models. Moreover, neutrophils from patients with alcohol-associated hepatitis were more prone to degranulation and more potent in reducing calcium channel expression than neutrophils from healthy individuals. This nondestructive and reversible action on hepatocytes defines a previously unrecognized role for neutrophils in the transient regulation of epithelial calcium signaling mechanisms.

## Abstract 2

Neutrophils transfer elastase-releasing granules into hepatocytes to transiently impair calcium signaling in alcohol-associated hepatitis.

## Graphical abstract

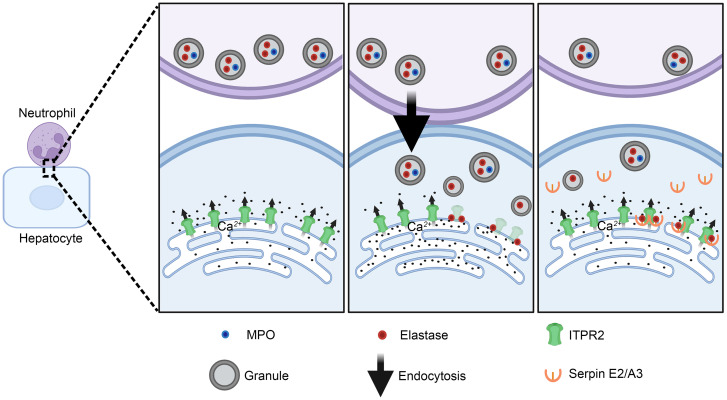

## Introduction

Neutrophils are the most abundant type of polymorphonucleated leukocytes in circulation, typically accounting for around 60% of the total in humans. When organs such as the liver, lungs, and pancreas are injured, inflammatory signals promote neutrophil infiltration into the organ’s parenchyma (1). Infiltrating neutrophils have been reported to directly damage host organs while using granule proteases in phagocytic digestion of bacteria and neutrophil extracellular trapping to control infection (2–4). However, accumulating evidence has emerged for a nondestructive role of neutrophils in host organs, including reports that granule proteases partially contribute to organ tissue remodeling and metabolic regulation (5–8).

Neutrophil infiltration is a hallmark of a range of liver diseases, including alcohol-associated hepatitis (AAH) (9). This is a potentially life-threatening condition, with a short-term mortality rate of 20%–50% and increasing incidence in the United States and worldwide, especially among young adults (25–34 years old) (10). As is the case for other diseases, it is commonly thought that in AAH, neutrophils damage host hepatocytes by nonspecific “killing” responses such as generation of reactive oxygen species and secretion of degradative enzymes contained in their granules (4, 11). However, histopathologic examination of liver biopsies from patients with AAH has shown that an increased number of neutrophils infiltrating the liver is correlated with improved, rather than worse, prognosis (12). Corticosteroids, which potently suppress the immune system and the inflammatory response, including neutrophil activation, are the only pharmacotherapy of proven efficacy in AAH, albeit with limited effectiveness (13, 14). This highlights the need for a better understanding of the interaction between infiltrating neutrophils and hepatocytes in AAH, to better guide therapy.

Intracellular calcium (Ca^2+^) signaling in the liver controls a variety of cellular functions (15, 16). In hepatocytes, Ca^2+^ release from intracellular stores is mediated by inositol 1,4,5-trisphosphate receptors (ITPRs) localized on the membrane of the endoplasmic reticulum (ER), and the type 2 isoform (ITPR2) accounts for 80% of the total pool (17). Genetic ablation or disease-mediated reductions in ITPR2 are associated with reduced bile secretion and impaired hepatocyte proliferation, respectively, which are 2 key liver functions compromised in patients with AAH (18, 19). Therefore, we investigated whether and how neutrophils regulate Ca^2+^ signaling in hepatocytes and the implications of this cell-to-cell communication for the pathogenesis of AAH.

## Results

### Neutrophils decrease Ca^2+^-associated proteins and cellular proliferation in hepatocytes without cellular damage.

**Figure 1:**
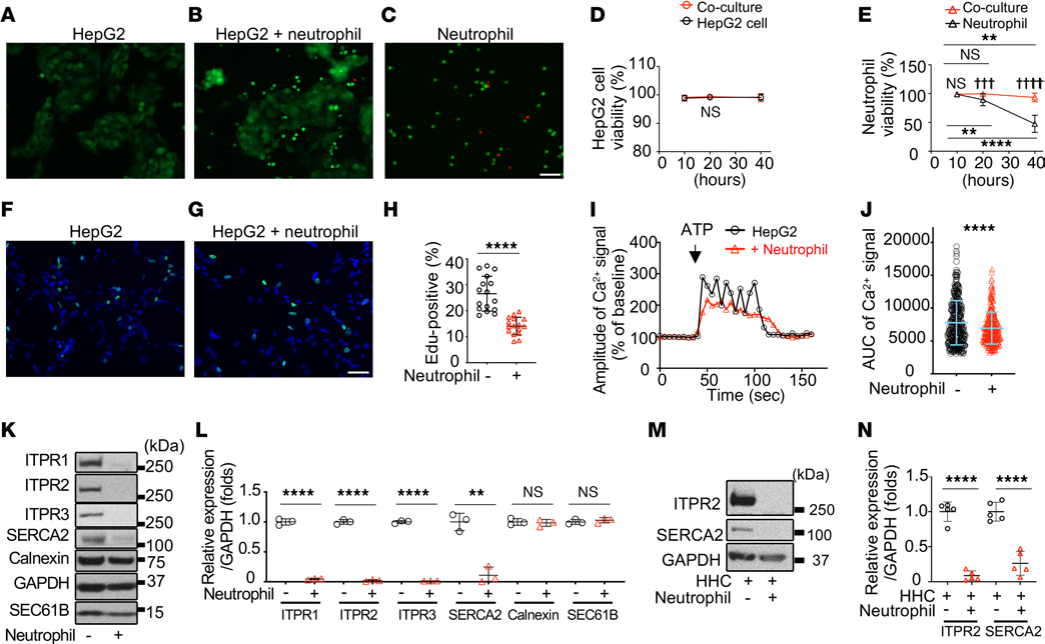
Neutrophils decrease Ca^2+^-related proteins in hepatocytes without causing cell death. (**A**–**C**) Representative images of HepG2 cells and neutrophils after 20 hours of culture, using double staining (calcein-AM for alive [green], ethidium homodimer-1 for dead [red]). HepG2 cells only (**A**), coculture (**B**), neutrophils only (**C**). (**D** and **E**) Graphs comparing time series of cell viability with and without coculture. (**D**) Viability of HepG2 cells does not decrease over time regardless of coculture. (**E**) Viability of neutrophils decreases over time unless they are cocultured. Differences between alone and coculture are represented by daggers, and changes over time are indicated by asterisks; 5 fields per coverslips were measured. (**F** and **G**) Representative images double labeled with EdU (green) and Hoechst 33342 (blue) of HepG2 cells alone (**F**) and cocultured with neutrophils (**G**) after 12 hours. (**H**) HepG2 cell proliferation (EdU-positive cells) is decreased by coculture with neutrophils; 5–6 fields were measured per coverslip. (**I**) Ca^2+^ signals in HepG2 cells are altered by neutrophils. Representative tracings of Fluo-4 fluorescence intensity of HepG2 cells alone or cocultured with neutrophils for 20 hours. Cells were stimulated with 20 μM ATP. (**J**) Ca^2+^ signaling in HepG2 cells as measured by the AUC upon ATP stimulation is diminished by neutrophils. Nine coverslips of HepG2 cells (362 total cells) and 8 coverslips of HepG2 cells cocultured with neutrophils (279 total cells) were analyzed. (**K**) Representative immunoblots and (**L**) quantitation of the blots show that ITPR1, ITPR2, ITPR3, and SERCA2 are decreased in HepG2 cells, but calnexin and SEC61B are not, after coculture with neutrophils for 1 hour. ITPR1, ITPR2, and ITPR3 were blotted onto different membranes because of their close molecular weights, and their ratios to GAPDH were measured separately. (**M**) Representative immunoblots and (**N**) quantitation of the blots shows that ITPR2 and SERCA2 are decreased in primary human hepatocytes (HHC) that are cocultured for 1 hour. The graph represents neutrophils from 5 healthy volunteers. In **A**–**N**, all are from 3 independent experiments. Data are mean ± SD. NS, not significant. **P < 0.01; ****P < 0.0001; ^†††^*P* < 0.001; ^††††^*P* < 0.0001 by unpaired, 2-tailed Student’s *t* test. Scale bars: 50 μm.

To mimic the interaction between infiltrating neutrophils and hepatocytes, liver-derived HepG2 cells were cocultured with human neutrophils (Figure 1, A–E). *Cxcl1* mRNA was elevated in the HepG2 cells, similar to the response of hepatocytes to infiltrating neutrophils in vivo (Supplemental Figure 1A; supplemental material available online with this article; https://doi.org/10.1172/JCI171691DS1) (20, 21). Although neutrophils can damage hepatocytes (3, 4), HepG2 cell viability was similar in cocultured cells and controls not exposed to neutrophils (Figure 1, A, B, and D). In contrast, neutrophil viability dropped progressively unless neutrophils were cocultured with HepG2 cells (Figure 1, B, C, and E). These results are consistent with recent reports showing that neutrophils in circulation have a short half-life, but infiltrating neutrophils change their phenotype to live from several days to 2 weeks (22–24). Neutrophils did not damage HepG2 cells by other measures as well, including albumin levels and mitochondrial membrane potential in the cells and alanine transaminase (ALT) levels in the culture medium (Supplemental Figure 1, B–E). Similar results were observed in primary mouse hepatocytes cocultured with mouse neutrophils (Supplemental Figure 1, F–K). Viability of mouse neutrophils also was significantly improved when cocultured with the hepatocytes (Supplemental Figure 1G).

Since hepatocyte proliferation is reduced in AAH (25–27), the potential role of neutrophils was examined. The number of 5-ethynyl-2′-deoxyuridine–positive (EdU-positive) HepG2 cells was significantly reduced when cocultured with neutrophils (Figure 1, F–H). Hepatocyte proliferation is regulated by Ca^2+^ signaling (28, 29), and ATP-induced Ca^2+^ signals in HepG2 cells were found to be significantly reduced by coculture with neutrophils (Figure 1, I and J). Moreover, examining expression of major proteins involved in intracellular Ca^2+^ signaling revealed that all 3 ITPR isoforms (ITPR1, ITPR2, and ITPR3) and the sarcoplasmic reticulum/ER Ca^2+^-ATPase 2 (SERCA2) pump were dramatically reduced (Figure 1, K and L). Conversely, the ER-resident proteins SEC61b and calnexin were unchanged. ITPR2, the most abundant ITPR isoform in hepatocytes (17), also was reduced in primary human and mouse hepatocytes cocultured with neutrophils for 1 hour (Figure 1, M and N, and Supplemental Figure 1, H and I) and SERCA2 was reduced in human hepatocytes (Figure 1M). Together, these results show that neutrophils interact with hepatocytes in a nondestructive manner to impair expression of Ca^2+^-handling proteins and Ca^2+^ signaling.

### Neutrophils decrease ITPR2 in HepG2 cells via a reversible nontranscriptional mechanism.

**Figure 2:**
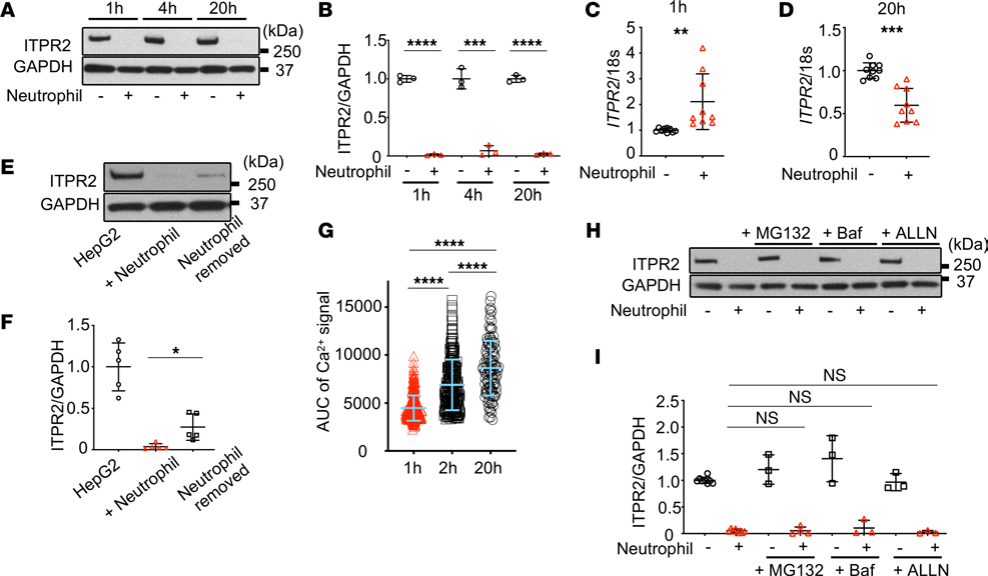
The decrease in ITPR2 in HepG2 cells induced by neutrophils is rapid and reversible. (**A**) Representative immunoblots and (**B**) quantitation of ITPR2 levels in HepG2 cells cocultured with neutrophils show that loss of ITPR2 persists for up to 20 hours in the continued presence of neutrophils. (**C** and **D**) RT-qPCR shows that *ITPR2* mRNA levels in HepG2 cells are increased after 1 hour and decreased after 20 hours of coculture with neutrophils. (**E**) Representative immunoblots and (**F**) quantitation show that ITPR2 levels begin to recover after neutrophils are removed. ITPR2 levels were measured in HepG2 cells cocultured with neutrophils for 20 hours, and then cells were washed to remove neutrophils and cultured for 2 more hours and collected (after neutrophil removal). Comparisons were relative to HepG2 cells cultured alone for 22 hours or cocultured with neutrophils. (**G**) Ca^2+^ signals in HepG2 cells progressively recover after neutrophils are removed. AUC of Fluo-4 fluorescence after stimulation with ATP (20 μM) was measured at 3 time points: after coculture with neutrophils for 1 hour (1 h, followed by complete removal of neutrophils for 1 hour [2 h] or 19 hours [20 h]). Data represent 5–7 coverslips each, with cell numbers *n* = 286, 208, 112. (**H**) Representative immunoblots and (**I**) quantitation shows that loss of ITPR2 in HepG2 cells is not blocked by treatment with MG132 (proteasome inhibitor, 50 μM), bafilomycin A1 (Baf, autophagy inhibitor, 50 nM), or Ac-Leu-Leu-Nle-aldehyde (ALLN, calpain inhibitor, 50 μM) for 1 hour, followed by coculture with neutrophils for 1 hour. Data are expressed as mean ± SD, *n* = 3–8. NS, not significant. *P < 0.05; **P < 0.01; ***P < 0.001; ****P < 0.0001 by unpaired, 2-tailed Student’s *t* test (**B**–**D** and **F**) or 1-way ANOVA with Tukey’s multiple-comparison test (**G** and **I**).

To understand the mechanism of the neutrophil-induced decrease in Ca^2+^ signaling proteins in hepatocytes, time-course experiments were performed focusing on ITPR2, because it is also the principal Ca^2+^ channel protein responsible for hepatocyte proliferation (28, 30, 31). ITPR2 protein levels in HepG2 cells decreased as early as 1 hour after coculture and this decrease persisted at 4 hours and 20 hours of coculture (Figure 2, A and B). Ca^2+^ signaling in HepG2 cells was similarly decreased 1 hour after coculture with neutrophils (Supplemental Figure 2, A and B). To test whether this reduction in ITPR2 is transcriptionally regulated, *ITPR2* gene expression was measured, but *ITPR2* mRNA was increased rather than decreased after 1 hour of coculture (Figure 2, C and D). To determine whether this reduction is reversible, cocultured neutrophils were removed, and then HepG2 cells recovered by monoculture. Under these conditions, ITPR2 levels in HepG2 cells rapidly recovered, as did Ca^2+^ signals (Figure 2, E–G). These data suggest neutrophil-induced loss of ITPR2 in HepG2 cells occurs through protein degradation rather than mRNA transcription. Indeed, longer exposure of ITPR2 immunoblots of HepG2 cell lysates after neutrophil coculture showed a ladder pattern (Supplemental Figure 2C). Inhibitors of autophagy, proteasomes, and calpain were tested (32, 33), but none were able to prevent the decrease in ITPR2 (Figure 2, H and I). Inhibitors of trypsin and caspase 3 also failed to prevent ITPR2 reduction (Supplemental Figure 2, D and E) (34, 35). Collectively, these data suggest neutrophils reduce ITPR2 by a transient proteolytic mechanism.

### Direct contact with intact neutrophils is necessary for the decrease in ITPR2 in hepatocytes.

**Figure 3:**
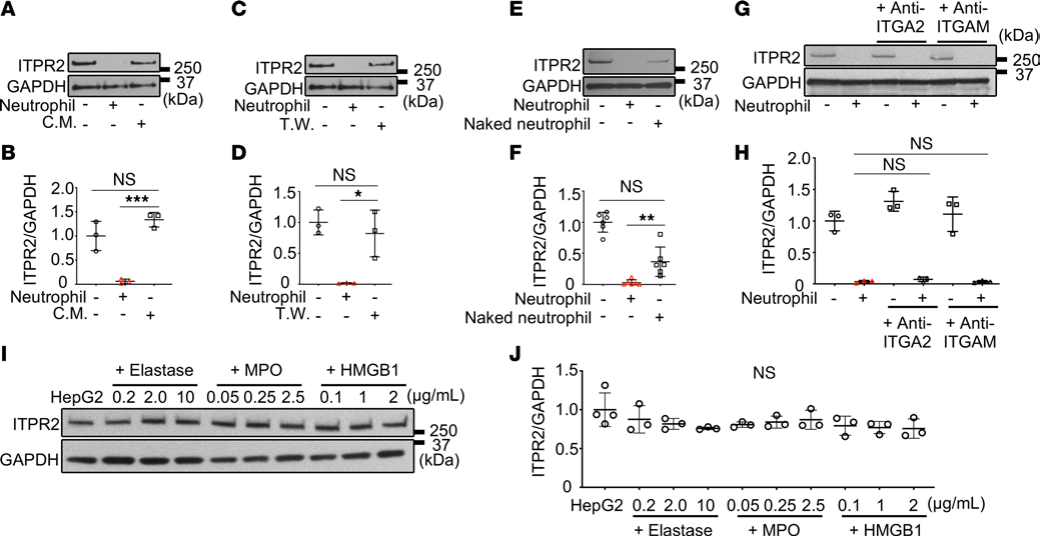
Direct contact between HepG2 cells and intact neutrophils is important for the decrease in ITPR2. (**A**) Representative immunoblots and (**B**) quantitation of ITPR2 levels after 18–24 hours of culture of HepG2 cell in conditioned medium (C.M.) with neutrophils cultured for 16 hours. C.M. does not reduce ITPR2 in HepG2 cells. (**C**) Representative immunoblots and (**D**) quantitation of ITPR2 in HepG2 cells separated from neutrophils by a 3-μm pore Transwell system (T.W.) after 18–24 hours. Coculture using T.W. does not decrease ITPR2 in HepG2 cells. (**E**) Representative immunoblots and (**F**) quantitation of ITPR2 levels in HepG2 cells after 18–24 hours of coculture with naked neutrophils, from which cell surface GPI-anchored proteins were removed. Naked neutrophils are less effective in reducing ITPR2 in HepG2 cells. (**G**) Representative immunoblots and (**H**) quantitation of ITPR2 levels in HepG2 cells after preincubation with anti–integrin α2 (anti-ITGA2) or anti–integrin αM (anti-ITGAM) antibodies for 2 hours and coculture with neutrophils for 18–24 hours. Inhibition of these integrins does not alter the decrease in ITPR2 in HepG2 cells. (**I**) Representative immunoblots and (**J**) quantitation of ITPR2 levels after incubation of HepG2 cells with various concentrations of neutrophil extracellular trap (NET) components (neutrophil elastase, myeloperoxidase [MPO], high mobility group box-1 [HMGB1]) for 20 hours. Extracellular administration of these NET components does not alter ITPR2 in HepG2 cells. Data are presented as mean ± SD; *n* = 6 (**F**), *n* = 3–4 (**J**), *n* = 3 (**B**, **D**, and **H**). NS, not significant. *P < 0.05, **P < 0.01, ***P < 0.001 by 1-way ANOVA with Tukey’s multiple-comparison test.

Several factors secreted by neutrophils have been implicated in liver diseases (26, 36). However, ITPR2 was not decreased in HepG2 cells treated with neutrophil-conditioned medium (Figure 3, A and B) or in cells separated from neutrophils by a semipermeable membrane (Figure 3, C and D). These results suggest neutrophils require direct contact with HepG2 cells to reduce ITPR2. To evaluate this, glycosylphosphatidylinositol-anchored (GPI-anchored) plasma membrane proteins were stripped from neutrophils using phosphatidylinositol-specific phospholipase C (PI-PLC, 0.5 units/mL) to generate “naked” neutrophils (37). The effect of naked neutrophils on ITPR2 was significantly attenuated compared with untreated neutrophils (Figure 3, E and F). Bulk RNA-seq was used to compare global gene expression in HepG2 cells with and without neutrophil coculture, and Ingenuity Pathway Analysis (IPA) revealed that neutrophils altered signaling related to cell adhesion and diapedesis in HepG2 cells (Supplemental Figure 3A). Integrin isoforms α2 and αM (ITGA2 and ITGM) belong to the top 5 IPA signaling pathways of cocultured HepG2 cells and can act as binding partners for neutrophils on some epithelia (37–39). However, blocking antibodies directed against these integrins did not prevent reduction of ITPR2 in HepG2 cells cocultured with neutrophils (Figure 3, G and H). An additional mechanism by which neutrophils come into direct contact with their targets is neutrophil extracellular traps (NETs), which can occur in liver (40). Typical mediators of NETs include high mobility group box 1 protein (HMGB1), neutrophil elastase, and myeloperoxidase (MPO) (41). However, extracellular administration of HMGB1, neutrophil elastase, or MPO to HepG2 cells had no effect on ITPR2 (Figure 3, I and J). Coculture for 4 hours with neutrophils in which NETs were induced by phorbol 12-myristate 13-acetate (PMA, 200 nM) also reduced ITPR2 in HepG2 cells (Supplemental Figure 3, B and C), but NET marker SYTOX Green was absent in neutrophils that were simply cocultured with HepG2 cells (Supplemental Figure 3, D and E). These results suggest that NET formation is not responsible for ITPR2 degradation here. Since NETs are also a form of neutrophil death (42), neutrophils treated with methanol to cause cell death were cocultured with HepG2 cells, but this also did not reduce ITPR2 (Supplemental Figure 3, F–I). Together, these findings suggest that intact neutrophils must be in direct contact with HepG2 cells to decrease ITPR2 by a mechanism that is independent of integrins or NET formation.

### Neutrophils insert granule proteins into hepatocytes.

**Figure 4:**
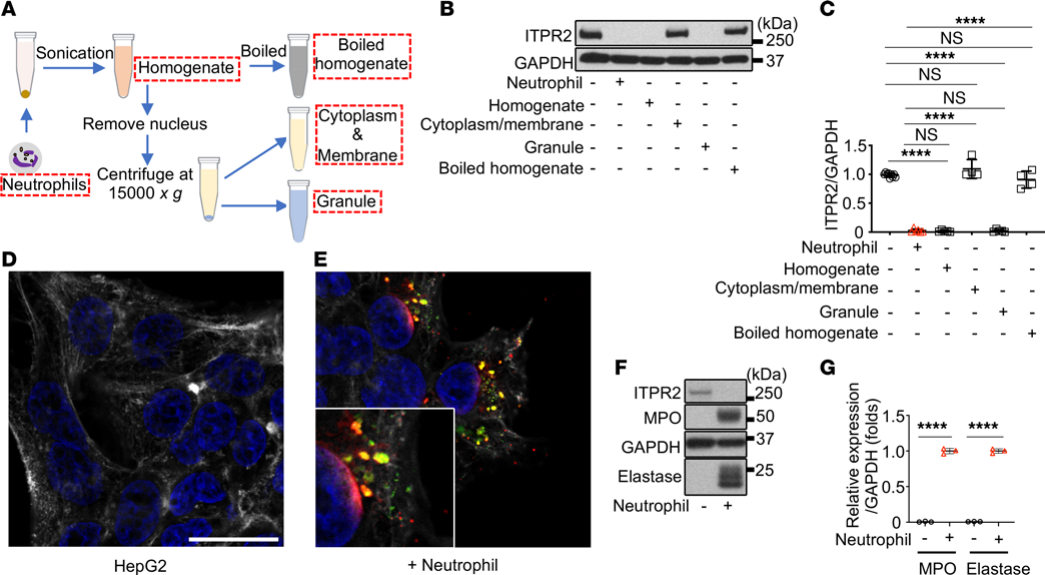
Neutrophils insert granule proteins into hepatocytes. (**A**) Flowchart showing the protocol for fractionating neutrophils. (**B**) Representative immunoblots and (**C**) quantitation of ITPR2 in HepG2 cells after 20 hours of incubation with the indicated fractions shows that the neutrophil granule fraction is sufficient to reduce ITPR2. (**D** and **E**) Representative confocal immunofluorescence images of HepG2 cells alone (**D**) and after coculture with neutrophils for 1 hour, after which the cells were then washed to remove the neutrophils (**E**). Labels are anti-myeloperoxidase (anti-MPO, green) and anti-elastase (red) antibodies, nuclear staining (DAPI, blue), and phalloidin (gray). Scale bar: 20 μm. Inset is an ×2 enlargement showing partial colocalization of MPO and elastase (yellow). Elastase also appears to be diffusely distributed in the nucleus. (**F**) Representative immunoblots and (**G**) quantitation show the neutrophil granule proteins MPO and elastase appear in HepG2 cells after coculturing with neutrophils for 1 hour, after which the cells were washed to remove the neutrophils. All data are presented as mean ± SD; *n* = 4–9 (**C**) and *n* = 3 (**G**). NS, not significant. ****P < 0.001 by 1-way ANOVA with Tukey’s multiple-comparison test.

To investigate the neutrophil component that reduces ITPR2 in hepatocytes, cellular fractions of neutrophils were administered to HepG2 cells (Figure 4A) (43). ITPR2 was decreased by incubation with either the granule-containing fraction or the total cell homogenate, but not by the cytoplasmic or plasma membrane fractions of neutrophils (Figure 4, B and C). Moreover, the effect of total homogenate was eliminated by boiling (Figure 4, B and C). The same results were obtained using primary human hepatocytes and human neutrophils (Supplemental Figure 4, A and B). These findings suggest proteins within neutrophil granules are transferred to hepatocytes upon direct contact. To test this hypothesis, HepG2 cells were cocultured with neutrophils for 1 hour, followed by washing to remove neutrophils. Confocal immunofluorescence revealed that the neutrophil granule proteins MPO and neutrophil elastase were present and partially colocalized within the HepG2 cells (Figure 4, D and E). This was confirmed by immunoblotting (Figure 4, F and G). Transfer of MPO and elastase from neutrophils also occurred in human and mouse primary hepatocytes, as verified by immunoblotting and immunostaining (Supplemental Figure 4, C–J).

### Hepatocyte ITPR2 is degraded by neutrophil elastase and hepatocytes express Serpin E2 and A3 in response.

**Figure 5:**
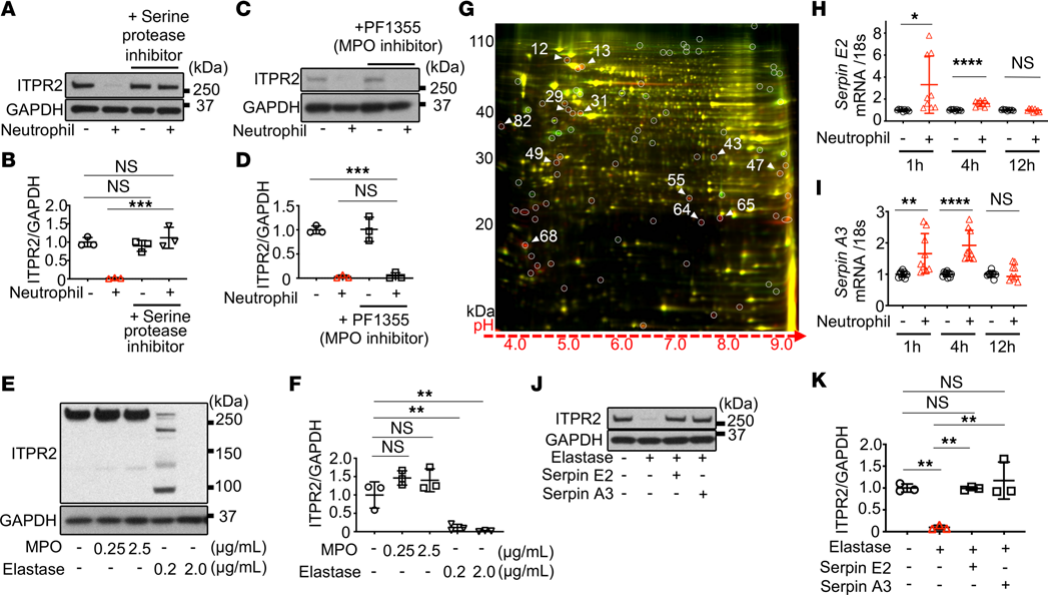
Hepatocyte ITPR2 is degraded by neutrophil elastase and hepatocytes express Serpin E2 and A3 in response. (**A**) Representative immunoblots and (**B**) quantitation of ITPR2 levels in HepG2 cells after coculture with neutrophils for 1 hour show that loss of ITPR2 is blocked by the serine protease inhibitor AEBSF (10 μM). (**C**) Representative immunoblots and (**D**) quantitation of ITPR2 levels in HepG2 cells after coculturing with neutrophils for 1 hour show that loss of ITPR2 is not blocked by the MPO inhibitor PF-1355 (10 μM). (**E**) Representative immunoblot and (**F**) quantitation of ITPR2 levels in HepG2 cell lysates 5 minutes after addition of MPO or neutrophil elastase at the indicated concentrations. Note the presence of multiple low molecular weight bands in homogenates treated with a low concentration of elastase, but ITPR2 is completely digested in homogenates treated with a higher concentration. (**G**) 2D-DIGE of a representative subproteome from lysates of HepG2 cells with (red, Cy5) or without (green, Cy2) neutrophil elastase (1 mg/mL). Yellow spots indicate overlap of both samples. Eighty-two spots were detected with a ratio of green to red fluorescence of greater than 1.5 (surrounded by circles), likely reflecting significantly degraded proteins. The 12 spots with the greatest differences (spots at the edges and spots near each other were avoided) were analyzed by mass spectrometry (Table 1). (**H**) RT-qPCR shows *Serpin E2* mRNA is increased at early time points in HepG2 cells cocultured with neutrophils, compared with HepG2 cells alone. (**I**) RT-qPCR shows mRNA levels of *Serpin A3* also are increased at early time points in HepG2 cells cocultured with neutrophils, compared with HepG2 cells alone. (**J**) Representative immunoblots and (**K**) quantitation of ITPR2 levels in HepG2 cell lysates 5 minutes after addition of neutrophil elastase protein (0.2 μg/mL) show that degradation of ITPR2 can be prevented by either recombinant Serpin E2 or Serpin A3 protein (20 μg/mL). All data are *n* = 3 and are presented as mean ± SD. NS, not significant. *P < 0.05; **P < 0.01; ***P < 0.001; ****P < 0.0001 by 1-way ANOVA with Tukey’s multiple-comparison test (**B**, **D**, **F**, and **K**) or unpaired, 2-tailed Student’s *t* test (**H** and **I**).

In HepG2 cells cocultured with neutrophils, the decrease in ITPR2 was blocked by 4-(2-aminoethyl) benzenesulfonyl fluoride hydrochloride (AEBSF), a serine protease inhibitor, but not by an MPO inhibitor (Figure 5, A–D). Administration of MPO to HepG2 cell homogenates rather than intact cells (Figure 3I) did not reduce ITPR2, but elastase at 0.2 μg/mL laddered the ITPR2 bands and eliminated them at 2.0 μg/mL (Figure 5, E and F). These results suggest neutrophil elastase is transferred to HepG2 cells by direct contact with neutrophils to degrade ITPR2. To examine the range of proteins degraded by neutrophil elastase (Figure 1K), proteomic analysis was performed using 2-dimensional difference gel electrophoresis (2D-DIGE) (44). Comparing HepG2 lysates with and without neutrophil elastase treatment, only 82 of 2153 spots were degraded by elastase (Figure 5G). Mass spectrometry identification of the 12 most degraded proteins is shown in Table 1. Infiltration of neutrophils occurs in most organs (22), so transfer of neutrophil granule proteins and ITPR2 degradation by elastase was examined in other cell types. In HCT116 (colon), A549 (lung), and PANC-1 (pancreas) cells cocultured with neutrophils for 1 hour, the presence of MPO and elastase was observed along with a decrease in ITPR2 (Supplemental Figure 5, A and B). Cathepsin G and MMP9, two other neutrophil granule proteins (45), could not be detected in HepG2 cells even after coculture with neutrophils (Supplemental Figure 5C). The observations that neutrophils do not damage hepatocytes in this system (Figure 1, A–E, and Supplemental Figure 1, B–K) and that removal of neutrophils restores ITPR2 (Figure 2, E–G) suggest hepatocytes have a mechanism to temporally limit this effect of neutrophils. To investigate this, bulk RNA-seq was performed using HepG2 cells 20 hours after incubation with either neutrophils or the neutrophil granule fraction (Supplemental Figure 5, D and E). We identified 101 upregulated and 108 downregulated genes by neutrophils, plus 50 upregulated and 49 downregulated genes by the granule fraction, respectively (Supplemental Figure 5F). Ten genes were downregulated by both neutrophils and granules, including *Serpin E2* and *Serpin A3* (Supplemental Figure 5F), 2 known antiprotease genes (46). Because proteolysis by neutrophil elastase in hepatocytes occurs as early as 1 hour after coculture, RT-qPCR was used to evaluate *Serpin E2* and *Serpin A3* gene expression in HepG2 cells at time points preceding 20 hours. Both mRNAs in HepG2 cells were significantly elevated at 1 and 4 hours of coculture with neutrophils (Figure 5, H and I), suggesting that HepG2 cells rapidly elevated *Serpin E2* and *Serpin A3* in response to neutrophil elastase, with a compensatory decrease in these mRNAs after 20 hours. Serpin A3 protein level also was elevated in human hepatocytes cocultured with neutrophils after 1 hour (Supplemental Figure 5, G and H). Finally, administration of recombinant Serpin E2 or Serpin A3 to HepG2 cell homogenates inhibited ITPR2 degradation by neutrophil elastase (Figure 5, J and K). These findings provide evidence that hepatocytes respond to contact with neutrophils by increasing expression of Serpin E2 and Serpin A3 to temporally limit the degradative effects of neutrophil elastase.

### Granules containing MPO and elastase are taken up by hepatocytes by PI3K-mediated endocytosis.

**Figure 6:**
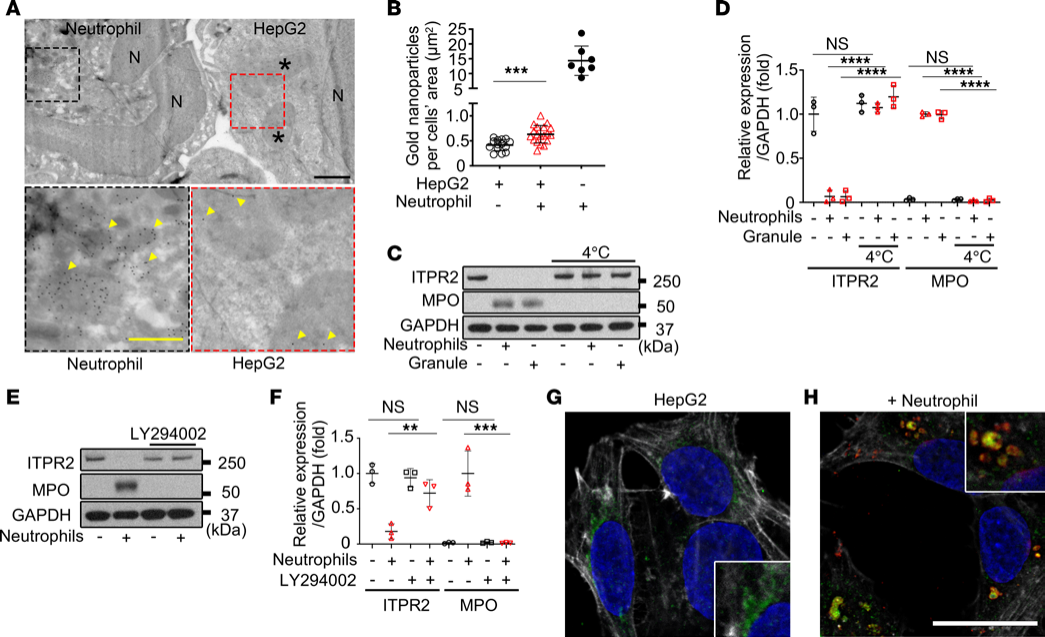
Granules containing MPO and elastase are taken up by hepatocytes via PI3K-mediated endocytosis. (**A**) Neutrophil granules are seen in HepG2 cells by transmission electron microscopy. Top panel: Representative image of HepG2 cells cocultured with neutrophils (N, nucleus; black asterisks, endosome-like structures in HepG2 cells). Scale bar: 1 μm. Bottom left panel: Magnified image shows gold nanoparticles (yellow arrows) bound to MPO antibodies in neutrophil granules. Scale bar: 500 nm. Bottom right panel: Magnified image shows MPO within endosome-like structures in HepG2 cells (yellow arrows). (**B**) Immunogold labeling is increased in HepG2 cells cocultured with neutrophils. Shown is the number of gold nanoparticles in HepG2 cells cultured alone or with neutrophils, or in neutrophils alone, each normalized by cell area. Data are from 14 images of HepG2 cells alone, 19 images of HepG2 cells cocultured with neutrophils, and 7 images of neutrophils alone. (**C**) Representative immunoblots and (**D**) quantitation of ITPR2 and MPO in HepG2 cells incubated with neutrophils or neutrophil granule fractions for 1 hour at 4°C or 37°C show that transfer of MPO and ITPR2 degradation are blocked at 4°C. (**E**) Representative immunoblots and (**F**) quantitation of ITPR2 and MPO in HepG2 cells incubated with LY294002 (40 μM) for 1 hour and then cocultured with neutrophils for 1 hour show that transfer of MPO and ITPR2 degradation are blocked by the PI3K inhibitor. (**G**) Representative confocal images of HepG2 cells alone and (**H**) after coculture with neutrophils for 1 hour. Labels are anti-LAMP1 (green) and anti–neutrophil elastase (red) antibodies, Hoechst 3342 (blue), and phalloidin (gray). Scale bar: 20 μm. Note that in cocultured HepG2 cells, some LAMP1 colocalizes with neutrophil elastase (yellow; better appreciated in ×2 magnification in inset). All data are presented as mean ± SD; *n* = 3 (**D** and **F**). NS, not significant. **P < 0.01; ***P < 0.001; ****P < 0.0001 by unpaired, 2-tailed Student’s *t* test (**B**) or 1-way ANOVA with Tukey’s multiple-comparison test (**D** and **F**).

To investigate how neutrophils transfer granule proteins to HepG2 cells, immunoelectron microscopy was performed using gold nanoparticles conjugated with an anti-MPO antibody. Quantitative analysis of immunoelectron labeling showed that MPO in neutrophil granules migrates to HepG2 cells, where it was localized to an endosome-like structure (Figure 6, A and B). This endosome-like structure was in the cytoplasm of HepG2 cells cocultured with neutrophils, but not in control HepG2 cells (Supplemental Figure 6, A and B). Although this structure was larger than neutrophil granules, immunostaining showed that MPO partially colocalized with elastase, suggesting they migrate from neutrophils to hepatocytes in granules or vesicles (Figure 4E). Therefore, after removing cocultured neutrophils from HepG2 cells, the HepG2 cells were subjected to the same procedure that was used to isolate the granule fraction from neutrophils (Figure 4A). This resulted in recovery of a granule fraction from HepG2 cells that contained both MPO and elastase (Supplemental Figure 6C). Uptake of granule proteins and ITPR2 degradation could be inhibited by incubation at 4°C or treatment with PI3K inhibitors, LY294002 and wortmannin (Figure 6, C–F, and Supplemental Figure 6, D and E), suggesting granules are transferred by PI3K-mediated endocytosis. Colocalization of lysosome-associated membrane protein 1 (LAMP1) with neutrophil elastase in HepG2 cells cocultured with neutrophils (Figure 6, G and H) supports the notion that granule uptake is by the endocytosis pathway. Furthermore, in HepG2 cells after incubation of the neutrophil granule fraction with the lipophilic membrane dye PKH67 (47), the colocalization of elastase (Supplemental Figure 6, F and G) or LysoTracker (Supplemental Figure 6, H and I) with this lipophilic membrane dye also supports these results. Some of the neutrophil elastase in HepG2 cells did not colocalize with MPO (Figure 4E), appearing to diffuse into the nucleus (Figure 4E and Supplemental Figure 6G), which suggests the elastase is transported out of the internalizing granules. This phenomenon can occur when NETs are elicited in neutrophils and may be associated with MPO-induced reactive oxygen species (48). Administration of the antioxidant *N*-acetyl-L-cysteine (NAC) inhibited the degradation of ITPR2 (Supplemental Figure 6, J and K), which may suggest the possibility of a similar mechanism.

### Loss of ITPR2 and impaired hepatocyte proliferation are attenuated in an elastase-deficient mouse model of AAH.

**Figure 7:**
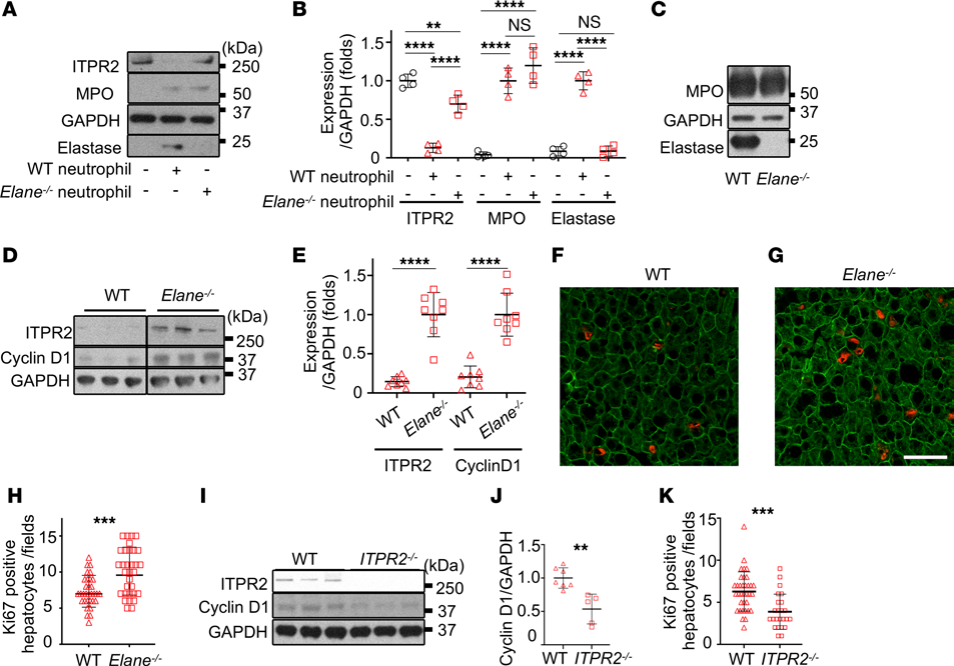
Loss of ITPR2 and impaired hepatocyte proliferation are attenuated in an elastase-deficient mouse model of alcohol-associated hepatitis (AAH). (**A**) Representative immunoblots and (**B**) quantitation of ITPR2, MPO, and elastase in primary mouse hepatocytes after 1 hour of coculture with neutrophils from WT or neutrophil elastase–KO (*Elane^–/–^*) mice. Findings support the notion that granules are transferred to hepatocytes from either type of neutrophil, but ITPR2 is only reduced if elastase is present. (**C**) Representative immunoblots confirm expression of MPO but not elastase in bone marrow of mice transplanted with *Elane^–/–^* or WT bone marrow, respectively. (**D**–**H**) In AAH models, ITPR2 levels, which are reduced in WT mice, improve in *Elane^–/–^* mice and hepatocyte proliferation increases. AAH was induced by the ethanol + LPS + fructose (ELF model; ref. 41) in *Elane^–/–^* or WT mice. (**D**) Representative immunoblots and (**E**) quantitation of ITPR2 and cyclin D1 in liver homogenates show that both are increased in *Elane^–/–^* mice. The lanes were on the same gel but noncontiguous. Representative confocal images of (**F**) WT and (**G**) *Elane^–/–^* livers immunostained with anti-CK18 (green) and anti-Ki67 (red) antibodies. Scale bar: 50 μm. (**H**) Quantitative comparison confirms the number of Ki67-positive hepatocytes is greater in *Elane^–/–^* mice. (**I**–**K**) In the AAH (ELF) model, hepatocyte proliferation is decreased in ITPR2-deficient (*ITPR2^–/–^*) mice compared with WT mice. (**I**) Representative immunoblots and (**J**) quantitation of the levels of ITPR2 and cyclin D1 in liver homogenates show a reduction of cyclin D1 in *ITPR2^–/–^* mice. (**K**) Quantitation of immunostaining of livers with anti-CK18 and anti-Ki67 antibodies shows that the number of Ki67-positive hepatocytes is reduced in the KO mice. All data are expressed as mean ± SD; *n* = 4 (**B**), *n* = 8 (**E**), 4 fields of view per mouse (**H**), *n* = 7 for WT and *n* = 5 for *ITPR2^–/–^* (**J**), and 4–5 fields of view per mouse (**K**). NS, not significant. **P < 0.01; ***P < 0.001; ****P < 0.0001 by 1-way ANOVA with Tukey’s multiple-comparison test (**B**) or unpaired, 2-tailed Student’s *t* test (**E**, **H**, **J**, and **K**).

Ethanol-LPS-fructose (ELF) treatment was used as a mouse model of AAH (37) and it showed that ITPR2 levels in liver homogenates were significantly reduced compared with controls (Supplemental Figure 7, A and B). The number of neutrophils infiltrating the liver was significantly increased as well (Supplemental Figure 7, C–E). *Serpin E2* mRNA of ELF-treated liver also increased compared with controls (Supplemental Figure 7F). The chronic-plus-binge AAH model (49) also resulted in decreased liver ITPR2 levels compared with controls (Supplemental Figure 7, G and H). There was also an increase in infiltrating neutrophils over controls in this model, but it was less pronounced than in the ELF model (Supplemental Figure 7I). These results indicate there is neutrophilic infiltration and decreased ITPR2 in the livers of AAH model mice. To clarify the relationship between neutrophil elastase and ITPR2 degradation in vivo, protein levels of primary mouse hepatocytes were compared after 1 hour of coculture with neutrophils isolated from neutrophil elastase–knockout (*Elane^–/–^*) or wild-type (WT) mice. MPO was found in both hepatocytes, but *Elane^–/–^* neutrophils failed to degrade ITPR2 (Figure 7, A and B). Next, the ELF model was examined in *Elane^–/–^* and WT mice (Figure 7C). ITPR2 levels in the livers of *Elane^–/–^* mice were significantly increased compared with WT, as was the hepatocyte proliferation marker cyclin D1 (Figure 7, D and E) (26). The number of hepatocytes expressing Ki67, another cell proliferation marker, was also significantly higher in *Elane^–/–^* than WT mice (Figure 7, F–H). In WT ELF-treated mice, treatment with the neutrophil elastase–specific inhibitor sivelestat or with AEBSF also increased the number of Ki67-positive hepatocytes (Supplemental Figure 7, J–M), and the levels of ITPR2 and cyclin D1 in the liver also were significantly increased (Supplemental Figure 7, N–O). Finally, serum ALT levels were higher in *ITPR2^–/–^* ELF mice than in matched WT ELF mice (145 ± 42 vs. 101 ± 26 U/L, *P* = 0.02, one-tailed Student’s *t* test) and lower in *Elane^–/–^* ELF mice than in matched WT ELF mice (56 ± 8 vs. 81 ± 24 U/L, *P* = 0.006, one-tailed Student’s *t* test). Collectively, these results provide evidence that ITPR2 in hepatocytes is degraded, and proliferation is reduced by elastase from infiltrating neutrophils in the ELF model of AAH. Finally, the livers of *ITPR2^–/–^* ELF mice had significantly lower levels of cyclin D1 and significantly fewer Ki67-positive hepatocytes compared with WT ELF mice (Figure 7, I–K). This is consistent with previous reports that decreased hepatocyte proliferation is related to ITPR2 deficiency (18) and extends this to AAH.

### Degradation of ITPR2 in hepatocytes occurs by insertion of neutrophil elastase in patients with AAH.

**Figure 8:**
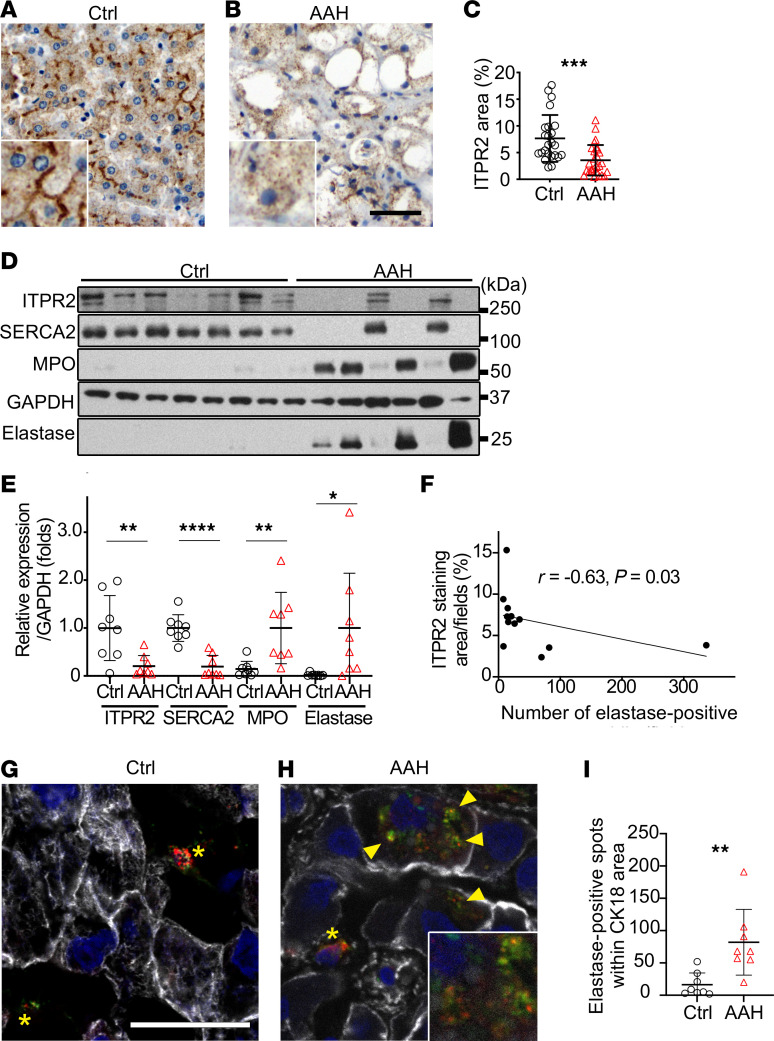
Degradation of ITPR2 in hepatocytes occurs by insertion of neutrophil elastase in patients with alcohol-associated hepatitis (AAH). (**A**–**C**) ITPR2 is reduced in hepatocytes in patients with AAH. Representative images of immunohistochemical staining with anti-ITPR2 antibody in (**A**) histologically normal controls (Ctrl) and (**B**) liver biopsy specimens from patients with AAH. Inset in each image is magnified ×3. Scale bar: 50 μm. (**C**) Quantitative measurement of ITPR2 staining confirms decreased expression in AAH. Three fields were quantified in each biopsy specimen (8 Ctrl and 9 AAH). (**D**–**F**) There is an inverse relationship between ITPR2 in hepatocytes and neutrophil elastase in liver biopsies from patients with AAH. (**D**) Representative immunoblots and (**E**) quantitation of ITPR2, SERCA2, MPO, and elastase in homogenates of livers from 8 Ctrl and explants from 8 patients who received liver transplants for AAH. ITPR2 and SERCA2 are decreased further in patients with more neutrophil infiltration. (**F**) There is an inverse relationship between ITPR2 and neutrophil elastase staining (*r* = –0.63, Spearman’s correlation; *P* < 0.05) based on the 12 individuals who underwent both analysis of the immunostained region of ITPR2 (**A**–**C**) and the number of elastase-stained positive neutrophils in panel (**D**–**F**) of Supplemental Figure 8. (**G**–**I**) Neutrophil elastase is found in hepatocytes of patients with AAH. Representative images of confocal fluorescent immunostaining with anti-CK18 (gray), anti-MPO (green), and anti-elastase (red) antibodies from (**G**) histologically normal human liver and (**H**) liver of AAH patient. Yellow asterisks represent neutrophils and arrowheads indicate that MPO and elastase dots are present within CK18-positive hepatocytes. Inset in **H** is an ×2 magnified image showing partial colocalization of MPO and elastase (yellow). Scale bar: 20 μm. (**I**) The number of spots of elastase present inside CK18-positive hepatocyte regions is significantly increased in AAH biopsies relative to controls. Ctrl = 8, AAH = 8. All data are presented as mean ± SD. *P < 0.05, **P < 0.01, ***P < 0.001 by 1-tailed Student’s *t* test (**C**) or unpaired, 2-tailed Student’s *t* test (**E**, **F**, and **I**).

To determine the relevance of these observations to human disease, liver biopsies from patient specimens that were histologically normal were compared to specimens from patients with biopsy-proven AAH. Immunohistochemical staining for neutrophil elastase and MPO showed a significant increase in the number of positive cells found in the liver parenchyma from patients with AAH compared with histologically normal controls (Supplemental Figure 8, A–F), while immunohistochemical staining for ITPR2 was significantly decreased in hepatocytes of biopsies from patients with AAH, relative to histologically normal controls (Figure 8, A–C). Protein levels were evaluated in homogenates of liver explants from patients transplanted for severe AAH, plus histologically normal livers (patient information in Table 2). Consistent with in vitro findings, ITPR2 and SERCA2 were significantly decreased and MPO and elastase were significantly increased in AAH compared with normal livers (Figure 8, D and E). Moreover, ITPR2 staining was inversely related to the number of elastase-positive cells (*r* = –0.63, *P* = 0.03; Figure 8F). Furthermore, higher magnification identified MPO- or elastase-positive granular staining in hepatocytes near neutrophils (Supplemental Figure 8, G and H), which was more apparent with fluorescent immunostaining for CK18 (hepatocytes), neutrophil elastase, and MPO in normal or AAH liver sections (Figure 8, G and H). In histologically normal specimens, neutrophil elastase and MPO were mostly confined to sinusoidal regions, whereas in AAH specimens, they appeared as punctate labels within CK18-positive cytoplasm, some of which colocalized. The number of elastase-positive puncta inside hepatocytes was significantly higher in AAH specimens than in controls (Figure 8I). Serpin A3 levels were significantly higher in homogenates of AAH liver samples than in controls (Supplemental Figure 8, I and J) as well. Finally, AAH specimens were fluorescently labeled for CK18, neutrophil elastase, and the apoptosis marker cleaved caspase 3 to detect perforocytosis (50). The occurrence of neutrophils in apoptotic hepatocytes was observed (Supplemental Figure 8K), but was rare.

### Neutrophils from patients with AAH are more potent than healthy controls in degrading ITPR2.

**Figure 9:**
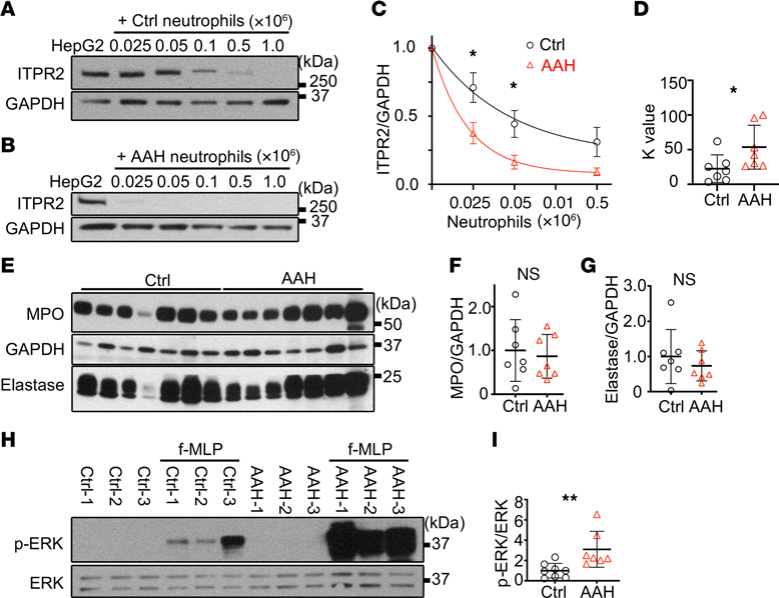
Neutrophils from patients with alcohol-associated hepatitis (AAH) are more capable than healthy controls of degrading ITPR2. (**A**–**D**) Kinetics of ITPR2 loss in HepG2 cells cocultured with control or AAH neutrophils. (**A**) Representative immunoblots of ITPR2 levels in HepG2 cells cocultured with neutrophils for 20 hours from (**A**) healthy volunteers or (**B**) patients with AAH. (**C**) Quantification of data from 7 healthy volunteers and 7 AAH patients shows a significant difference in measured ITPR2/GAPDH levels when 0.025 × 10^6^ or 0.05 × 10^6^ neutrophils were administered. (**D**) First-order rate constant *K* is higher for AAH neutrophils than for controls. (**E**–**G**) MPO and elastase content is similar in control and AAH neutrophils. (**E**) Representative immunoblots and (**F** and **G**) quantitation of MPO and elastase protein levels in neutrophils from 7 healthy volunteers and 7 AAH patients. (**H** and **I**) ERK signaling is more active in AAH neutrophils. (**H**) Representative immunoblots and (**I**) quantitation of p-ERK and ERK after stimulation of neutrophils isolated from blood of 8 healthy volunteers (Ctrl) or 7 AAH patients with fMLP (100 nM) for 5 minutes. Data are shown as mean ± SEM (**C** and **D**) or mean ± SD (**F**, **G**, and **I**). NS, not significant. *P < 0.05, **P < 0.01 by unpaired, 2-tailed Student’s *t* test.

The function of neutrophils in patients with AAH may differ from that of healthy donors (51, 52), and indeed we found that neutrophils from AAH patients (information in Table 3) were more capable than neutrophils from healthy individuals in degrading ITPR2 in HepG2 cells (Figure 9, A–D). MPO and elastase levels were similar in healthy and AAH neutrophils, suggesting that differences between the neutrophils are not due to increased expression of granule proteins (Figure 9, E–G). Therefore, ERK phosphorylation in response to stimulation with *N*-formyl-methionyl-leucyl-phenylalanine (fMLP) was evaluated as an index of neutrophil activation, including degranulation (53). Indeed, AAH neutrophils displayed increased ERK phosphorylation when compared with control neutrophils (Figure 9, H and I). These results suggest that neutrophils in AAH are primed (54) to transfer granule contents into hepatocytes, resulting in a more effective degradation of ITPR2.

## Discussion

Neutrophils control infection by killing bacteria using reactive oxygen species, granule proteins, NETs, and other substances (41). On the host side, it is commonly reported that neutrophil granule proteins also damage hepatocytes (4, 11). For example, when granule components are released extracellularly in NETs, they can damage epithelial systems, including hepatocytes (2). Additionally, when retinal or colon cancer cell lines or human umbilical vein endothelial cells are cocultured with neutrophils, MPO migrates into these cells to cause cytotoxicity (39, 55, 56). On the other hand, neutrophil elastase has been reported to modulate clock genes, insulin resistance, and lipogenesis in the liver, besides directly causing liver injury (6–8), but how elastase acts at the hepatocyte level has not been explained. The present study provides evidence that neutrophils inject granule contents into hepatocytes to use elastase as a signal transduction molecule rather than as a nonspecific cytotoxic protease, as seen in NETs.

It is particularly notable that neutrophils did not change their ALT, mitochondrial membrane potential, or albumin synthesis, and this was observed not only in HepG2 cells but in primary mouse and human hepatocytes as well. Also, viability of isolated neutrophils was prolonged when cocultured with hepatocytes, consistent with recent reports that tissue infiltration alters neutrophil phenotypes (22–24). Additional features of elastase as a signaling molecule in hepatocytes are that the hepatocytes respond by expressing Serpins as a recovery mechanism, and that ITPR2 and Ca^2+^ signaling are restored when the neutrophil leaves. The limited range of target proteins suggests other pathways in addition to those involving ITPR2 also may be selectively altered by neutrophil elastase. Granules containing MPO and elastase were inserted into hepatocytes by PI3K-mediated endocytosis, but further experiments are needed to investigate this in more detail because neutrophils must be in direct contact with hepatocytes rather than simply being engulfed by extracellular vesicles. Other granule proteins such as cathepsin G and MMP9 were not detected in hepatocytes despite the presence of MPO and elastase, suggesting some specificity in the transfer of granules or their contents to hepatocytes. Questions also remain as to how elastase moves from internalized granules to target proteins in hepatocytes. Because the targets of elastase are proteins of the cytoskeleton, ER, or nucleus rather than soluble cytoplasmic proteins, and because it localizes to lysosomes, a compartmentalized transport process may be responsible. In fact, in the hepatocyte cytoplasm, some elastase was visualized as puncta that did not colocalize with MPO, as well as diffusely in the nucleus. The mechanism by which elastase escapes from granules is also an interesting question. A similar phenomenon occurs by MPO-dependent reactive oxygen species when neutrophils undergo NET formation (48), and a similar mechanism may be occurring here because the antioxidant NAC inhibited the degradation of ITPR2.

**Figure 10:**
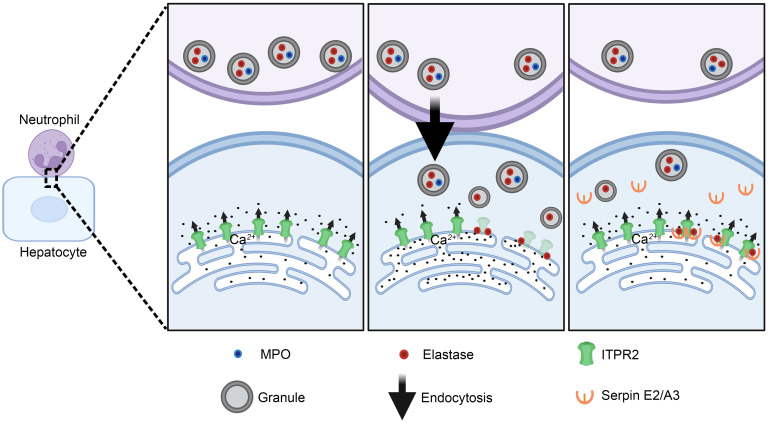
The sequence of events in neutrophil-hepatocyte interactions in AAH. Left: Neutrophils are in proximity to hepatocytes. Center: Neutrophils directly contact hepatocytes to transfer granules that contain MPO and elastase via PI3K-mediated endocytosis into the hepatocytes. The neutrophil elastase degrades ITPR2 and certain other proteins. Right: The neutrophil moves away from the hepatocyte, which recovers Ca^2+^ signaling in the hepatocyte by producing Serpin E2 and A3 to block the elastase.

Studies performed in vivo with mouse models and using specimens from patients with AAH suggest the clinical relevance of the current findings. This includes evidence that degradation of ITPR2 in hepatocytes by neutrophil elastase contributes to decreased hepatocyte proliferation in AAH mice, and that elastase deficiency or neutrophil elastase inhibitors can ameliorate this. AAH studies in ITPR2-deficient mice lent further support for a causal link for the association between ITPR2 in hepatocytes and hepatocyte proliferation. Moreover, serum ALT levels were inversely related to the degree of hepatocyte proliferation in the various mouse models of AAH. Degradation of ITPR2 in hepatocytes by transfer of neutrophil elastase also occurred in the livers of AAH patients, and AAH neutrophils were more potent in causing ITPR2 degradation than normal controls. These findings collectively suggest that infiltrating neutrophils and hepatocytes routinely engage in this mode of signaling (Figure 10), and that in AAH this balance is disturbed by a rapid increase in infiltrating neutrophils and enhanced granule secretory signaling, leading to a decrease in ITPR2. Several previous reports about the nondestructive effects of neutrophils on hepatocytes by mechanisms other than infection control lend support to the present findings (5, 22).

Ca^2+^ signaling regulates a wide range of cellular functions, and in liver and other epithelial organs this is mediated mostly by Ca^2+^ release from ITPRs (57). ITPR2 is the predominant isoform in hepatocytes (17), where it plays an important role in secretion by promoting targeting and canalicular insertion of BSEP and MRP2, two of the major transporters for bile secretion (19). ITPR2 also plays an important role in liver regeneration by promoting cell proliferation (18). This is because HGF, EGF, and insulin, which are the primary growth factors that promote liver regeneration, act by increasing Ca^2+^ in the nucleus (30), and proliferation of hepatocytes depends on increases in nucleoplasmic Ca^2+^ (29). Consequently, liver diseases in which ITPR2 is reduced result in impairments in both bile secretion and liver regeneration. Both bile secretion and liver regeneration are impaired in AAH as well (27, 37, 58, 59), consistent with our observation that ITPR2 also is reduced in this clinical condition. Cells employ a range of strategies to regulate their own levels of ITPRs to control their Ca^2+^ signals, including transcriptional and epigenetic mechanisms, plus a variety of degradative mechanisms (32, 35, 60–62). ITPR levels also can be regulated by neighboring cells. For example, ITPR3 is the dominant isoform in cholangiocytes (63), and its expression in these cells can be reduced by neutrophils, which in this case bind to integrins on the cholangiocyte membrane, leading to inhibition of ITPR3 transcription (37). The current work provides a different mechanism for neighboring cells to modulate ITPRs in target cells, by direct insertion of a degradative enzyme. The finding that neutrophils rapidly degrade Ca^2+^-related proteins and manipulate cell proliferation defines what we believe is a novel way for epithelial cells to spatially and temporally regulate Ca^2+^ signaling to adapt to changes in the tissue environment.

This study has certain limitations, which may guide future work. First, we did not examine phenotypical differences between neutrophils in the blood and neutrophils infiltrating the liver, raising the question of whether and how hepatocytes may contribute to the functional adaptation of neutrophils in this target tissue. This question may be particularly relevant in light of recent work proposing that neutrophils have a previously unrecognized degree of transcriptional plasticity that is likely modulated by their surrounding tissue environment. This concept may be particularly relevant in AAH, because the transcriptional profile of liver neutrophils in this condition differs from that of blood neutrophils in AAH, as well as from that of liver neutrophils in alcohol-associated cirrhosis (22, 64). Second, although neutrophil granules and their elastase are inserted into hepatocytes in a manner that depends on PI3K-mediated endocytosis, further details such as target tissue specificity are unclear. The observations that this insertion occurs in hepatocytes in vivo, and in other types of epithelial cells as well, suggest that this reflects a more general type of neutrophil–epithelial cell interaction that has not been recognized before. By identifying this previously unappreciated mechanism by which neutrophils alter hepatocyte function, the current work has the potential to provide new therapeutic targets in AAH.

## Methods

### Sex as a biological variable.

Our animal study exclusively examined male mice. Studies on humans involved mostly men and few women, due to the nature of the diseases under study. Due to difficulties in collecting sufficient human samples, we did not reach a sample size that accounts for sex differences. It is not known whether the results of the present study apply to females as well.

### Isolation of neutrophils from human blood and administration of reagents to neutrophils.

Blood samples were collected from patients at Yale-New Haven Hospital (YNHH) with a diagnosis of AAH in 2022–2023, with samples from healthy volunteers collected then as well. Neutrophils were isolated and purified using with PolymorphPrep (Proteogenix, AN1114683) according to the manufacturer’s instructions. For removal of GPI-anchored membrane proteins from neutrophils, cells were treated with PI-PLC (0.5 units/mL; Sigma-Aldrich, P5542) at 37°C for 30 minutes with gentle stirring (37). To stimulate ERK signaling, isolated neutrophils (5 × 10^6^) were treated for 5 minutes with fMLP (100 nM; Sigma-Aldrich, F3506) at 37°C in a humidified 5% CO_2_ atmosphere. Cells were then centrifuged at 350*g* for 5 minutes and samples for immunoblotting were prepared from the pellet. For evaluation of neutrophil NETs, cells were incubated with SYTOX Green (0.2 μM; Thermo Fisher Scientific, S7020) for 30 minutes and Hoechst 33342 (100 ng/mL) was added for live imaging. For induction of NETs, cells were incubated with 200 nM PMA for 4 hours. Neutrophils were treated with 100% methanol for 5 minutes, and cell death was confirmed by ethidium homodimer-1 (EthD-1) staining. More information on the isolation of neutrophils can be found in Supplemental Methods.

### Isolation of mouse neutrophils.

Femurs and tibias were harvested from euthanized mice, and the bone marrow was washed into a culture dish using a sterile syringe. Neutrophils were isolated according to the EasySep Mouse Neutrophil Enrichment Kit protocol (StemCell Technologies, 19762).

### Histological analysis of human liver specimens.

Archived liver biopsies from patients at YNHH with AAH between 2012 and 2014 were used. Histologically normal tissues were obtained from the peripheral portion of liver resections performed for metastatic colorectal cancer at YNHH from 2010 to 2017, and cases were identified by a review of pathology reports. Anti-ITPR2 (gift from Richard Wojcikiewicz, SUNY, Syracuse, New York, USA) or anti–neutrophil elastase (Abcam, ab131260) or anti-MPO (Thermo Fisher Scientific, RB373A0) antibodies were used for immunohistochemical staining by NovoLink polymer detection system (Leica Biosystems, RE7290-K). ITPR2 staining area was calculated with ImageJ (NIH) by setting the same circular area to eliminate bias due to the cut edge of the biopsy specimen. For detailed protocols for immunohistochemical staining, please refer to Supplemental Methods.

### Cell culture, coculture with neutrophils, reagents.

Human hepatocytes were obtained from the Liver Tissue Procurement and Distribution System of the NIH (University of Pittsburgh). Mouse primary hepatocytes were obtained from 8- to 12-week-old mice using collagenase perfusion, as previously described (65). HepG2 cells were obtained from ATCC. HCT116, A549, and PANC-1 cells were obtained from Ikki Sakuma (Chiba University, Chiba, Japan), Samir Gautam (Yale University), and Moitrayee Bhattacharyya (Yale University), respectively. One million cells were plated and used for experiments the following day, unless otherwise noted. The ratio of neutrophils to cocultured adherent cells was 1:1 unless otherwise noted. For more information on how each cell was cultured and the reagents administered, see Supplemental Methods.

### Immunoblotting analysis.

Liver homogenate by sonication or cells were lysed in RIPA buffer plus protease and phosphatase inhibitor cocktail (Thermo Fisher Scientific, 89901 and 78440). Intensities of bands on immunoblots were quantified by densitometry analysis by ImageJ software and normalized to GAPDH. The phosphorylation signal of ERK was standardized by total ERK. For a detailed protocol of immunoblotting analysis and more information on the antibodies used, see Supplemental Methods.

### Neutrophil fractionation.

Neutrophils were fractionated as previously described (43). The homogenate fraction was obtained by centrifuging neutrophils in the medium at 400*g* for 5 minutes and treating the pellet with a stick ultrasound device (Fisherbrand CL18) at 120 W, 20 kHz output for 3 seconds, 3 times. This was subjected to centrifugation at 720*g* for 5 minutes and the settled pellet (debris and nuclei) was discarded. The supernatant was centrifuged at 15,000*g* for 10 minutes. The pellet was used as the granule fraction and the supernatant as the cytoplasmic or membrane fractions. Immunoblotting confirmed this crude granule fraction for the presence of MPO and elastase, but not GAPDH.

### Cell proliferation assays.

Cell proliferation was assessed using Click-iT EdU Alexa Fluor 488 reagents (Invitrogen, C10637) according to the manufacturer’s instructions for image-based detection. Five to 6 pictures were taken per coverslip using a Zeiss Axio Observer falling-fluorescence microscope with a 20× objective lens. Overall cell numbers were measured by nuclear staining with Hoechst 33342 and the percentage of cells incorporating EdU was assessed. For a detailed protocol using EdU Alexa Fluor 488 reagents, see Supplemental Methods.

### Cell live/dead viability assay.

To measure changes in viability of neutrophils and hepatocytes in coculture, a LIVE/DEAD Viability/Cytotoxicity kit (Molecular Probes, L3224) was used according to the manufacturer’s instructions. Five to 6 images per coverslip were acquired using a Zeiss Axio Observer falling-fluorescence microscope with a 20× objective. Calcein-AM (green) represents live cells and EthD-1 (red) represents dead cells.

### JC-1 assay for mitochondrial membrane potential.

Cells were cultured in 96-well plates and incubated with JC-1 dye (10 μg/mL; Thermo Fisher Scientific, T3168) at 37°C for 10 minutes, which was then detected using a microplate reader (excitation/emission, red, 550/600 nm, green, 485/535). CCCP (100 μM Cayman Chemical, 25458) was administered as a negative control.

### Neutrophil-conditioned medium and neutrophil coculture in a Transwell system.

Human neutrophils were cultured in Eagle’s modified essential medium (EMEM) at 37°C for 16 hours, and the supernatant was incubated with HepG2 cells for 18–24 hours as neutrophil-conditioned medium. Neutrophils were placed in the upper compartment of a 3-μm pore Transwell system (FALCON, 353096) and HepG2 cells in the lower compartment and cocultured for 18–24 hours.

### Functional blocking interventions.

To inhibit the function of ITGAM or ITGA2 in HepG2 cells, an anti-ITGAM antibody (Thermo Fisher Scientific, 14-0112-82, clone M1/70) or anti-ITGA2 antibody (Santa Cruz Biotechnology, sc-53502, clone P1E6) was used as previously described (66). Briefly, HepG2 cells were preincubated with ITGAM antibody (5 μg/mL) or ITGA2 antibody (10 μg/mL) for 2 hours at 37°C. After antibodies were removed by washing with PBS, the cells were cocultured for 18 hours with or without neutrophils.

### Confocal fluorescence imaging of hepatocytes.

Hepatocytes plated on glass coverslips were cocultured with neutrophils for 1 hour, fixed (4% paraformaldehyde, PFA), permeabilized (0.5% Triton X-100), blocked (PBS containing 5% bovine serum albumin and 0.05% Tween), and incubated overnight at 4°C with primary antibodies (1:200 dilution). Images were acquired using a Leica Stellaris 8 (Leica Microsystems). For more information on antibodies and staining, see Supplemental Methods.

### Electron microscopy.

HepG2 cells after coculture with or without neutrophils for 1 hour on coverslips were fixed and dehydrated through ethanol. The hardened blocks were cut using a Leica UltraCut UC7. Sixty-nanometer sections were collected on Formvar-/carbon-coated grids and stained using 2% uranyl acetate and lead citrate. The sections were viewed in a FEI Tecnai Biotwin TEM at 80 kV and images were acquired using an AMT NanoSprint15 MK2 sCMOS camera. For immunoelectron microscopy, the sample pellet was fixed and resuspended in 10% gelatin, spun down and chilled blocks, frozen rapidly in liquid nitrogen, were cut on a Leica Cryo-EMUC6 UltraCut and 60-nm-thick sections were collected using the Tokuyasu method (67). Immunolabeling of the ultrathin sections was performed according to Slot and Geuze (68) (anti-MPO, 1:100; Protein A gold, Utrecht Medical Center). The 60-nm sections were viewed in a FEI Tecnai Biotwin TEM at 80 kV and images were acquired using an AMT NanoSprint15 MK2 sCMOS camera. The number of MPO-bound gold nanoparticles entering HepG2 cells, cocultured with or without neutrophils, was counted and compared by dividing by the area of HepG2 cells. More information can be found in Supplemental Methods.

### Ca^2+^ signaling.

Fluorescence intensity of the Ca^2+^-sensitive fluorophore Fluo-4, AM (Invitrogen, F14201) in HepG2 cells responding to ATP was monitored using a Zeiss LSM 710 confocal microscope. Analysis was performed using ImageJ in selected regions of interest, and Ca^2+^ signals were compared by calculating the area under the curve after stimulation with ATP. Additional details can be found in Supplemental Methods.

### Granule staining and cell uptake.

To examine the uptake of the granule fraction of neutrophils by HepG2 cells, neutrophil-derived granule fractions (3.0 × 10^6^ cells) were stained according to the protocol of the PKH67 Green Fluorescent Cell Linker Mini Kit (Sigma-Aldrich, MINI67-1KT) and incubated with HepG2 cells for 1 hour before observation with a Leica Stellaris 8 confocal microscope. Simultaneous staining with LysoTracker Red DND-99 (Thermo Fisher Scientific, L7528) was captured by live imaging. Fluorescent immunostaining with an anti–neutrophil elastase antibody was also performed after fixation.

### Endocytosis inhibition experiments.

To inhibit endocytosis, experiments were performed at 4°C as previously reported (69). Plates of HepG2 cells were placed on ice (4°C) in an incubator for 30 minutes and cocultured with neutrophils or neutrophil granule fractions for an additional hour before HepG2 cells were collected. HepG2 cells pretreated with LY294002 (40 μM; Cayman Chemical, 70920) or wortmannin (20 μM; Cayman Chemical, 10010591) for 1 hour were further cocultured with neutrophils for 1 hour, after which the HepG2 cells were harvested.

### RNA isolation and quantitative real-time PCR.

Total RNA was extracted and purified by RNeasy Mini Kits (QIAGEN, 74104) according to the manufacturer’s protocol. One-half microgram of total RNA was reverse transcribed into cDNA using an iScript cDNA Synthesis kit (Bio-Rad, 1708891). All TaqMan primers and probes were obtained from Thermo Fisher Scientific: *ITPR2* (Hs00181916_m1), *Cxcl1* (Hs00236937-m1), *Serpin E2* (Hs00299953_m1), *Serpin A3 (*Hs00153674_m1), *18S* ribosomal RNA (rRNA, Hs03003631_g1), *Serpin E2* (Mm00436753_m1), and *β-Actin* (Mm00607939_s1). Real-time PCR reactions were performed in triplicate using an ABI 7500 Sequence Detection System (Applied Biosystems). The expression of target genes was normalized to *18S* rRNA or *β-Actin* and quantification of relative expression was determined.

### Bulk RNA-seq and IPA.

cDNA and library preparation and sequencing, IPA, and bulk RNA-seq data analysis were performed as previously described (37) and are described in detail in Supplemental Methods.

### Administration of proteins to HepG2 cell homogenates.

HepG2 cells were homogenized in 100 μL of PBS with a stick ultrasound device at 120 W, 20 kHz output for 3 seconds, 3 times. Neutrophil MPO, neutrophil elastase, recombinant proteins Serpin E2 (BioLegend, 769002) and Serpin A3 (R&D Systems, 1295-PI) were administered for 5 minutes at indicated concentrations.

### Mouse models of AAH.

Two separate mouse models of AAH were used, as well as the *Elane^–/–^* mouse and the *ITPR2^–/–^* mouse. Experiments with neutrophil elastase inhibitors followed previously published protocols (70, 71). Complete details can be found in Supplemental Methods.

### Measurement of ALT.

The enzymatic activity of ALT in the medium of cultured cells or serum of mice was measured in a microplate reader according to the instructions for the Alanine Transaminase Activity Assay Kit (Fluorometric) (Abcam, ab105134).

### Proteomic analysis and mass spectroscopy.

Proteomic analysis (2D-DIGE) of HepG2 cell lysates degraded by neutrophil elastase and protein identification by mass spectrometry were performed by Applied Biomics as previously described (44). More information can be found in Supplemental Methods.

### Fluorescent staining of liver.

Liver blocks of frozen human tissue or harvested mouse liver lobes were embedded in optimal cutting temperature (OCT) compound (Electron Microscopy Sciences, 72592). These sections were cut into serial sections (7 μm) in a cryostat and placed on glass slides. After fixation (4% PFA), permeabilization (0.5% Triton X-100), and blocking (5% normal goat serum + PBS), they were reacted overnight at 4°C with the following primary antibodies (1:200 dilution): anti-MPO, anti-elastase, anti-CD31 (BD Biosciences, 550274), anti-Ki67 (Thermo Fisher Scientific, 14-5698-82), anti-CK18 (Proteintech, 10830-1-AP or R&D Systems, AF 7619), and anti–cleaved caspase 3 (Cell Signaling Technology, 9661). Secondary antibodies were goat anti-mouse Alexa Fluor 555, goat anti-rabbit Alexa Fluor 488, and donkey anti-sheep Alexa Fluor 647 (Invitrogen, A21448) at a 1:500 dilution. Images were acquired using a Leica Stellaris 8.

### Statistics.

Data are expressed as mean ± SD or SEM of multiple independent experiments unless stated otherwise. All paired or unpaired Student’s *t* tests were 2-tailed except where otherwise noted. Comparisons between 3 or more groups were performed using 1-way ANOVA and Tukey’s multiple-comparison test. Differences with a *P* value of less than 0.05 were considered statistically significant. All statistical analyses were performed using GraphPad Prism 7 software.

### Study approval.

This study was conducted under protocols HIC-2000025846 and HIC-1304011763 and approved by the Institutional Review Board on the Protection of the Rights of Human Subjects (Yale University). Some tissue collections were approved by the Johns Hopkins Medicine Institutional Review Board (IRB00107893 and IRB00154881). Written informed consent was obtained from all participants. The study protocol conformed to the ethical guidelines of the Helsinki Declaration and the Istanbul Declaration. All animals received humane care in accordance with the NIH *Guide for the Care and Use of Laboratory Animals* (National Academies Press, 2011). Animal studies were approved by Yale University’s Institutional Animal Care and Use Committee (07602-2024 and 11377-2022).

### Data availability.

The data used in the analysis can be found in the Supporting Data Values file. The raw RNA-seq data have been deposited in the international NCBI SRA database as BioProject PRJNA876711 and PRJNA876578.

## Author contributions

NO planned and conducted most experiments, analyzed the data, and wrote the first draft of the manuscript. MFL participated in some of the Ca^2+^ signaling experiments. MTG supported the study with manuscript revisions and microscopy experiments. EK performed experiments on human hepatocytes. HA performed RNA-seq analysis. DAH assisted with and supervised the analysis of the RNA-seq data. TI performed bone marrow transplants in mice, and JPP planned and supported these experiments. BJP managed and retrieved patient information (age, sex, various laboratory tests) on frozen liver sections of AAH patients, and ZS helped plan and support this. BEE provided useful discussions about degradation of ITPRs and edited the manuscript. MHN designed the research, participated in data analysis and interpretation, provided all funding that supported this work, and edited the manuscript.

## Supplementary Material

Supplemental data

Unedited blot and gel images

Supporting data values

## Figures and Tables

**Table 1:**
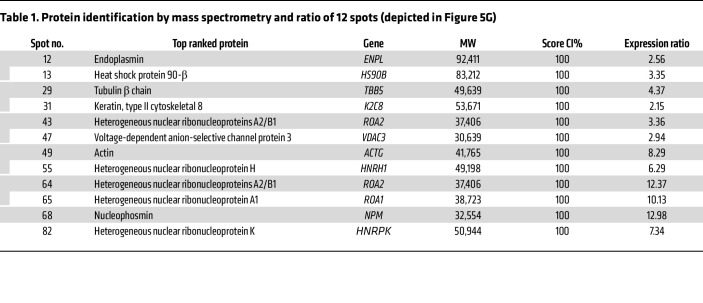
Protein identification by mass spectrometry and ratio of 12 spots (depicted in Figure 5G)

**Table 2:**
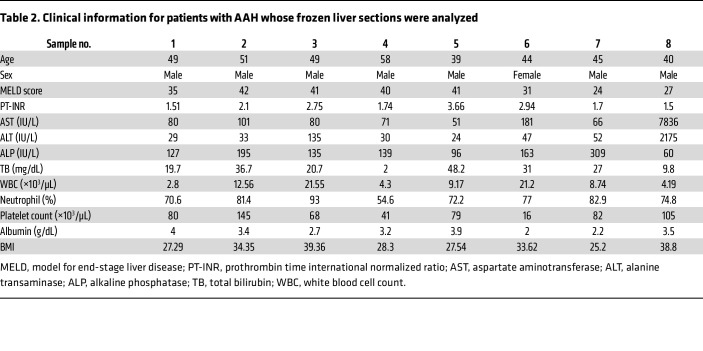
Clinical information for patients with AAH whose frozen liver sections were analyzed

**Table 3:**
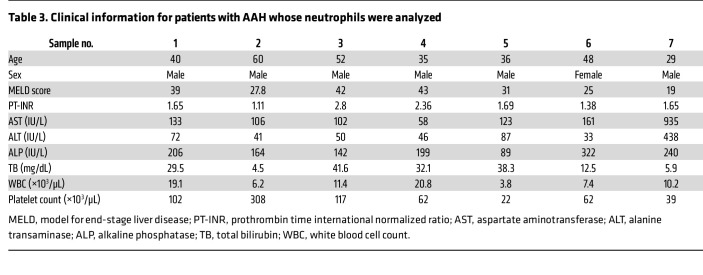
Clinical information for patients with AAH whose neutrophils were analyzed

## Version 1 06/25/2024

In-Press Preview

## Version 2 08/15/2024

Electronic publication
