# Supplementary material for: Neutrophils insert elastase into hepatocytes to regulate calcium signaling in alcohol-associated hepatitis
Source: J Clin Invest. 2024 Jun 25;134(16):e171691. doi: 10.1172/JCI171691 (PMC11324315; doi:10.1172/JCI171691)
Supplement: Supplemental data [file jci-134-171691-s042.pdf]

Supplemental Methods

Isolation of neutrophils from human blood

Peripheral venous blood samples were collected in K2 EDTA tubes (Becton, Dickinson and Company, Franklin Lakes, New Jersey, USA, 366643). After collecting the neutrophil layer using PolymorphPrep (Proteogenix, Schiltigheim, France, AN1114683), cells were washed with diluted HEPES to remove PolymorphPrep by centrifugation at 400 x g for 10 minutes. To remove erythrocytes, cells were incubated with ACK lysis buffer (Lonza, Bend, Oregon, USA, BP10-548E) for 5 minutes at 37°C. Neutrophils were harvested by centrifugation and resuspended in the medium to use or resuspended in RPMI 1640 medium supplemented with 10% heat-inactivated fetal bovine serum (Gibco, 10438026), 100 U/ml penicillin G and 100 μg/ml streptomycin (Gibco, 15140122).

Immunohistochemistry staining for human liver specimens

The primary antibody incubation was performed overnight at a ratio of 1:100 at 4 °C. Antigen retrieval was performed in a steamer for 20 minutes with citrate 10 mM, pH 6 (Sigma, #C999) and counterstained with Harris Hematoxylin (Sigma cat# GHS280). Images were acquired on an Olympus BX51 microscope with a 20X objective. Images were acquired from 4-5 different areas to quantify the number of neutrophil elastase- or MPO-positive cells in each image. High-magnification representative images of elastase- or MPO-positive granules in hepatocytes were taken at 60x.

Cell culture, co-culture with neutrophils, reagents

Human hepatocytes were cultured with Hepatocyte Basal Medium (Lonza, CC-3197) with dexamethasone and insulin (each 0.1 μM, Millipore-Sigma, D4902, and I2643 respectively) and overlaid with Matrigel (Corning, 356237). Primary mouse hepatocytes were cultured with William's E Medium (Gibco ™ #12551032) with supplemented with 10% heat-inactivated fetal bovine serum (Gibco, 10438026), 100 U/ml penicillin G and 100 μg/ml streptomycin (Gibco, 15140122). HepG2 cells were cultured with Eagle's Minimum Essential Medium

(EMEM, ATCC, 30-2003) supplemented with 10% heat-inactivated fetal bovine serum (Gibco, 10438026), 100 U/ml penicillin G and 100 µg/ml streptomycin (Gibco, 15140122), at 37°C, 5% CO_2. These cells were maintained in Dulbecco's MEM (DMEM, ATCC, 30-2002) with the same additives and conditions as for HepG2 cells. HepG2 cells were treated with neutrophil elastase (Millipore-Sigma, 324681), neutrophil MPO (Millipore-Sigma, 475911), or HMGB1 (R&D systems, 1690-HMB) at the indicated concentrations for 20 hours. MG132 (Millipore-Sigma, 474790) at 50 µM was used as the proteasome inhibitor, Bafilomycin A1 (Sigma Aldrich, 19-148) at 50 nM as the autophagy inhibitor, and N-acetyl-L-leucyl-L norleucinal (ALLN, Millipore-Sigma, 208719) at 50 µM as the calpain inhibitor, each administered to HepG2 cells for 1 hour as pretreatment. The cell-permeable caspase-3 inhibitor (Millipore-Sigma, 235423) was used at 50 µM, and the cell-permeable trypsin inhibitor, AdaAhx3L3VS (adamantane-acetyl-(6-aminohexanoyl)3-(leucinyl)3-vinyl-(methyl)-sulfone) (Millipore-Sigma, 114802) was used at 50 nM for 20 hours. The cell-permeable serine protease inhibitor, AEBSF (4-(2-aminoethyl) benzenesulfonyl fluoride hydrochloride, MP Biochemicals, 02193503-CF) was used at 10 µM. PF1355 (Cayman Chemicals, 22222) was used at 10 µM. N-Acetyl-L-cysteine (NAC) was used at 5mM.

Electron microscopy

HepG2 cells after co-culture with/without neutrophils for 1 hour on coverslips were fixed in 2.5% glutaraldehyde in 0.1M sodium cacodylate buffer pH7.4 for 1 hour, then rinsed in PBS. They were post-fixed in 0.5% osmium tetroxide and 0.8% potassium ferrocyanide in PBS for 1 hour. These were then rinsed in sodium cacodylate and distilled water and en-bloc stained in aqueous 2% uranyl acetate for 1 hour, followed by further rinsing in distilled water and dehydrated through an ethanol series to 100% ethanol. The cells were infiltrated with Embed 812 (Electron Microscopy Sciences) resin, gelatin capsules were filled with resin and inverted over the coverslips, and baked at 60 °C for 24 hours. After detaching the coverslip from the resin, the hardened blocks were cut using a Leica UltraCut UC7. 60nm sections were collected on formvar/carbon-coated grids and contrast stained using 2% uranyl acetate and lead citrate. The sections were viewed in a FEI Tecnai Biotwin TEM at 80Kv and Images were taken using AMT NanoSprint15 MK2 sCMOS camera. For immunoelectron

microscopy, the sample pellet was fixed in 4% PFA plus 0.125% glutaraldehyde in PBS for 15-30minutes at room temperature followed by further fixation in 4% PFA in PBS at 40C for 1 hour. They were rinsed in PBS and re-suspended in 10% gelatin, spun down and chilled blocks, then trimmed and placed in 2.3M sucrose overnight on a rotor at 4°C. They were transferred to aluminum pins and frozen rapidly in liquid nitrogen. The frozen blocks were cut on a Leica Cryo-EMUC6 UltraCut and 60nm thick sections were collected using Tokuyasu method (66) and placed on carbon/formvar coated grids and floated in a dish of PBS for immunolabeling. Immunolabeling of the ultra-thin sections were processed according to Slot and Geuze (67) and grids were incubated on a primary antibody rabbit anti-MPO (Fisher Scientific, RB373A0, 1:100 dilution) at dilution 1:200 for 1 hour rinsed and then on 10nm Protein A gold (Utrecht Medical Center) as a secondary antibody for 1 hour. All grids were rinsed in PBS, fixed using 1% glutaraldehyde for 5mins, rinsed again and transferred to a UA/methylcellulose drop for 10 minutes before being collected and dried. The 60nm sections on grids were viewed FEI Tecnai Biotwin TEM at 80Kv and Images were taken using AMT NanoSprint15 MK2 sCMOS camera. The number of MPO-bound gold nanoparticles entering HepG2 cells, co-cultured with or without neutrophils, was counted and compared by dividing by the area of HepG2 cells.

Immunoblotting analysis

All samples were lysed with RIPA buffer (Thermo Fisher Scientific, 89900) with a protease and phosphatase inhibitor cocktail (Thermo Fisher Scientific, 1861281). Bradford assay (#23200, Thermo Fisher) was used to determine protein concentrations, and loading samples were diluted with sample buffers (NP0007 & NP0004, Thermo Fisher), then boiled for 10 minutes at 70 °C according to the manufacturer's protocol. These samples were loaded onto NuPAGE™ Protein Gels. Proteins were transferred to the nitrocellulose membrane (Bio-Rad #1620145). Membranes were blocked with 5% milk (Omniblok™, AmericanBIO, AB10109) for 30 min at room temperature and reacted with primary antibody (1:1500) overnight at 4°C. Secondary antibodies, anti-rabbit (Invitrogen, #A 27036) or anti-mouse (Cytiva, NA931Cay) were used at 1:3000 for 1 hour at room temperature. Pierce™ ECL Western Blotting Substrate (#32106, Thermo Fisher) was used for detection. The following primary antibodies were used: ITPR1 (Alomone labs, ACC-019), ITPR2 (Santa Cruz Biotechnology,

398434), ITPR3 (BD Transduction Laboratories, 610313), SERCA2 (Cell signaling, 4388), Calnexin (Abcam, ab92573), SEC61B (Thermo Fisher Scientific, PA3-015), MPO (Fisher Scientific, RB373A0), elastase (R&D systems, MAB91671), Phospho-Erk1/2 (Cell Signaling, #9101), Erk1/2 (Cell Signaling, #9102) and GAPDH (Thermo Fisher Scientific, Clone 6C5, AM4300), Serpin A3 (NovusBiologicals, #NBP2-52559) , Cathepsin G (Thermo Fisher Scientific, # PA5-99402), MMP9 (R&D Systems, #AF911), Albumin (ProteinTech, #16475-1-AP), CyclinD1 (Cell Signaling, #2978S).

Confocal fluorescence imaging of hepatocytes

The following primary antibodies were used:Anti-MPO (Fisher Scientific, #RB373A0 or Invitrogen, MA1-34067), anti-neutrophil elastase (R&D systems, #MAB91671 or Abcam, #ab131260), anti-Lamp1 (Cell Signaling, # 15665). Secondary antibodies were incubated with goat anti-mouse Alexa 555 and goat anti-rabbit Alexa Fluor 488 secondary antibodies (1:500, Invitrogen, A28180 and A-11008, respectively) for 1 hour at room temperature. The cytoskeleton was stained with Phalloidin (1:500, Invitrogen, A22287) and nuclei with DAPI or Hoechst 33342 (1:1000, Invitrogen, D1306 and H3570). They were then mounted on antifade fluorescent mounting media (Electron Microscopy Sciences, 17966).

Calcium signaling

Fluorescence intensity of calcium-sensitive fluorescent dye, Fluo4/AM (Invitrogen, F14201) in HepG2 cells responding to ATP (Millipore-Sigma, 20-306) was monitored with a Zeiss LSM 710 confocal microscope. Briefly, cells on glass coverslips in HEPES buffer (pH 7.4) were loaded with the Fluo-4 (6 µM) for 30 minutes at 37°C. Then cells on coverslips were transferred to a custom-made perfusion chamber and perfused with HEPES buffer while stimulated with 20 µM ATP. Relative Fluo-4 fluorescence intensity after stimulation was analyzed using Image J (National Institutes of Health) in selected regions of interest and compared to the baseline. Quantification of calcium signals was compared by calculating the area under the curve after stimulation with ATP and 30 seconds.

Bulk RNA-seq and Ingenuity Pathway Analysis

cDNA and library preparation and sequencing: RNA was isolated using RNeasy Mini Kits (QIAGEN, 74104) from HepG2 cells co-cultured for 20 hours with or without neutrophils, or with or without the neutrophil granule fraction (each in triplicate). RNA integrity number was determined using an Agilent Bioanalyzer 2100 (Agilent Technologies, Santa Clara, California, USA). On average, 40 million sequencing reads (100 bp paired end) of each sample were obtained using an Illumina 6000 Sequencer. *Ingenuity Pathway Analysis:* The sequencing data of HepG2 co-cultured with or without neutrophils were analyzed to get the candidates for functional blocking experiments. Quality control was performed by FastQC (Galaxy V.0.2.6+galaxy0) and Trimmomatic (Galaxy V.0.2.6+galaxy0). To remove Illumina adaptor sequences, the TruSeq3-paired-end file was used in Trimmomatic. The reads were mapped to the University of California Santa Cruz hg19 human genome by STAR (Galaxy V.2.6.0b-1). The raw read counts of genes were counted by feature Counts (Galaxy V.1.6.4+galaxy1) and the differential expression analysis was conducted by DESeq2 (Galaxy V.2.11.40.6). A list of gene transcripts satisfying FDR<0.05, fold change ±2, was input to IPA (Ingenuity Pathway Analysis, QIAGEN,). Integrins αM and 2 were selected as candidates from the top5 classical pathways obtained from IPA, from the list of genes defined as present in the plasma membrane in the gene ontology term. Bulk RNA-seq data analysis: To capture expression of genes commonly changed in HepG2 cells treated with neutrophil or granule fractions, both of sequencing data were analyzed. Low quality ends (less than phred score=30) and short read length (minimum length=30) was trimmed using PRINSEQ++ (version1.2). Trimmed reads were aligned to the hg38 genome reference using STAR (v2.7.1) and subsequently, RSEM (RNA-Seq by Expectation-Maximization) was used to count reads mapping to the genes from Ensembl release 93. Normalized gene counts were evaluated using the R package DESeq2. Heatmaps showed row-normalized relative gene expression z-scores across columns.

Mouse model of AAH

The chronic + binge model was conducted using 9- to 10-week-old male C57BL/6N mice (Charles River Laboratories), with four control and four AAH.

The ELF model was performed in four experiments. First, 9- to 10-week-old male C57BL/6N mice (Charles River Laboratories) were used in a control group of 4 and an AAH group of 4. Second, to examine phenotypic differences due to neutrophil elastase deficiency in the AAH mouse model, heterozygous (The Jackson Laboratory, B6.129X1-*Elane*^{tm1Sds}/J) crosses were used to generate WT mice (Elane+/+) and homozygous deficient mice (Elane-/-). Due to the small number of male same-week-olds available at one time, bone marrow from each mouse was transplanted into male C57BL/6NJ mice (The Jackson Laboratory) before ELF model experiments were performed. For bone marrow transplantation, donors were two 4-week-old male WT mice (Elane+/+) and two homozygous deficient mice (Elane-/-), each generated from heterozygous crossbreeding. Bone marrow was harvested, and single cells were adjusted in PBS to a final concentration of 5 x10^6/100ul/mouse after passing through a cell strainer. Recipients were male C57BL/6NJ mice (11 weeks old) as recommended by The Jackson Laboratory. 32 mice were gamma irradiated with Cs at 5 Gy twice on the day of transplantation. Then bone marrow prepared as described above was injected transvenously. ELF model experiments were started 4 weeks after transplantation. Elane-/- mice were 8 in the AAH group and 8 in the control group, and wild-type mice were 8 in the AAH group and 8 in the control group.

Third, to test the effect of elastase inhibitors on the AAH mouse model, male C57BL/6N mice (Charles River Laboratories) aged 9-11 weeks were used in the following groups: control group 6 mice, control + Sivelestat group 7 mice, control + AEBSF group 7 mice, AAH group 8 mice, AAH + Sivelestat group 8 mice, AAH +ABESF group was 8 mice. Experiments with these inhibitors followed the previously published protocol (69, 70).

Fourth, to investigate the role of ITPR2 in hepatocyte proliferation in the AAH model, we performed experiments using male systemic ITPR2-/- mice. Male systemic ITPR2-/- mice were 5 in the AAH group and 6 in the control group, and WT mice were 7 in the AAH group and 6 in the control group. Since ITPR2-/- mice have been crossed with pure C57BL/6N wild-type (WT) mice (Charles River Laboratories) for eight generations, C57BL/6N wild-type (Charles River Laboratories) mice were purchased to serve as wild-type mice.

For the chronic + binge model, Lieber-DeCarli control and ethanol liquid diets were obtained from BioServ (#1259SP and #1258SP, Flemington, New Jersey, USA). After acclimatization to tube feeding with the control diet ad libitum for 5 days, mice received either the control or the ethanol diet for 10 days. On day 11, mice were gavaged with 5 g ethanol/kg body weight and sacrificed 9 hours later. The ELF model is almost identical to the NIAAA model, but in the ethanol diet group, in addition to the ethanol liquid diet plus 30% fructose (Sigma-Aldrich), on the 4th day, 7th day, and 10th day of the ethanol liquid diet regimen, LPS (Escherichia coli O111:B4, 0.05 mg/kg body weight, Sigma-Aldrich) was injected intraperitoneally into mice. Therefore, the control group was fed the same amount of food consumed by ethanol-fed mice and with a non-isocaloric control diet.

Cell proliferation assay

Briefly, HepG2 cells were plated on coverslips and co-cultured with or without neutrophils for 12 hours. Then, after 2 hours of replacing half the medium with 5-ethynyl-2'-deoxyuridine (EdU) solution, the cells were fixed with 4% PFA (Electron Microscopy Science, 15710), and Edu-incorporated cells were detected by fluorescence.

Proteome Analysis and Mass Spectrometry

Non-treated HepG2 cell lysates (sonicated on ice) or HepG2 cell lysates incubated with neutrophil elastase at a final concentration of 1ug/ml for 5 minutes were sent to Applied Biomics, Inc. on dry ice for proteomic analysis. Thereafter, 2-D cell lysis buffer (30 mm Tris/HCl, pH 8.8, containing 7 M urea, 2 M thiourea, 4% CHAPS) was added and the supernatant was collected after centrifugation at 25,000g for 30 min at 4°C.The lysate was diluted with 2D cell lysis buffer to a final protein concentration of 6 mg/mL. Samples were labeled with Cy3 and Cy5 dyes, respectively, and equal amounts (30 μg) of protein were loaded into strip holders. IEF was performed in the dark at 20°C according to GE BioSciences protocol. after completion of the IEF run, IPG strips were incubated in newly prepared equilibration buffer #1 (50 mm Tris/HCl, pH 8.8, containing 6 M urea, 30% glycerol, 2% SDS, trace amounts of bromophenol blue, and 10 mg/mL DTT) for 15 minutes with gentle

shaking. The strips were then rinsed in newly prepared equilibration buffer #2 (50 mm Tris/HCl, pH 8.8, containing 6 M urea, 30% glycerol, 2% SDS, trace amounts of bromophenol blue and 45 mg/mL iodoacetamide) for 10 minutes with gentle shaking. IPG strips were then washed once with SDS-gel running buffer, transferred to SDS/PAGE (12% SDS-gels prepared using low fluorescence glass plates), and sealed with 0.5% (w/v) agarose solution (in SDS-gel running buffer) .

SDS/PAGE was performed at 15°C until the dye was removed from the gel. Image scans were performed immediately after SDS/PAGE using a Typhoon TRIO (GE Healthcare) according to the manufacturer's protocol. Scanned images were analyzed with Image QuantTL software (GE Healthcare), and in-gel and cross-gel analyses were performed using DeCyder software version 6.5 (GE Healthcare). Ratio changes in protein expression differences were obtained from in-gel DeCyder software analysis. Based on the in-gel analysis and spot picking design by DeCyder software, the spots of interest were picked using an Ettan Spot Picker (GE Healthcare). Gel spots were washed several times and digested in-gel with modified porcine trypsin protease (Trypsin Gold, Promega). Digested trypsin peptides were desalted with Zip-tip C18 (Millipore); 0.5 μL of matrix solution (α-cyano-4-hydroxycinnamic acid, 5 mg/mL, 50% acetonitrile, 0.1% trifluoroacetic acid, 25 mm ammonium bicarbonate) from Zip-tip Peptides were eluted and spotted onto MALDI plates. MALDI-TOF (MS) and TOF/TOF (tandem MS/MS) were performed on a 5800 mass spectrometer (AB Sciex). MALDI-TOF mass spectra were acquired in reflectron positive ion mode, averaging 2000 laser shots per spectrum.TOF/TOF tandem MS fragmentation spectra were obtained for the 5-10 most abundant ions present in each sample (trypsin autolysis An average of 2000 laser shots per fragmentation spectrum was acquired for each of the peptides and other known background ions. Both the obtained peptide masses and associated fragmentation spectra were sent to GPS Explorer version 3.5 with the MASCOT search engine (from Matrix Science) for analysis by the National Center for Biotechnology Information nonredundant (NCBInr) or the Swiss Protein database. Searches were performed with search parameters that did not constrain the molecular weight or isoelectric point of the protein, varied the carbamidomethylation of cysteines and oxidation of methionine residues, and allowed for one missed cleavage. Candidates with either a protein score CI% or ion CI% > 95 were considered significant.

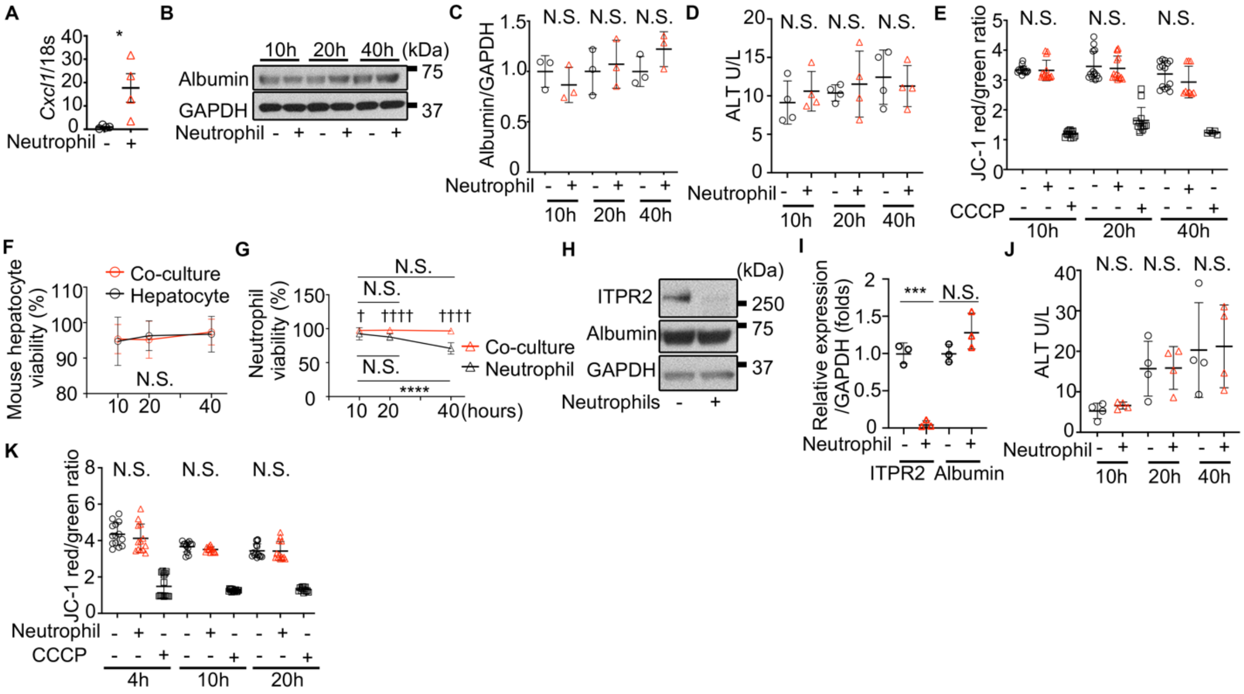

Supplementary Figure 1. Neutrophils decrease ITPR2 without impairing hepatocyte function. Studies were performed with HepG2 cells and human neutrophils (**A**-**E**) or with mouse primary hepatocytes and mouse neutrophils (**F**-**K**). (**A**) RT-qPCR shows that *Cxcl1* mRNA levels in HepG2 cells are increased after 20 hours of co-culture with neutrophils. (**B**-**D**) Co-culture with human neutrophils does not impair albumin synthesis or result in release of ALT from HepG2 cells. (**B**) Representative immunoblots and (**C**) quantitation of the blots show that albumin levels are not changed in HepG2 cells after co-culture with neutrophils for 10-40 hours. (**D**) ALT levels in the culture medium supernatant of HepG2 cells are not changed after co-culture with neutrophils for 10-40 hours. (**E**) Co-culture with neutrophils for indicated times does not alter mitochondrial membrane potential of HepG2 cells with JC-1 staining assay; CCCP (100 uM) treatment to dissipate membrane potential was used as negative control. (**F**) Co-culture with neutrophils does not decrease viability of mouse hepatocytes. Double staining (calcein-AM for alive (green), ethidium homodimer-1 for dead (red)) was performed in co-culture experiments of mouse neutrophils and mouse primary hepatocytes for the specified time. (**G**) Viability of neutrophils decreases over time unless they are co-cultured. Differences between monoculture and co-culture are indicated by daggers and changes over time are indicated by asterisks. Measured 5-6 fields per coverslip. (**H-J**) Co-culture with mouse neutrophils does not impair albumin synthesis or result in release of ALT from mouse hepatocytes. (**H**) Representative immunoblots and (**I**) blot quantification show that ITPR2 is dramatically reduced in HepG2 cells co-cultured with neutrophils for 1 hour, while albumin levels are unchanged. (**J**) ALT levels in the culture medium supernatant of mouse hepatocytes are not changed after co-culture with neutrophils for 10-40 hours. (**K**) Co-culture with mouse neutrophils for indicated times does not alter mitochondrial membrane potential of mouse hepatocytes. (**A**) is shown as mean±SEM, all others as mean±SD. Three or more independent experiments were performed, and all statistical analyses were performed using unpaired t-tests. (N.S., not significant, *or †$p<0.05$, ***$p<0.001$, ****or ††††$p<0.0001$, unpaired t-test).

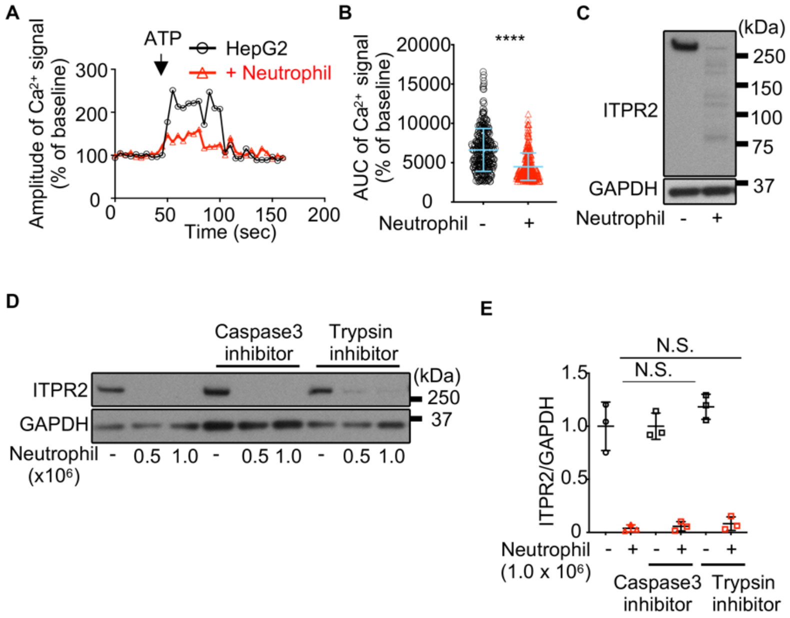

Supplementary Figure 2. Neutrophil-induced loss of ITPR2 and calcium signals in HepG2 cells occurs rapidly and is not mediated by caspse-3 or trypsin. (**A**) Calcium signals in HepG2 cells are altered by neutrophils. Representative tracings of Fluo 4 fluorescence intensity of HepG2 cells alone or co-cultured with neutrophils for 1 hour. Cells were stimulated with 20 μM ATP. (**B**) Calcium signaling in HepG2 cells as measured by the area under the curve upon ATP stimulation is diminished by neutrophils (6 coverslips of HepG2, n=484; 6 coverslips of HepG2+neutrophils, n=420, from 2 independent experiments, graphs are mean ± SD, ****p <0.0001, unpaired t-test). (**C**) Representative immunoblots showed that ITPR2 bands were detected in a ladder-like pattern in HepG2 cells co-cultured with neutrophils for 20 hours by film developed with a 30-minute exposure. (**D**) Representative immunoblots shows that loss of ITPR2 in HepG2 cells is not blocked by treatment with caspase-3 inhibitor (50 μM) or trypsin inhibitor (AdaAhx3L3VS, 50 nM) by co-culture with 0.5 or 1 million neutrophils. for 20 hours. (**E**) Quantitative analysis of HepG2 cells co-cultured with 1 million neutrophils is shown. Data are mean±SD, n=3 (N.S., not significantly different by one-wayANOVA).

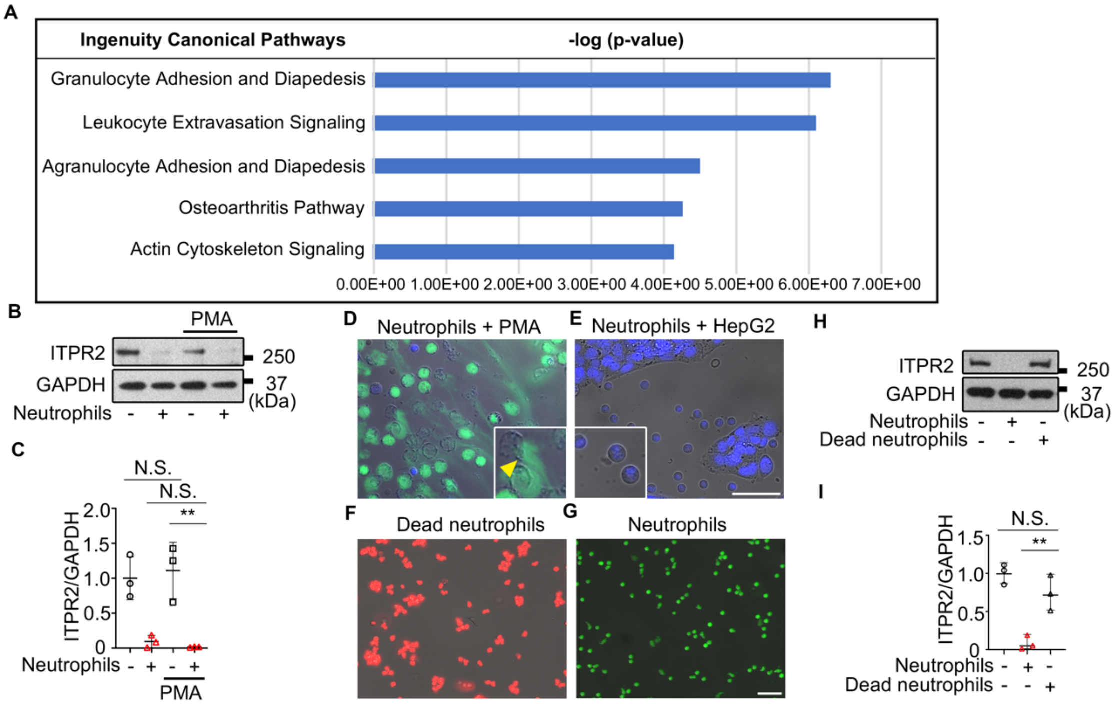

Supplementary Figure 3. Neutrophils activate specific signaling pathways in HepG2 cells, which is not replicated by dead neutrophils. (A) The top 5 canonical pathways derived from ingenuity pathway analysis are shown. A list of gene transcripts satisfying false discovery rate (FDR)<0.05, fold change ±2, from RNA-seq data of HepG2 cells co-cultured with neutrophils for 20 hours, was analyzed. The values of -log(p-value) for each pathway are 6.294 (granulocyte adhesion and diapedesis), 6.103 (leukocyte extravasation signaling), 4.488 (agranulocyte adhesion and diapedesis), 4.262 (osteoarthritis pathway), 4.146 (actin cytoskeleton signaling) respectively. (**B**) Representative immunoblots and (**C**) quantitation showed that ITPR2 levels in HepG2 cells were reduced after 4 hours of co-culture with neutrophils with or without phorbol myristate acetate (PMA, 200 nM). (**D**, **E**) Representative phase contrast images of neutrophils incubated with PMA for 4 hours (**C**) and neutrophils after co-culture with HepG2 cells for 4 hours (**D**). Labels are SYTOX green and nuclear stain Hoechst 33342 (blue). Scale bar, 50 µm. Inset is a 2x enlargement showing that yellow arrowheads indicate extacellular nucleic acids of neutrophils stained in (**D**) but not (**E**). (**F**, **G**) Representative phase contrast images of neutrophils using double staining (calcein-AM for alive (green), ethidium homodimer-1 for dead (red)). Neutrophils treated with 100% methanol for 5 min (**F**), untreated neutrophils (**G**). Scale bar, 50 µm. (**H**) Representative immunoblots and (**I**) quantitation show that ITPR2 levels in HepG2 cells are not decreased after co-culture with dead neutrophils for 1 hour instead of intact neutrophils. All data are presented as mean±SD, n=3. All statistical analyses were performed using one-way ANOVA (N.S., not significant, **p<0.01).

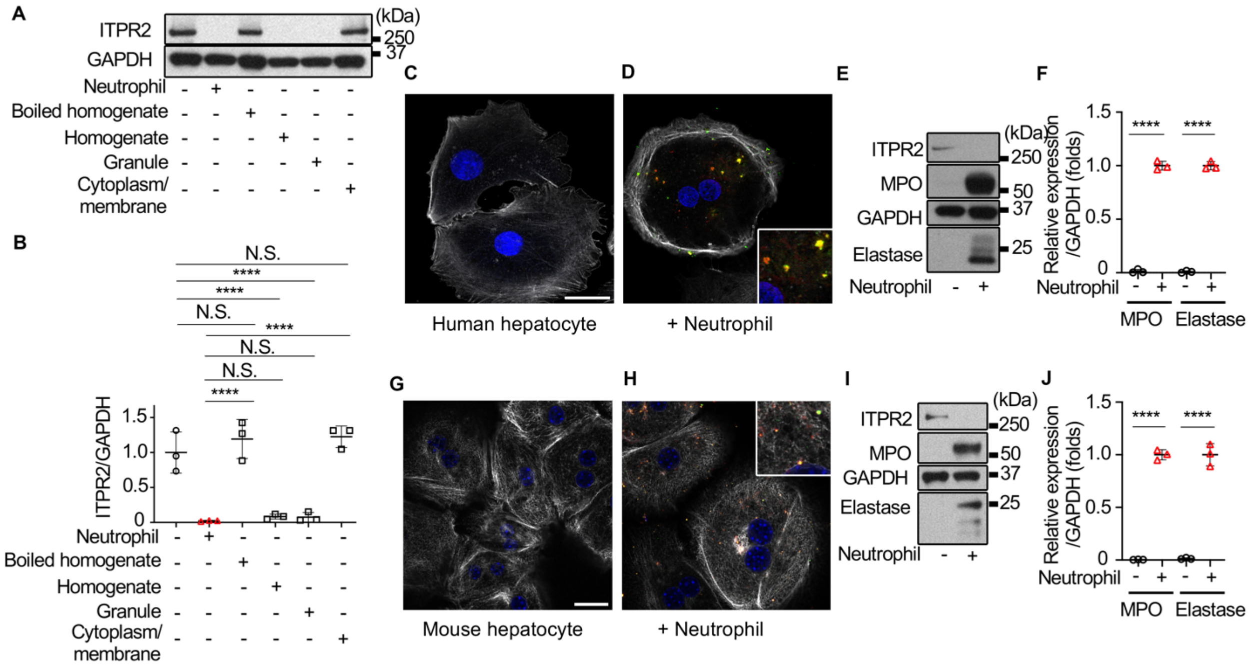

Supplementary Figure 4. Neutrophil granule contents translocate to both human (A-F) and mouse (G-J) hepatocytes. (**A**) Representative immunoblots and (**B**) quantitation show that ITPR2 levels in human hepatocytes are decreased after co-culture with neutrophils or homogenate or granule fractions for 1 hour but not boiled homogenate nor cytoplasm/membrane fractions. (**C**, **D**) Representative immunostaining images of human hepatocytes only (**C**) and co-culture with neutrophils for 1 hour and then washing to remove neutrophils (**D**) with anti-myeloperoxidase (MPO, green), anti-elastase (red) antibodies, nuclear staining (DAPI, blue), and Phalloidin (gray). Scale bar, 20 µm. Inset is a 2x enlargement showing partial co-localization of MPO and elastase (yellow).(**E**) Representative immunoblots and (**F**) quantitation show that ITPR2 levels in human hepatocytes are decreased after co-culture with neutrophils for 1 hour while MPO and elastase levels are increased. (**G**, **H**) Representative immunostaining images of mouse hepatocytes only (**G**) and co-culture with neutrophils for 1 hour and then washing to remove neutrophils (**H**) with anti-myeloperoxidase (MPO, green), anti-elastase (red) antibodies, nuclear staining (DAPI, blue), and Phalloidin (gray). Scale bar, 20 µm. Inset is a 2x enlargement showing partial co-localization of MPO and elastase (yellow)(**I**) Representative immunoblots and (**J**) quantitation show that ITPR2 levels in mouse hepatocytes are decreased after co-culture with neutrophils for 1 hour while MPO and elastase levels are increased. All data are presented as mean±SD, n=3. Statistical analysis in (**B**) was one-way ANOVA and others were unpaired t-test. (N.S., not significant, ****$p<0.0001$)

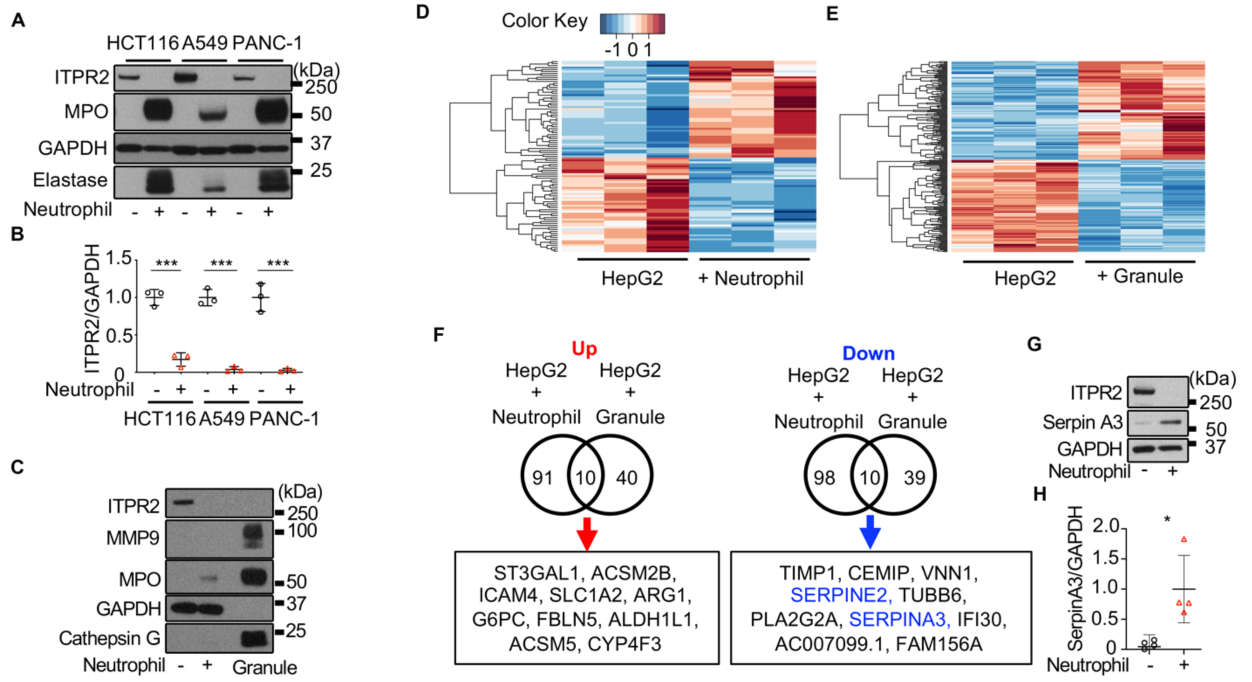

Supplementary Figure 5. Hepatocytes respond to neutrophil elastase by altering their expression of serpin E2 and serpin A3. (**A**) Transfer of MPO and elastase from neutrophils and loss of ITPR2 occurs in multiple cell types. Representative immunoblots (**A**) evaluating ITPR2, MPO, and neutrophil elastase in HCT116 (colon) or A549 (lung) or PANC-1 (pancreas) after 1 hour co-culture with neutrophils. Quantitation for ITPR2 (**B**) are presented as mean±SD, n=3. Statistical analysis was performed using unpaired t-test (***$p<0.001$). (**C**) Representative immunoblot evaluated for MMP9 and Cathepsin G in HepG2 cells co-cultured with neutrophils for 1 hour. ITPR2 in HepG2 cells is degraded and MPO is transferred, but not MMP9 or Cathepsin G. The right lane is a blot of neutrophil granule fraction as a positive control. (**D**-**F**) RNA-seq of HepG2 cells co-cultured for 20 hours with neutrophils or extracted granule fractions (both from 3 different volunteers) and respective controls (HepG2) was performed. (**D**) Heat maps of HepG2 cells co-cultured with neutrophils and HepG2 cells cultured alone. (**E**) Heat maps of HepG2 cells incubated with granule fraction and HepG2 cells alone. (**F**) Venn diagram shows the number of genes whose expression was significantly increased (UP) or decreased (Down) for HepG2 cells treated with neutrophils or their granule fraction compared to respective controls (false discovery rate (FDR) < 0.05, log fold change < -0.5 or > 0.5). A list of 10 genes commonly up- or down-regulated in the two RNA-seqs is shown and includes *Serpin E2* and *Serpin A3*. (**G**) Representative immunoblots and (**H**) quantitation show that Serpin A3 protein levels in human hepatocytes are increased after co-culture with neutrophils for 1 hour. Data are presented as mean±SD, n=4. Statistical analysis was performed using unpaired t-test. (*$p<0.05$)

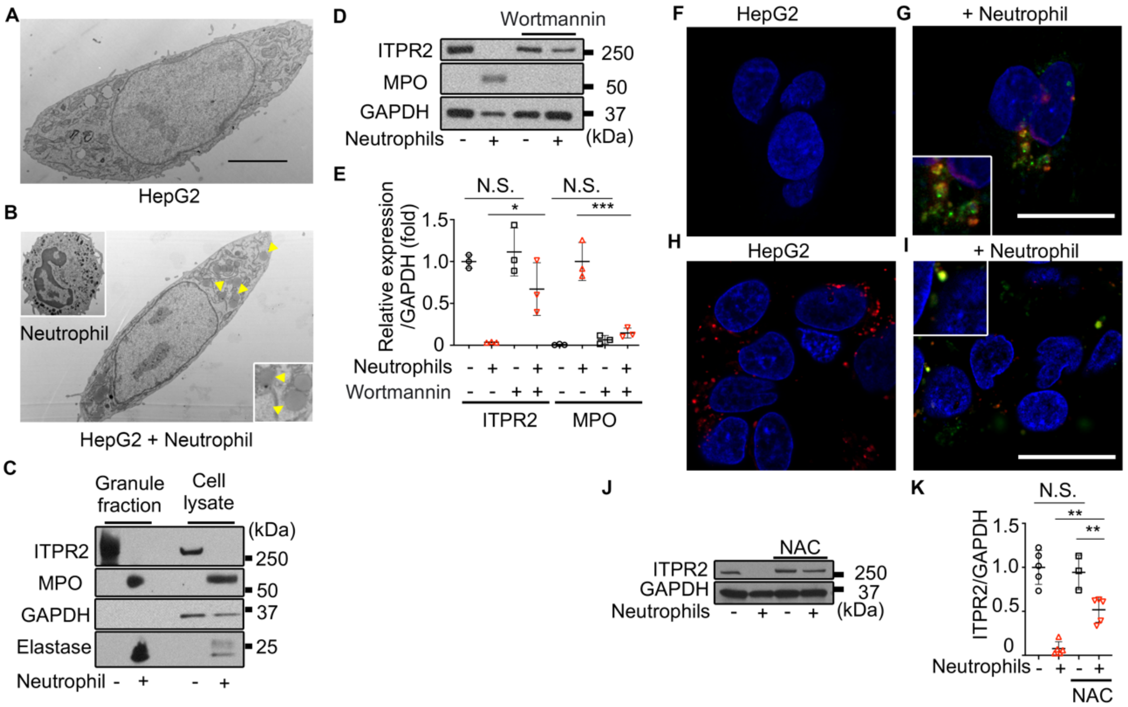

Supplementary Figure 6. Transfer and trafficking of neutrophil granule contents into HepG2 cells. (**A**, **B**) Representative transmission electron micrograph of HepG2 cells co-cultured with neutrophils for 1 hour (**B**) or HepG2 cells alone (**A**). Scale bar, 5 µm. In (**B**), the top left shows a neutrophil at the same magnification, and the bottom right image shows a portion of a HepG2 cell magnified 2-fold, with yellow arrowheads indicating endosome-like structures. (**C**) Representative immunoblots show that the granule fraction isolated by the technique in Figure 4A from HepG2 cells co-cultured with neutrophils for 1 hour (second lane from Granule fraction) contains MPO and elastase, as does its total lysate (second lane from Cell lysates). (**D**) Representative immunoblots and (**E**) quantitation showed that the loss of ITPR2 and the appearance of MPO in HepG2 cells after co-culture with neutrophils for 1 h was prevented by pretreatment with the PI3K inhibitor wortmannin (20 µM). (**F**, **G**) Representative immunostaining images show that neutrophil granule fractions stained with PKH67 (green) are incorporated into HepG2 cells by incubation for 1 hour, and some of them are also stained with anti-elastase antibody (red). Nuclei are stained with DAPI (blue). Untreated HepG2 cells (**F**) and HepG2 cells after granule fraction administration (**G**). Inset is a 2x enlargement showing partial co-localization of PKH67 and elastase (yellow). Scale bar, 20 µm. (**H**, **I**) Representative confocal microscopy images show that neutrophil granule fractions stained with PKH67 (green) are incorporated into HepG2 cells by incubation for 1 hour, and some of them are also stained with Lysotracker red. Nuclei are stained with Hoechst 33342 (blue). Untreated HepG2 cells (**H**) and HepG2 cells after granule fraction administration (**I**). Inset is a 2x enlargement showing partial co-localization of PKH67 and Lysotracker (yellow). Scale bar, 20 µm. (**J**) Representative immunoblots and (**K**) quantitation show that that loss of ITPR2 in HepG2 cells after 20 hour co-culture with neutrophils is blocked by n-acetylcysteine (NAC, 5 mM). Data are presented as mean±SD, n=3-5. Statistical analysis was performed by one-way ANOVA. (N.S., not significant, *p<0.05, **p<0.01, ***p<0.001).

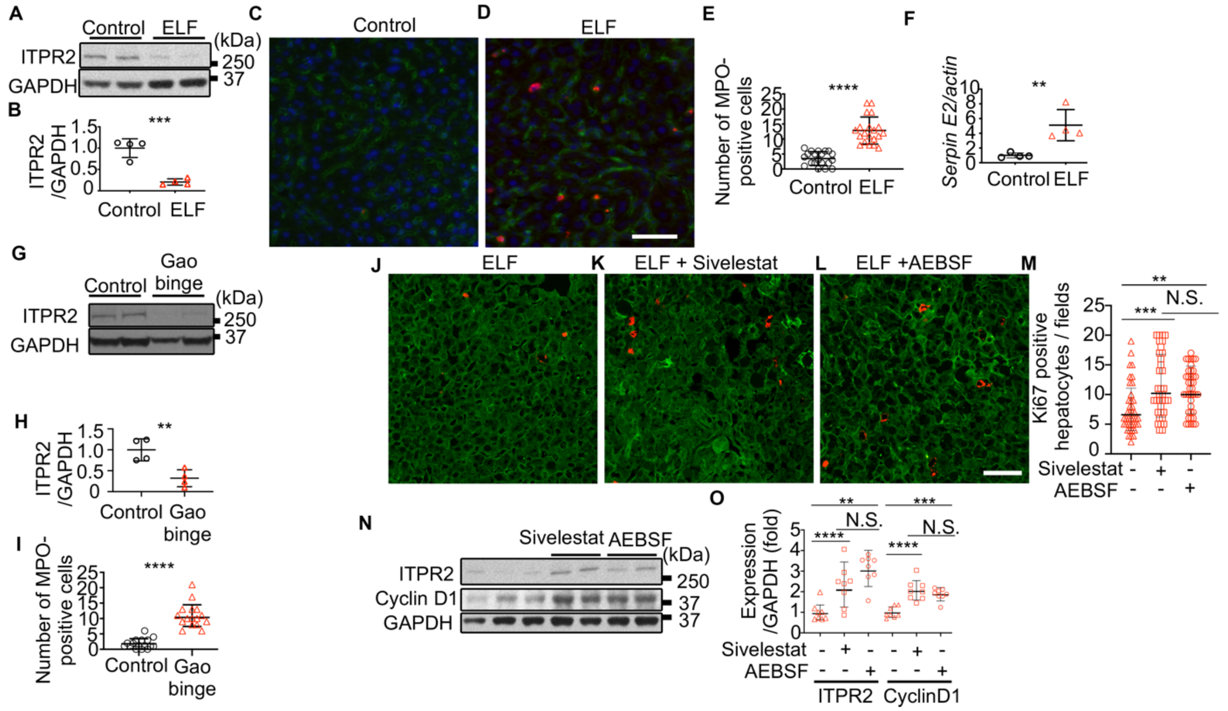

Supplementary Figure 7. A mouse model of alcohol-associated hepatitis (AAH) recapitulates neutrophil elastase-dependent loss of ITPR2 and impaired proliferation in hepatocytes. (**A-F**) AAH model mice on ethanol + LPS + fructose (ELF model;(41)) have increased numbers of neutrophils infiltrating the liver and decreased ITPR2 levels in liver homogenates. (**A**) Representative immunoblots and (**B**) quantitation of liver homogenates shows that ITPR2 levels are decreased in ELF. (**C**) Representative confocal images of livers from control and (**D**) ELF mice stained with anti-MPO (red) and CD31 (green) antibodies. (**E**) Quantitation shows that MPO-positive neutrophils are increased in the liver of ELF mice compared to control mice. Five fields for each mouse. (**F**)RT-qPCR shows that *Serpin E2* mRNA levels in liver of ELF are increased than control mice. (**G-I**) AAH model mice on chronic plus binge model (Gao binge; (52)) have increased numbers of neutrophils infiltrating the liver and decreased ITPR2 levels in liver homogenates. (**G**) Representative immunoblots and (**H**) quantitation of liver homogenates shows that ITPR2 levels are decreased in Gao binge. (I) Quantitation shows that MPO-positive neutrophils are increased in the liver of Gao binge compared to control mice. Four fields per mouse. (**J-M**) Proliferation marker Ki67 in hepatocytes are increased by elastase inhibitors (Sivelestat or AEBSF) of ELF mouse models. Representative confocal images of (**J**) ELF, (**K**) ELF + Sivelestat, and (**L**) ELF + AEBSF livers immunostained with anti-CK18 (green) and anti-Ki67 (red) antibodies. (**M**) Quantitation shows that the number of Ki67-positive hepatocytes was higher in ELF + Sivelestat and ELF + AEBSF than in ELF mice. 4-5 fields per mouse. (**N**, **O**) In ELF models, reduced ITPR2 levels are ameliorated by elastase inhibitors (Sivelestat or AEBSF) and hepatocyte proliferation is increased. (**N**) Representative immunoblots and (**O**) quantitation of ITPR2 and Cyclin D1 levels in liver homogenates shows that both are increased in ELF + Sivelestat and ELF + AEBSF mice. In all images, scale bars are 50 μm. Data are presented as mean±SD. (**M**) and (**O**) were analyzed by one-way ANOVA with n=8, all others by unpaired t-test with n=4. (N.S., not significant, **p<0.01, ***p<0.001, ****p<0.0001).

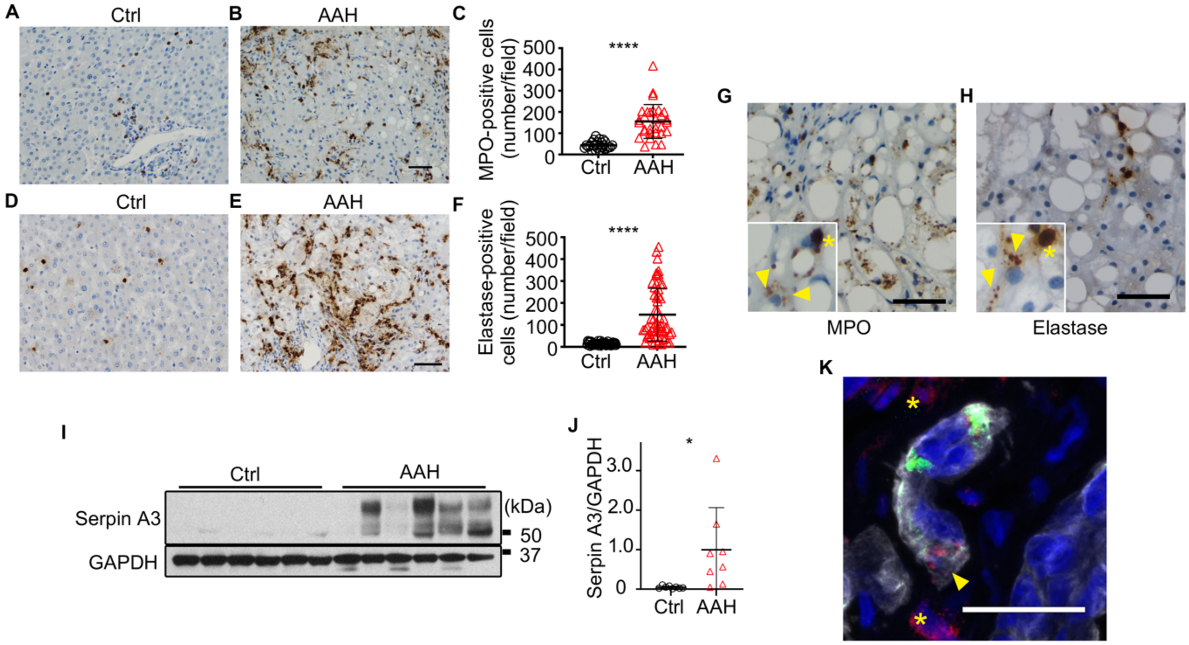

Supplementary Figure 8. MPO and elastase are transferred to hepatocytes and Serpin A3 is increased in patients with alcohol-associated hepatitis (AAH). (**A**-**F**) Liver images of AAH patients show infiltration of many neutrophils. Representative images of immunohistochemical staining with anti-MPO antibody in (**A**) histologically normal controls (Ctrl) and (**B**) liver biopsy specimens from AAH patients. Scale bar, 50 µm. (**C**) Quantitative measurement of MPO-positive cells confirmed the increased number of neutrophils in the liver of AAH. Three fields were quantified in each biopsy specimen (8 controls and 10 AAH patients). Representative images of immunohistochemical staining with anti-elastase antibodies in liver biopsy specimens from (**D**) histologically normal controls and (**E**) AAH patients. Scale bar, 50 µm. (**F**) Quantitative measurement of elastase-positive cells confirmed the increased number of neutrophils in the liver of AAH. 4-7 fields were quantified in each biopsy specimen (8 controls and 9 AAH patients). Data are shown as mean±SD, ****$p<0.0001$ by unpaired t-test. (**G**, **H**) Representative images of immunohistochemical staining for MPO (**G**) or elastase (**H**) in liver biopsies of AAH patients are shown. Scale bar, 50 µm. Inset is a 3x enlargement showing; yellow asterisks are neutrophils and yellow arrowheads are MPO- or elastase-positive granules inserted into hepatocytes. (**I**) Representative immunoblotting and (**J**) Quantitation of serpin A3 in liver homogenates showed that serpin A3 in the liver of AAH patients is increased. n=8, data are shown as mean±SD, *$p<0.05$ by unpaired t-test. (**K**) Representative images of frozen liver tissue from AAH patients stained with anti-cleaved caspase 3 antibody (green) and anti-elastase antibody (red), anti-CK18 antibody (gray) and DAPI (blue). The yellow asterisks are elastase-positive neutrophils, and the yellow arrowheads are neutrophils that appear to be embedded in cleaved caspase3-positive hepatocytes (CK18-positive, cytoskeleton). Scale bar, 50 µm.
