# Supplementary material for: Neutrophils insert elastase into hepatocytes to regulate calcium signaling in alcohol-associated hepatitis
Source: J Clin Invest. 2024 Jun 25;134(16):e171691. doi: 10.1172/JCI171691 (PMC11324315; doi:10.1172/JCI171691)

Full unedited blot for Figure 1K

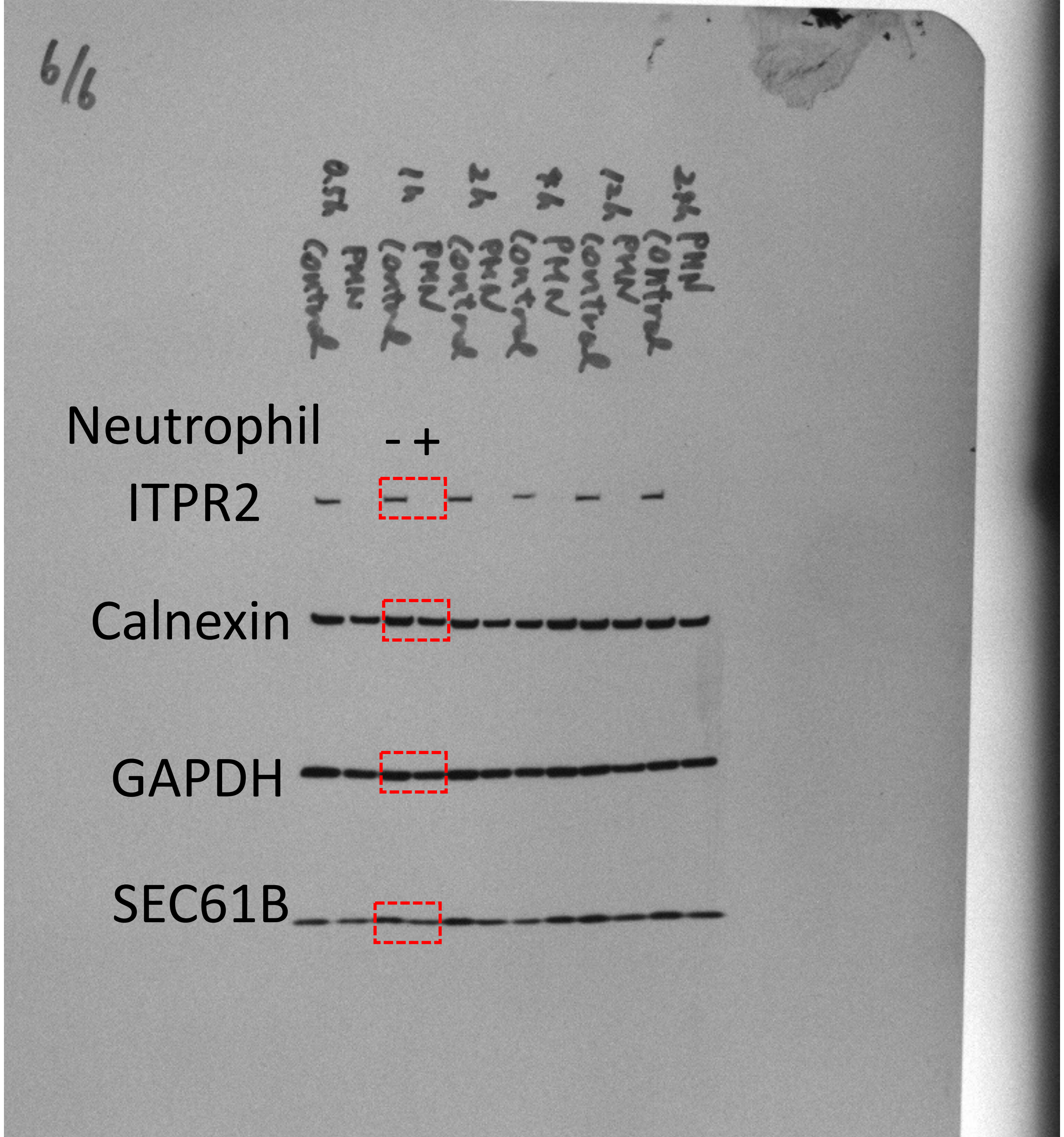

Full unedited blot for Figure 1K

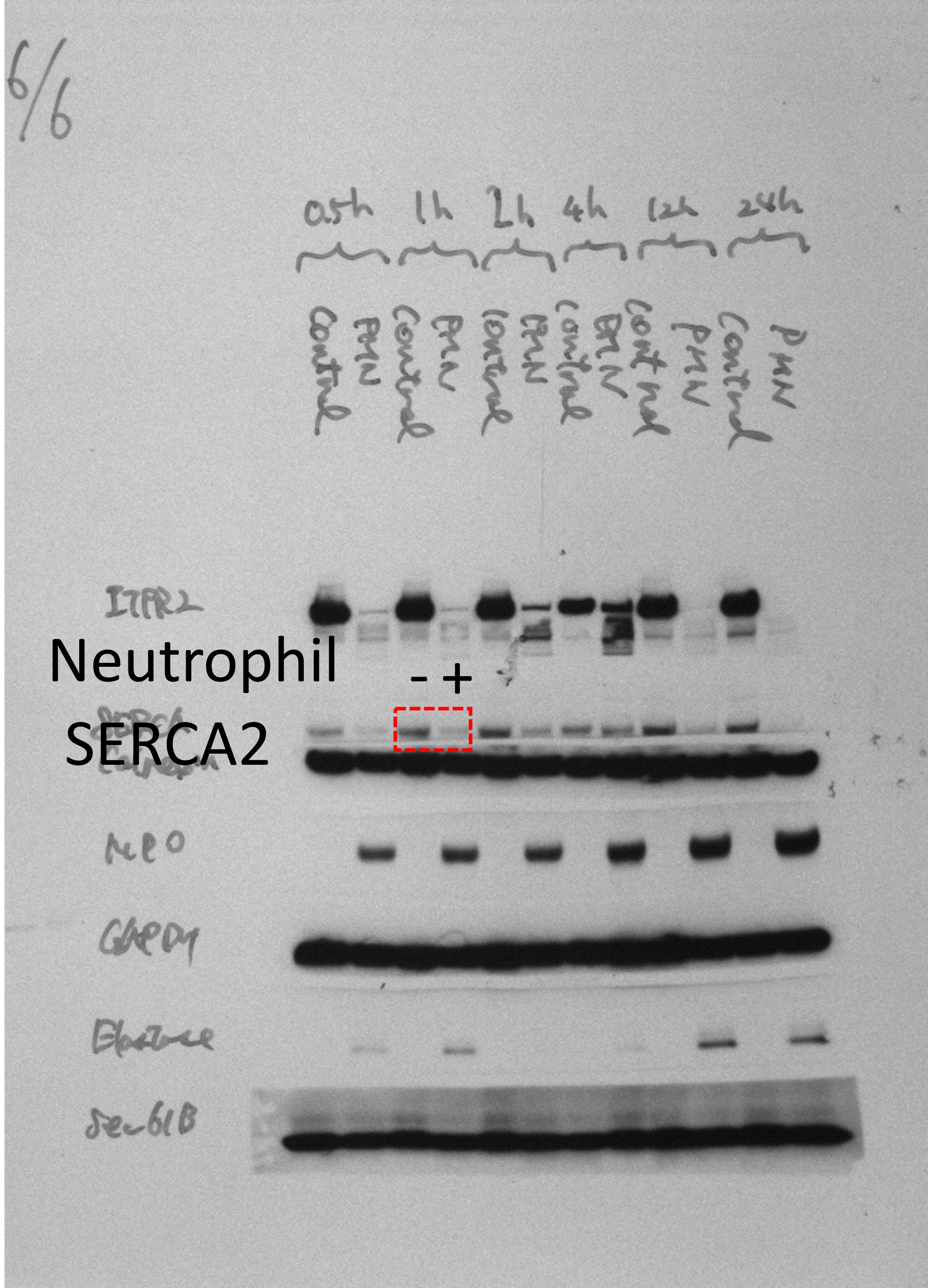

Full unedited blot for Figure 1K

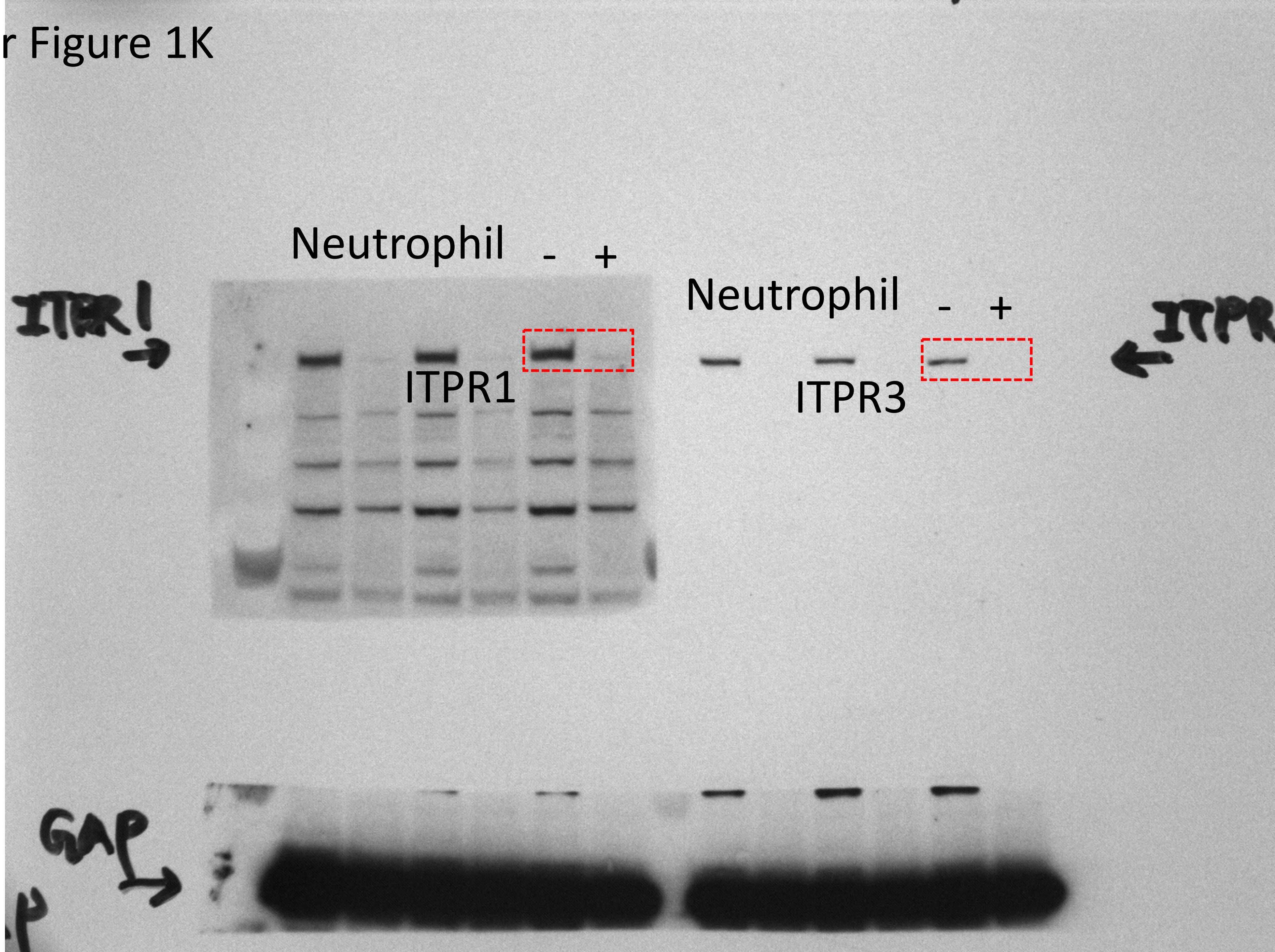

Full unedited blot for Figure 1M

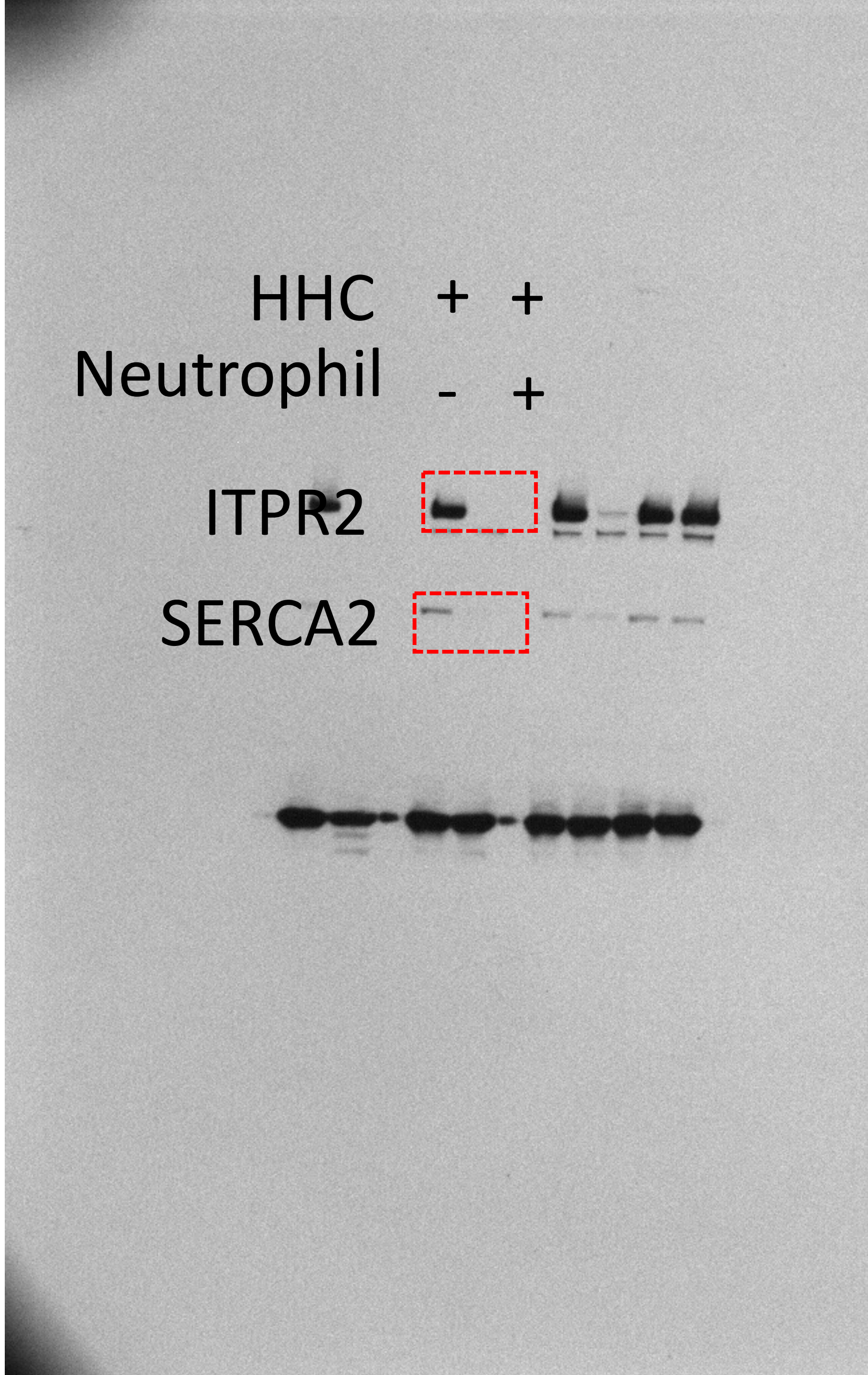

Full unedited blot for Figure 1M

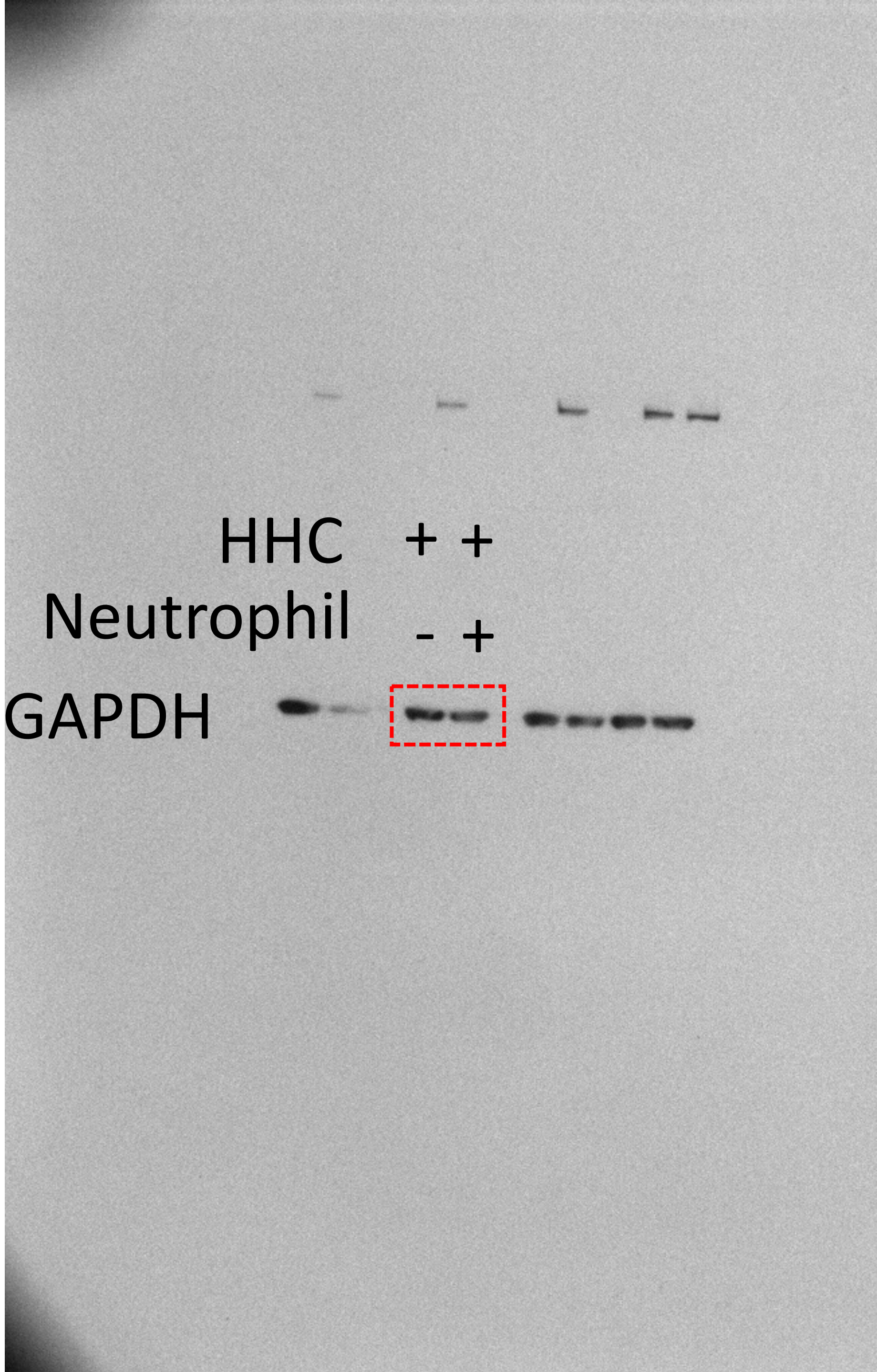

Full unedited blot for Figure S1B

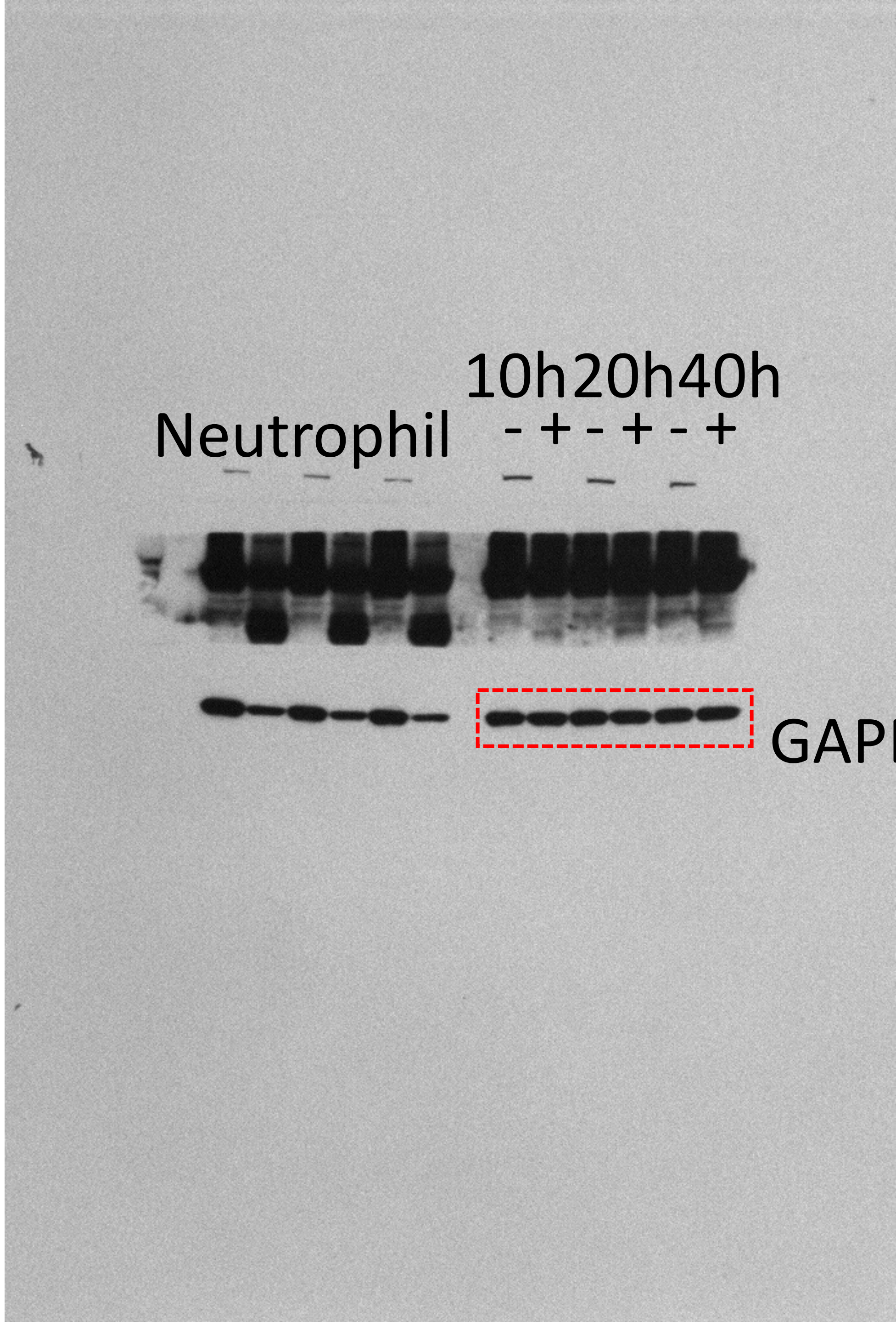

Full unedited blot for Figure S1B

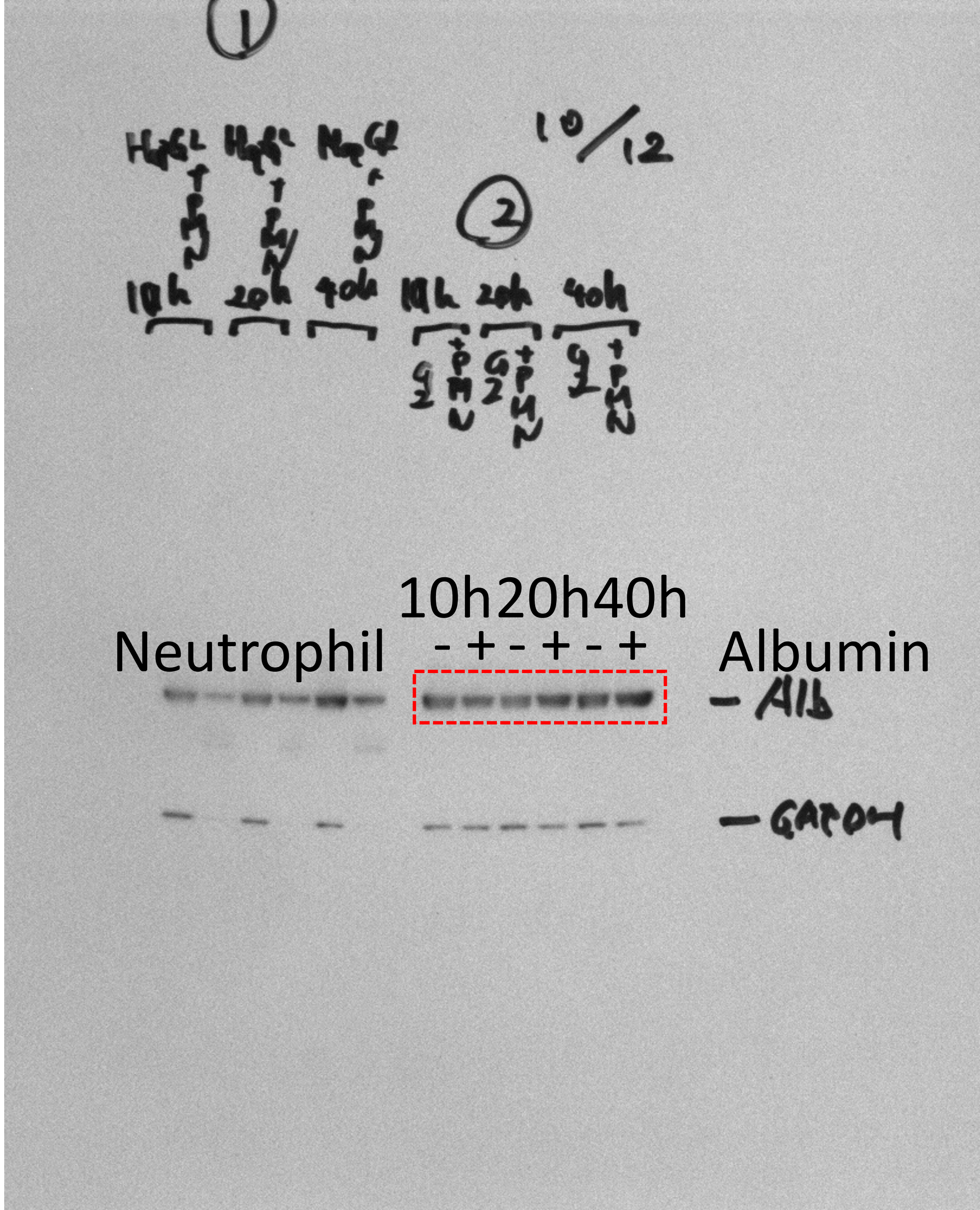

Full unedited blot for Figure S1H

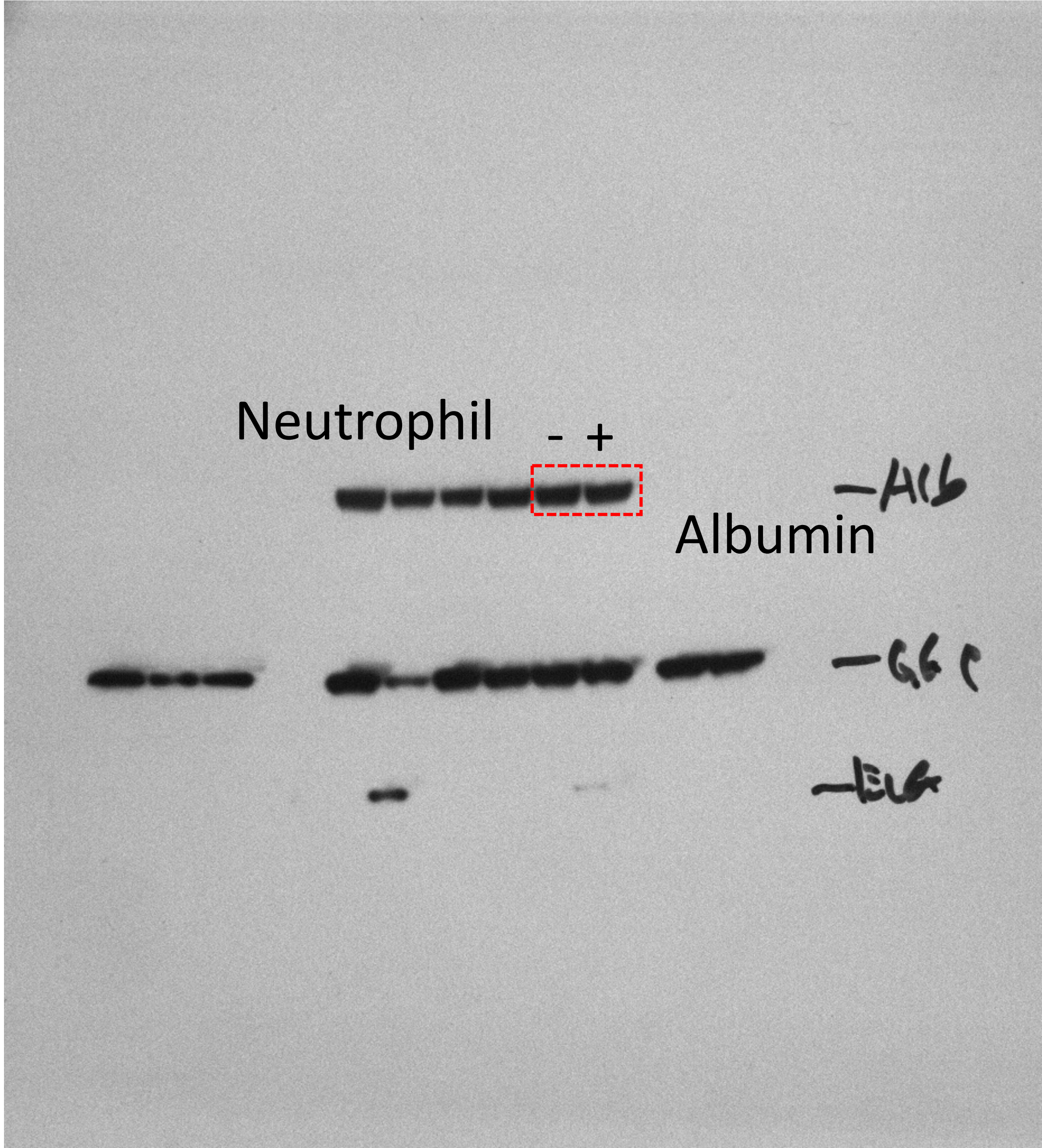

Full unedited blot for Figure S1H

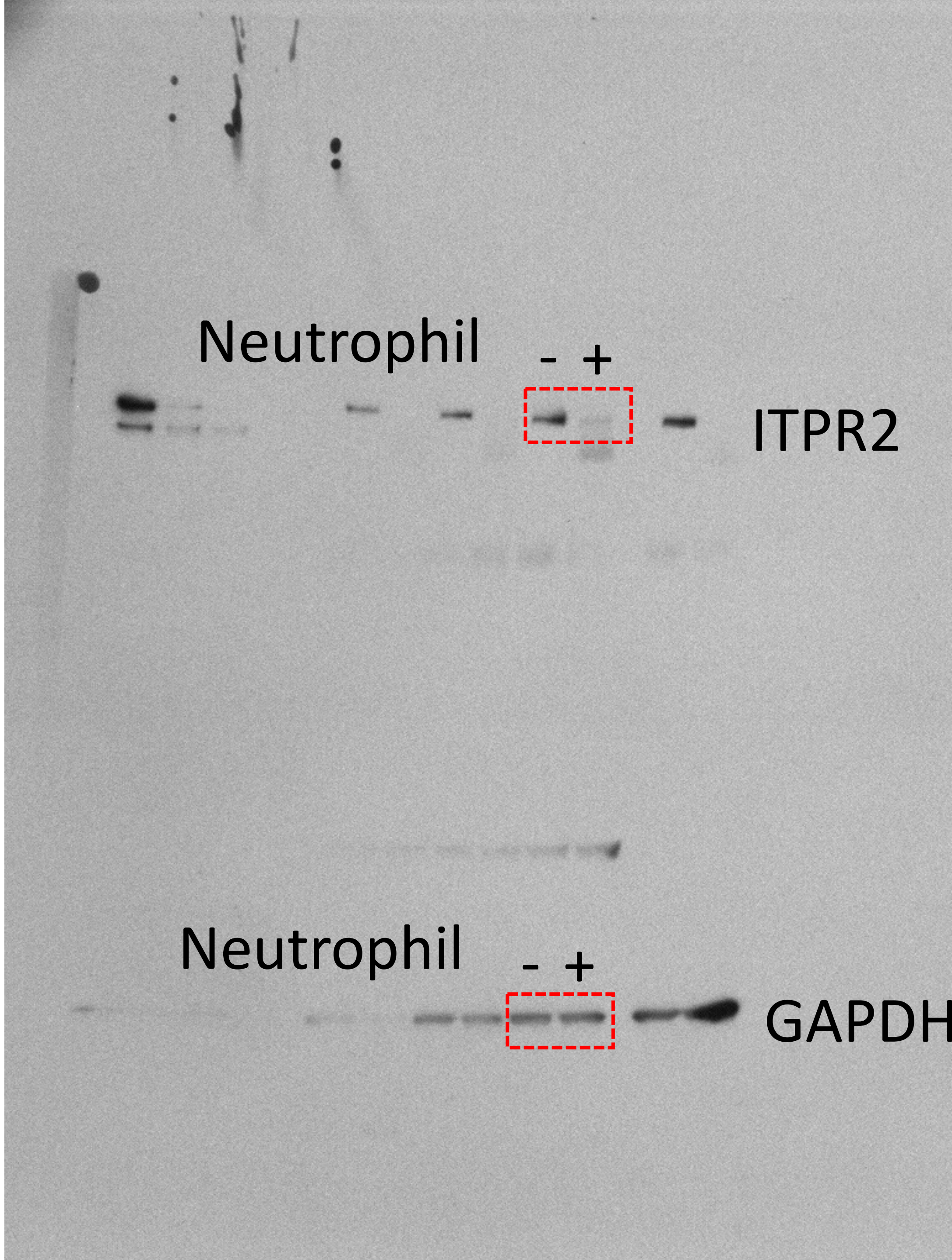

Full unedited blot for Figure 2A

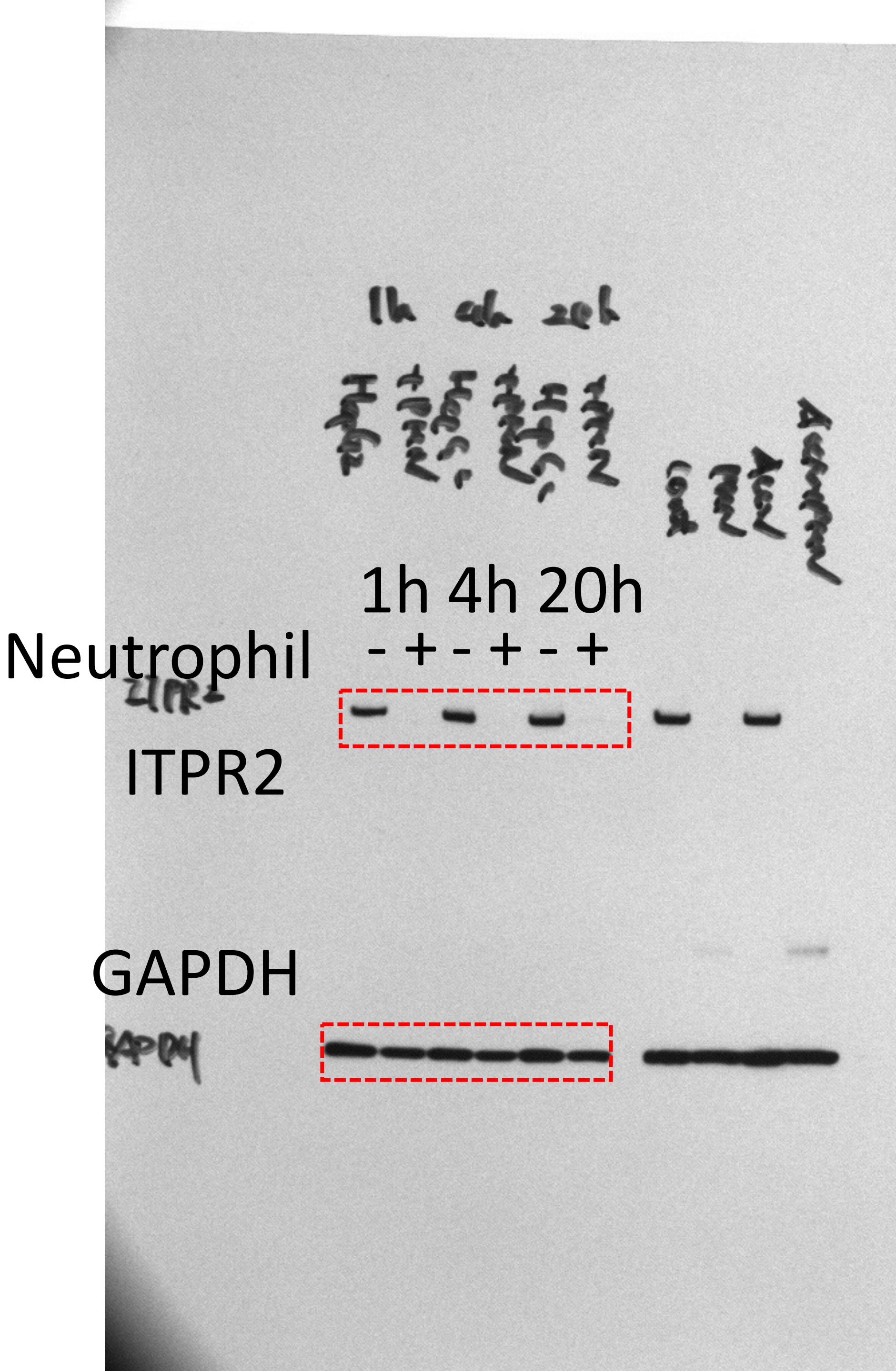

Full unedited blot for Figure 2E

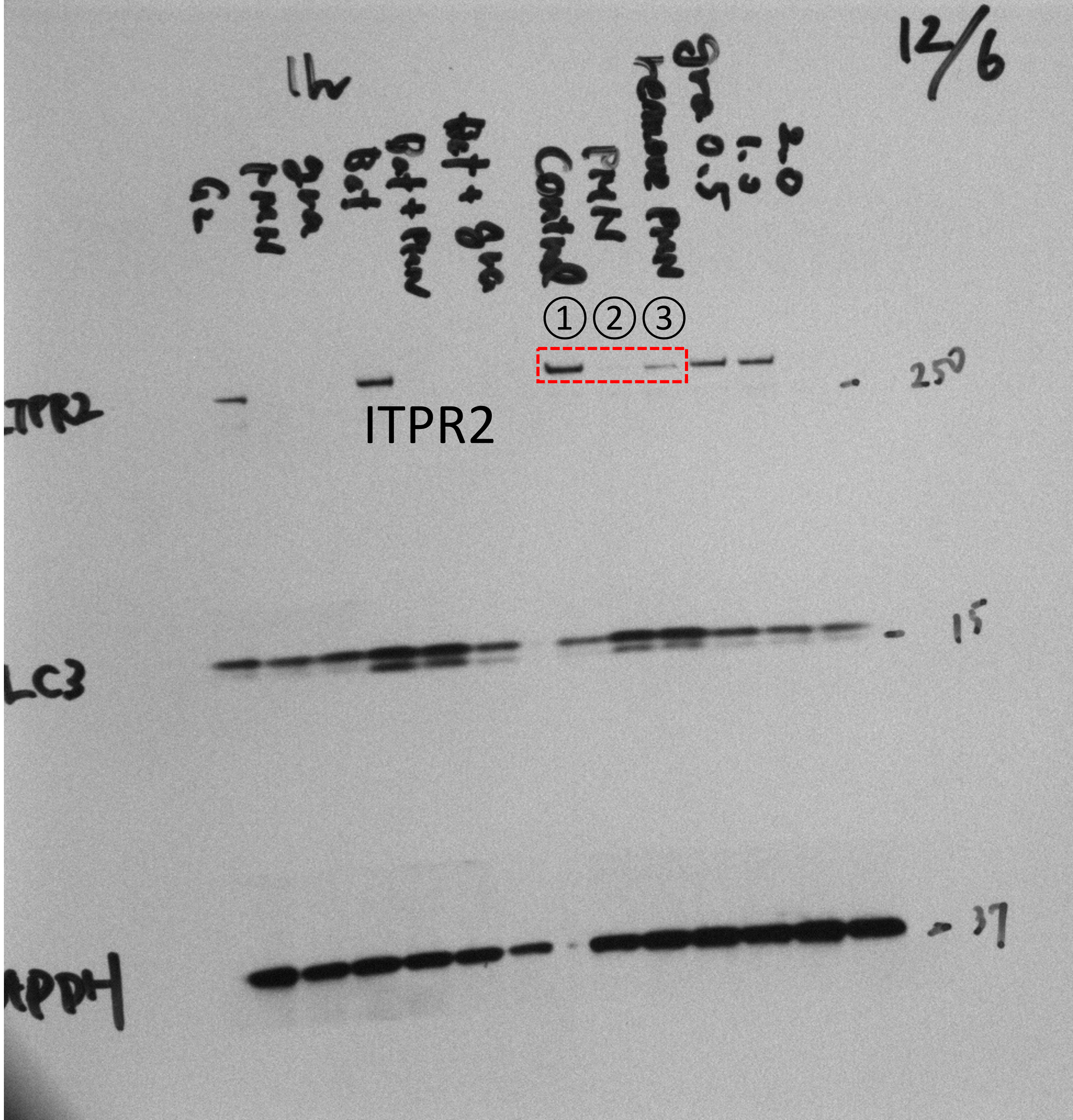

① HepG2

② + Neutrophil

③ Neutrophil removed

Full unedited blot for Figure 2E

① HepG2
② + Neutrophil
③ Neutrophil removed

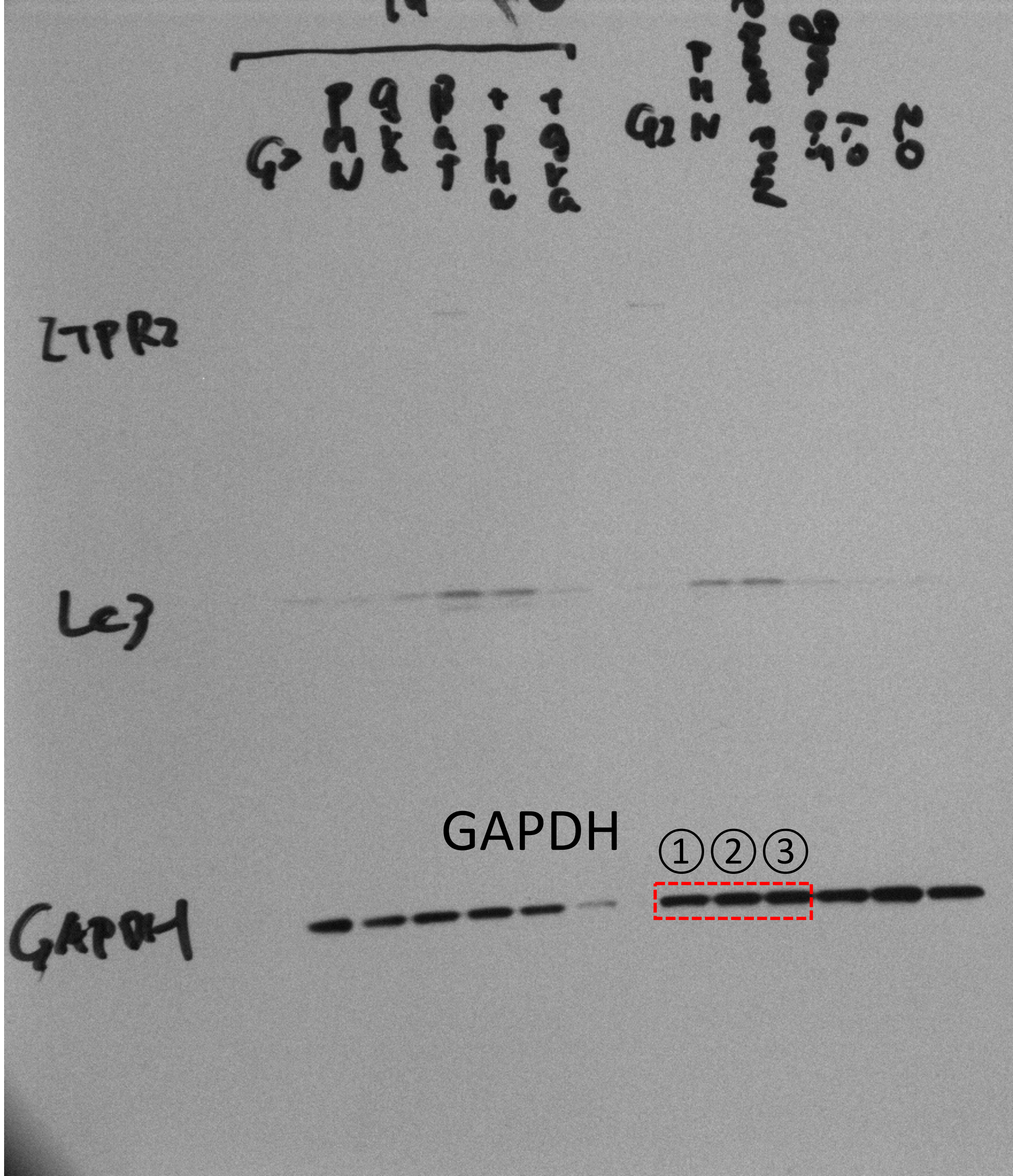

Full unedited blot for Figure 2H

① + MG132

② + Baf

③ + ALLN

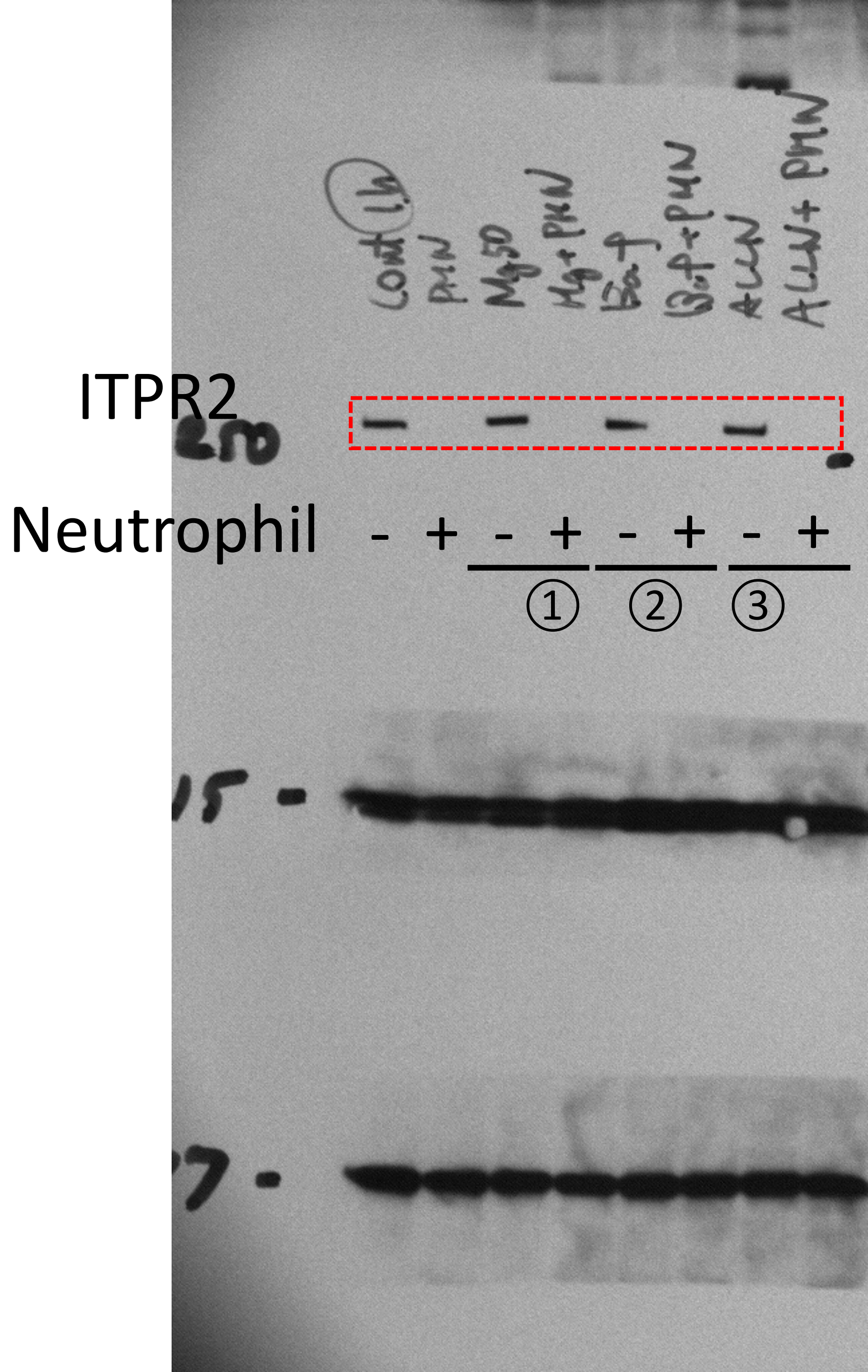

Full unedited blot for Figure 2H

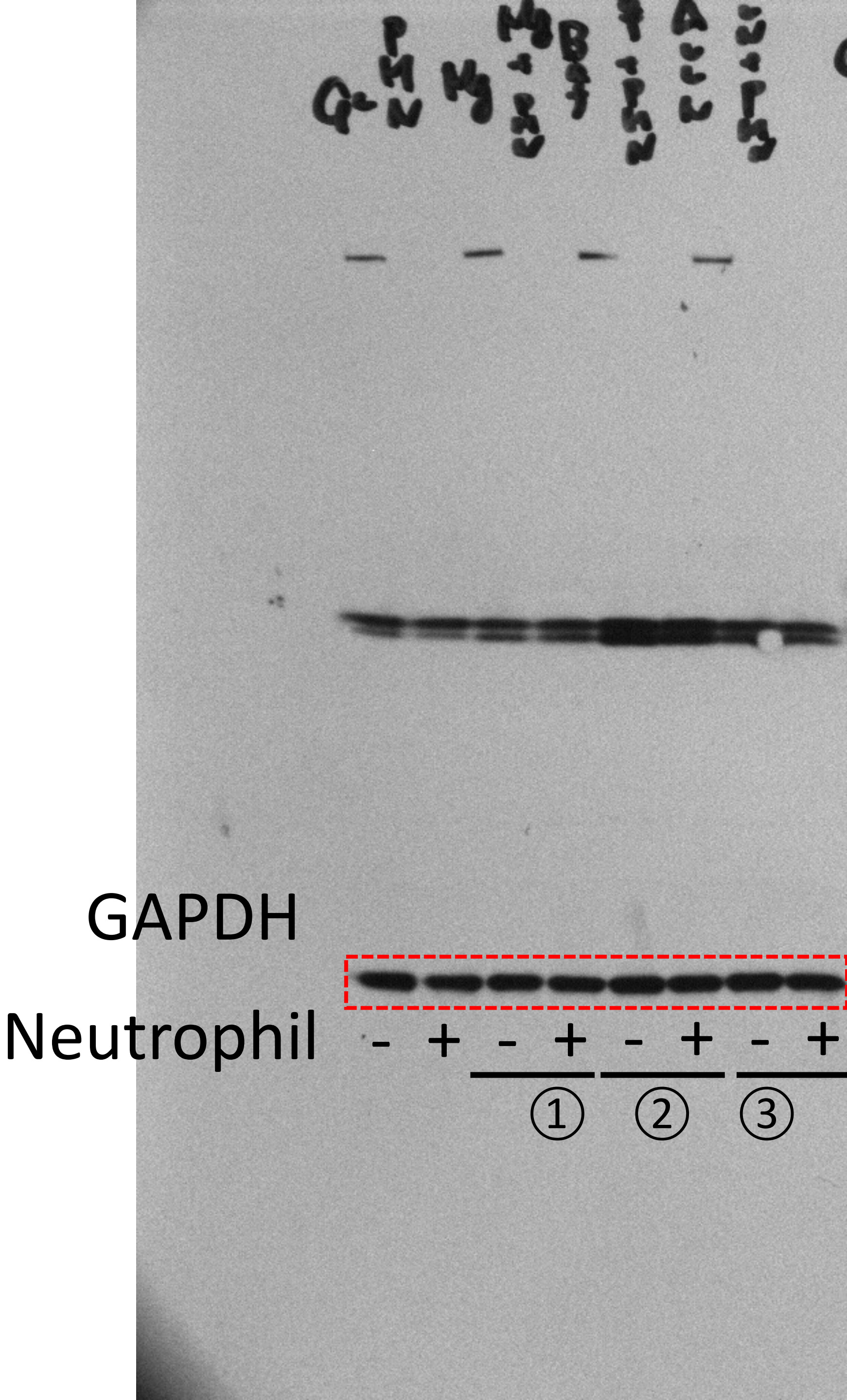

① + MG132
② + Baf
③ + ALLN

Full unedited blot for Figure S2C

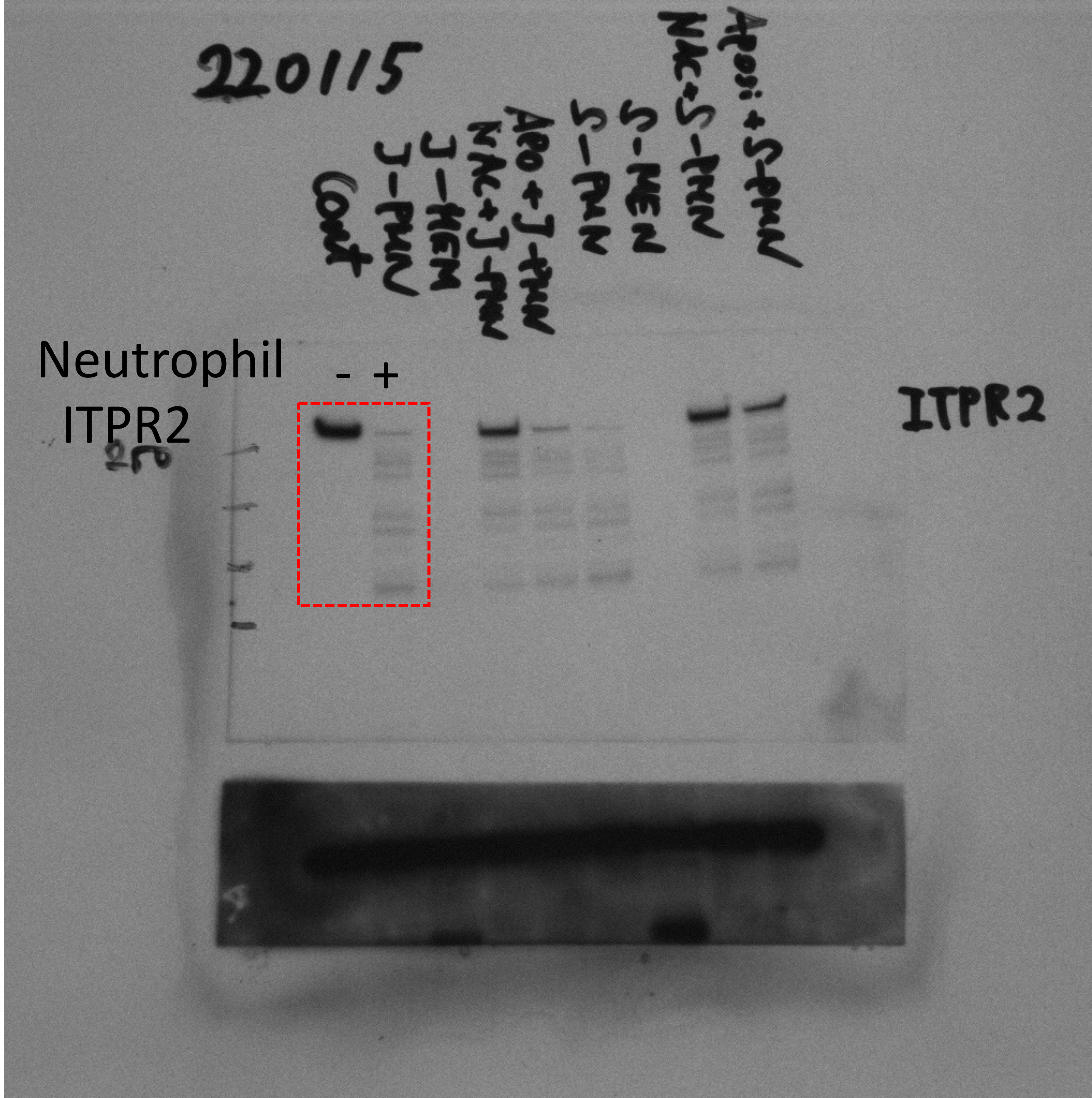

Full unedited blot for Figure S2C

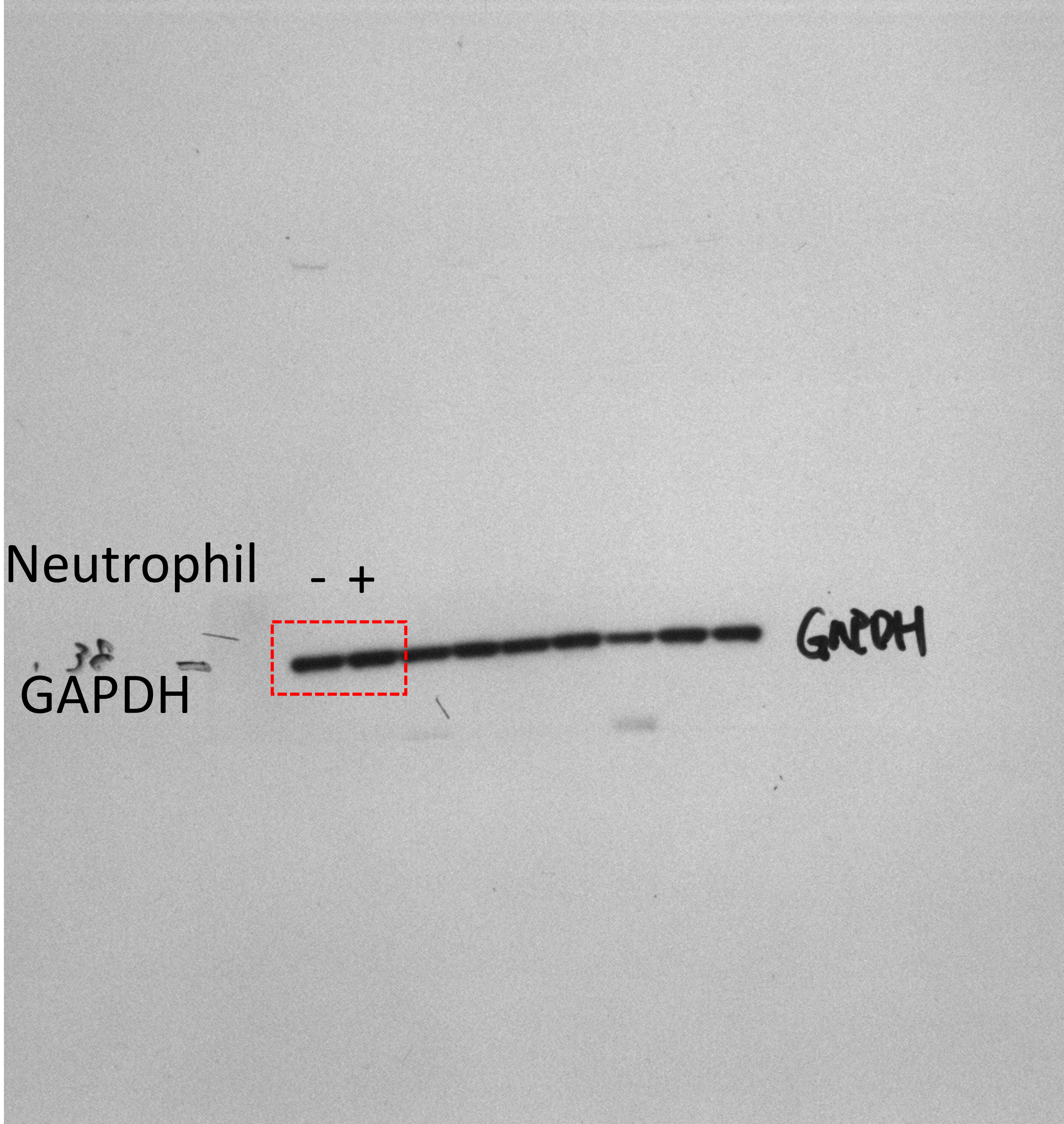

Full unedited blot for Figure S2D

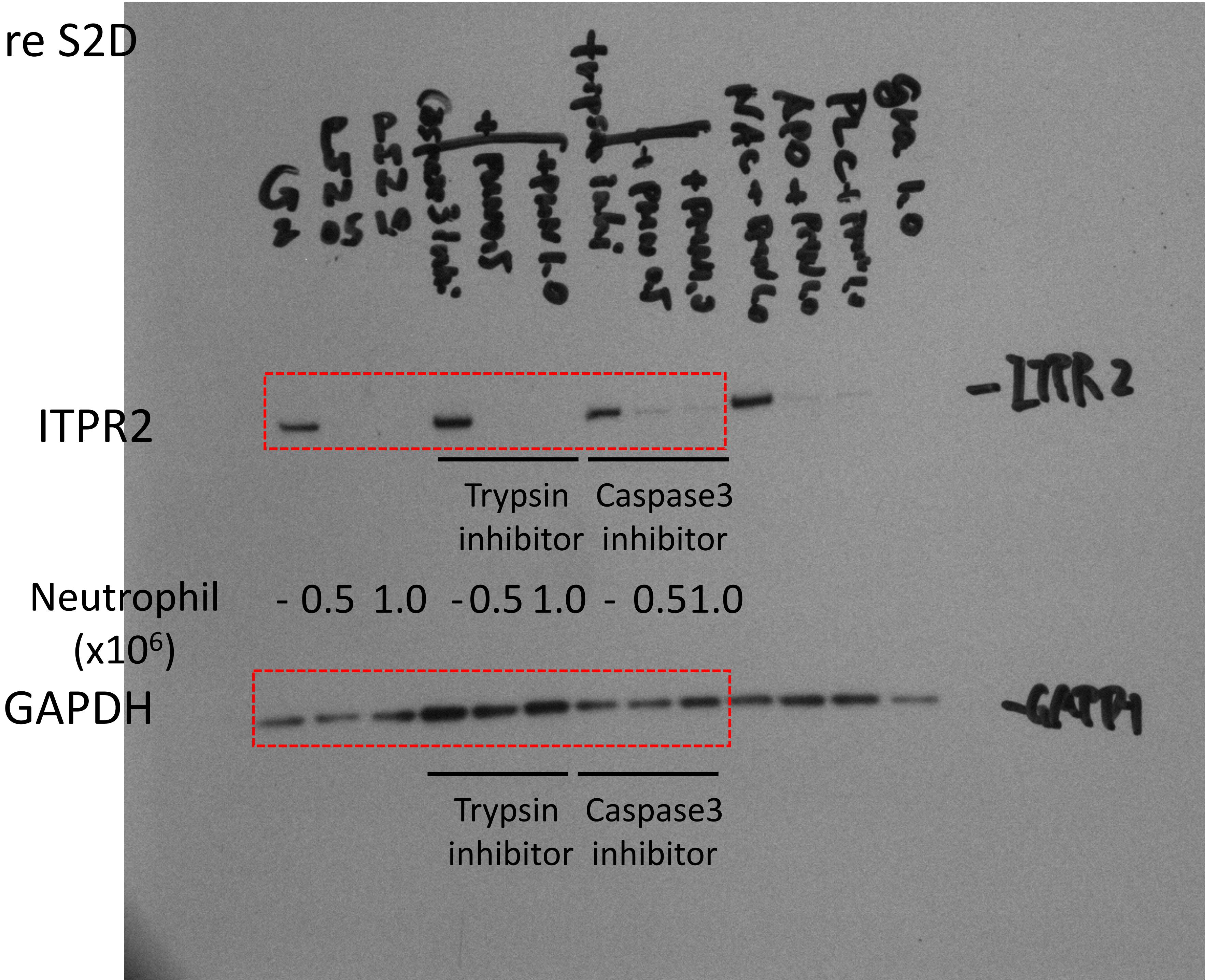

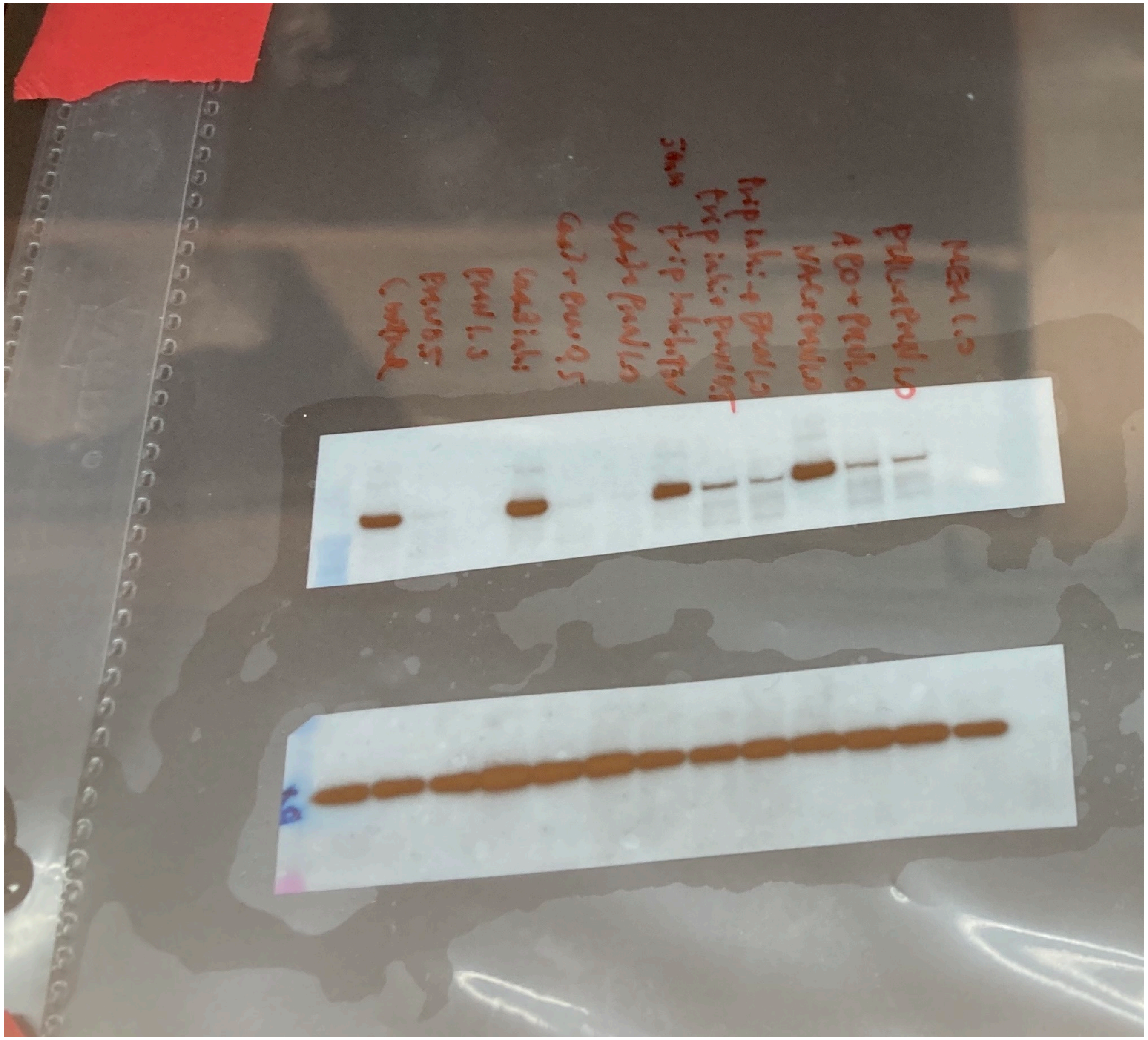

Full unedited blot for Figure 3A, C

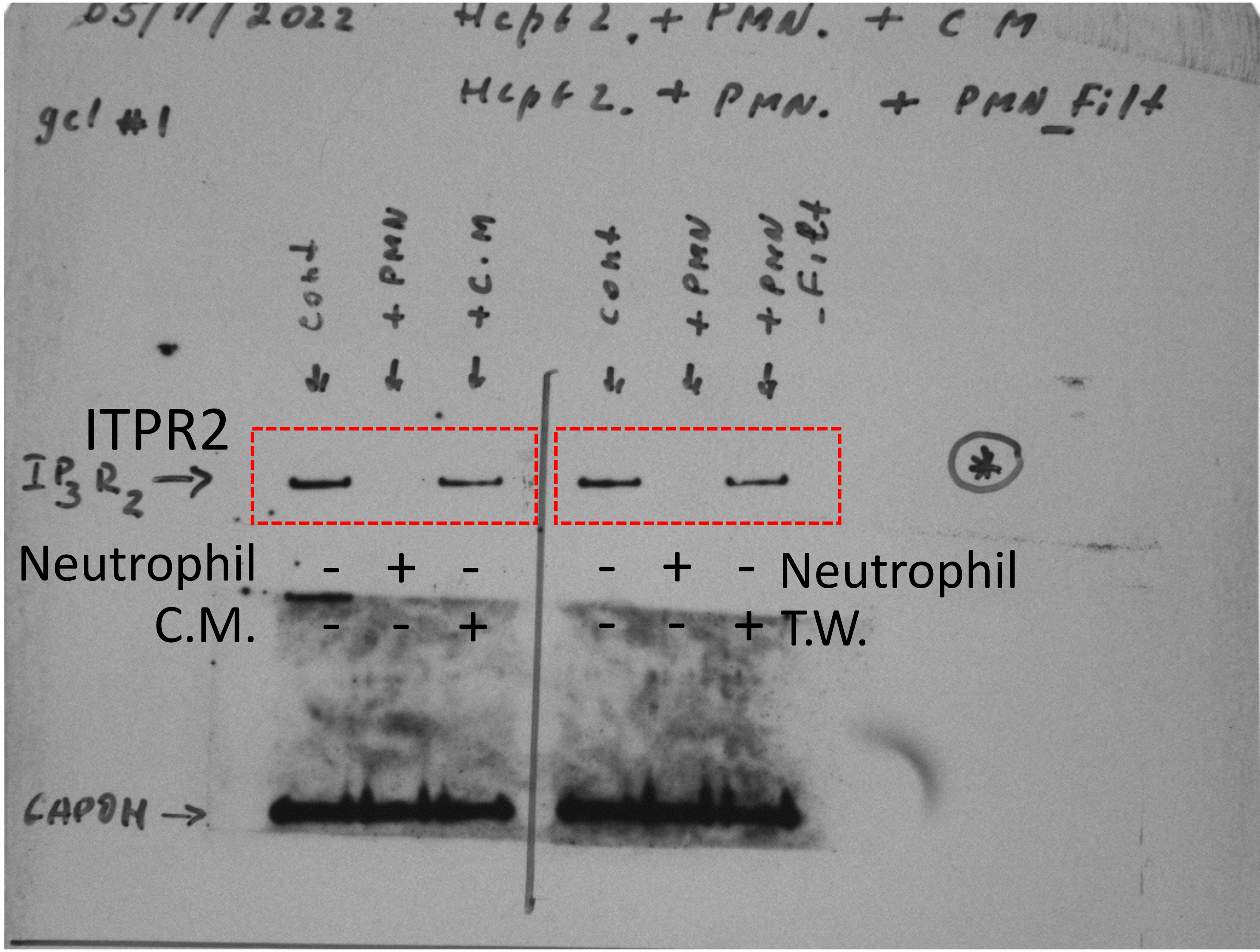

Full unedited blot for Figure 3A, C

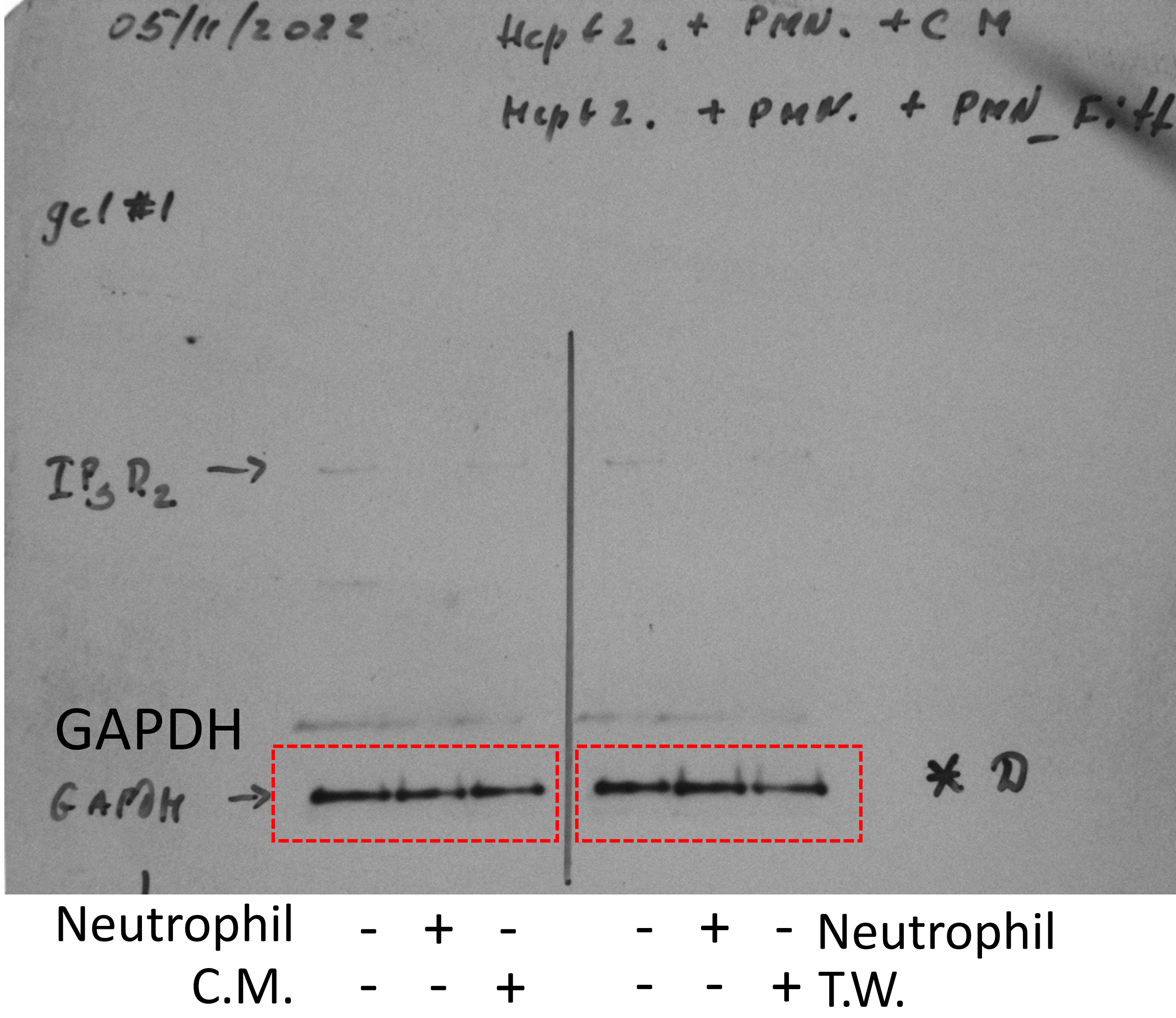

Full unedited blot for Figure 3E

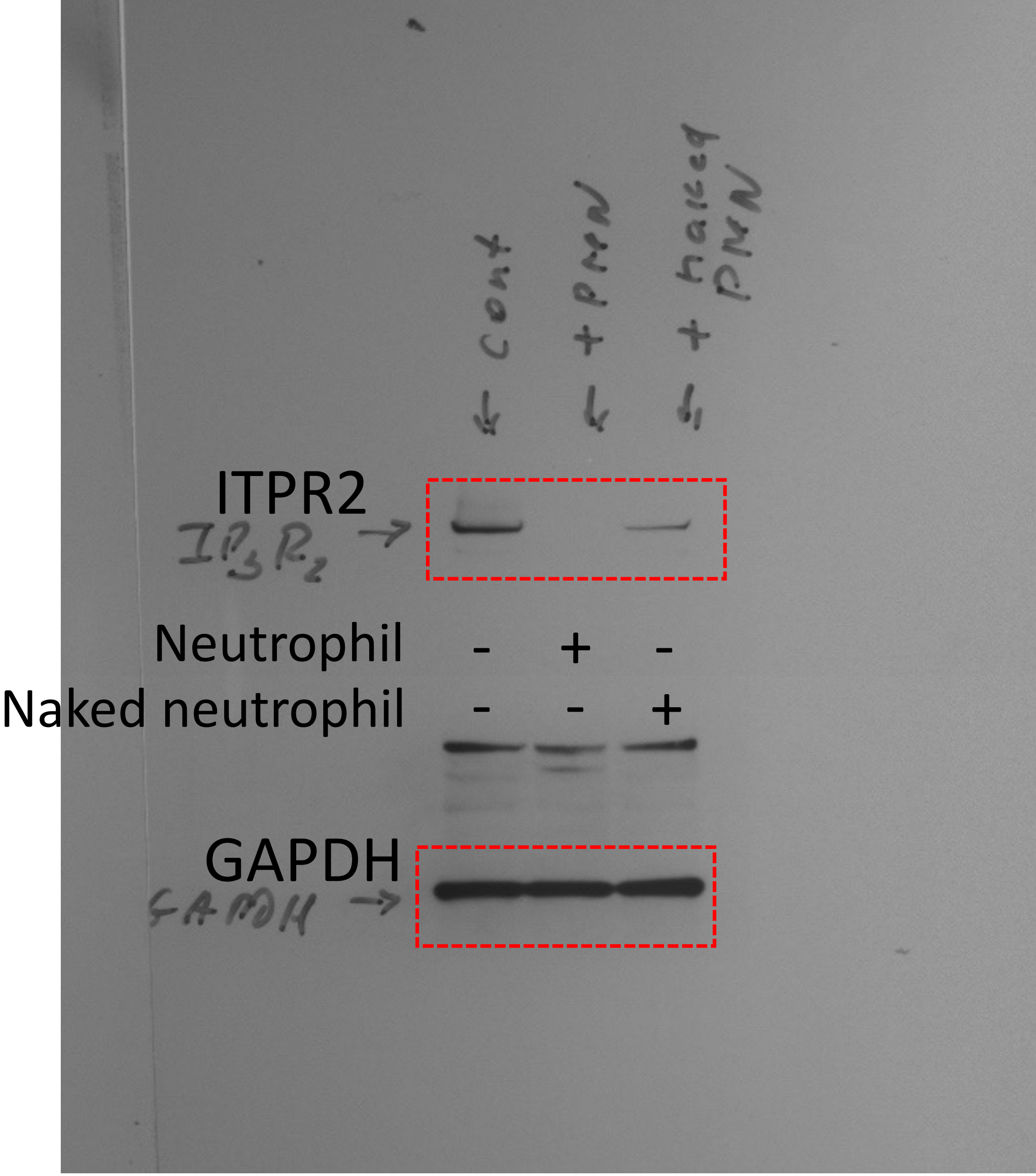

Full unedited blot for Figure 3G

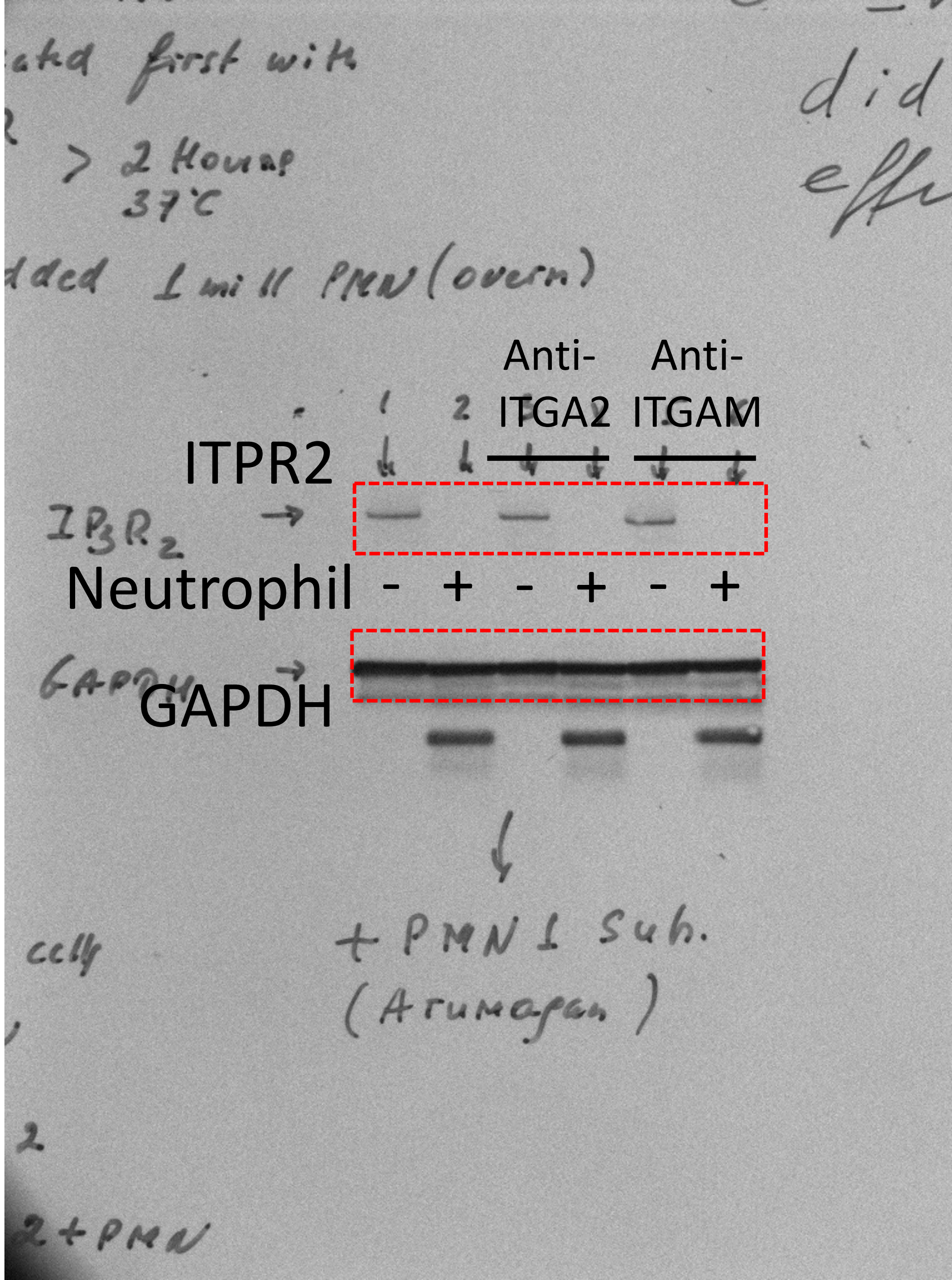

Full unedited blot for Figure 3I

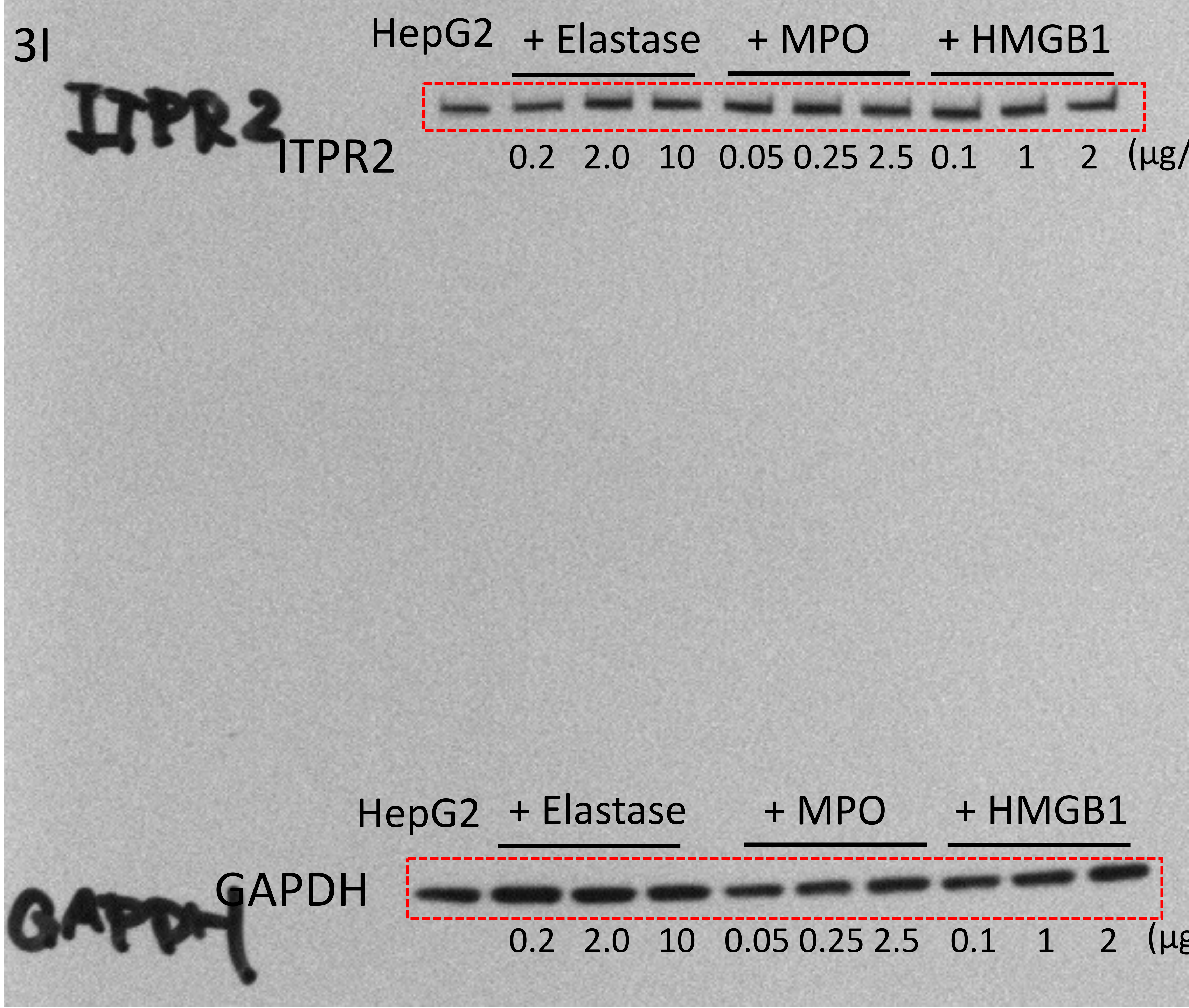

Full unedited blot for Figure S3D

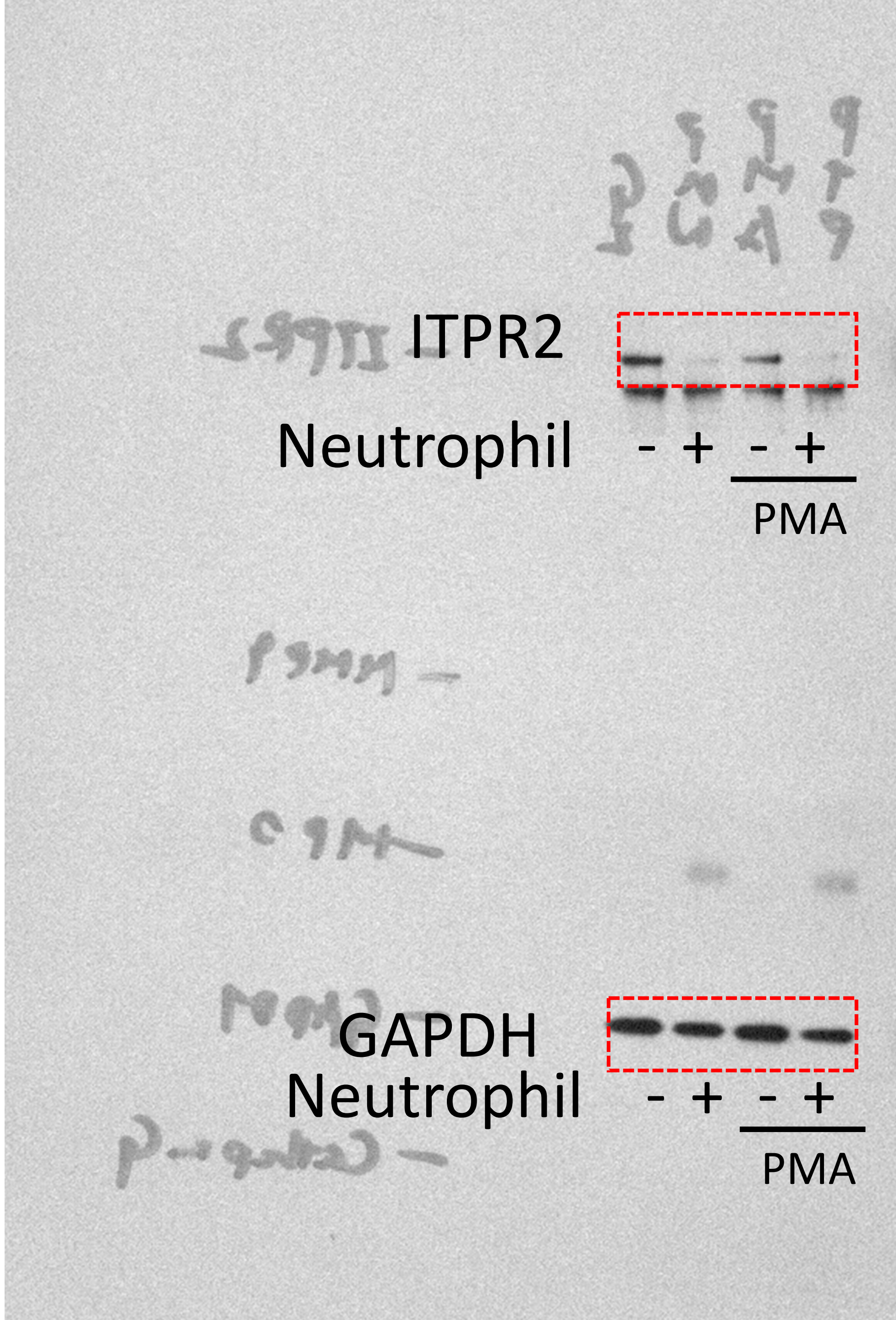

Full unedited blot for Figure S3H

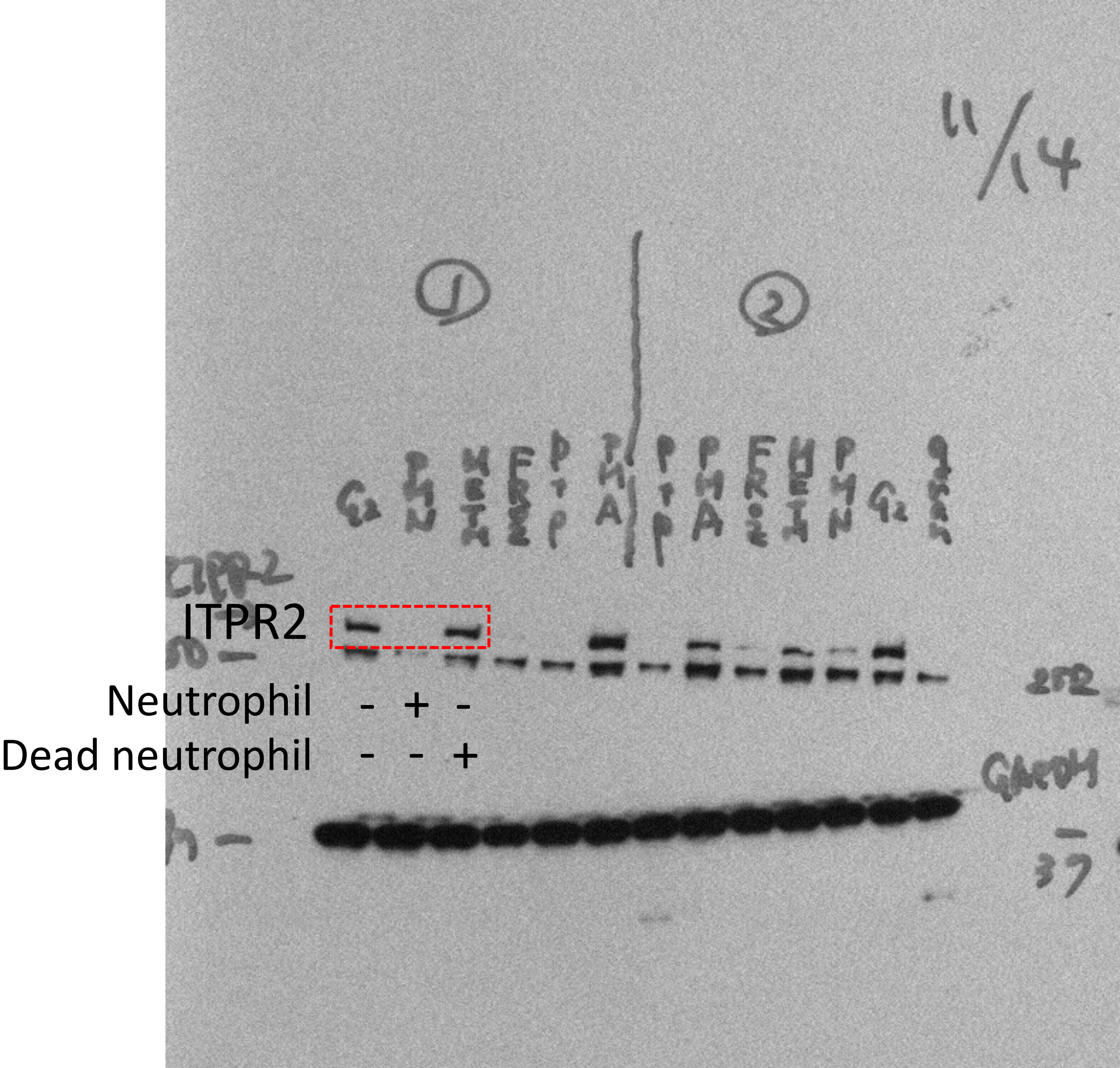

Full unedited blot for Figure S3H

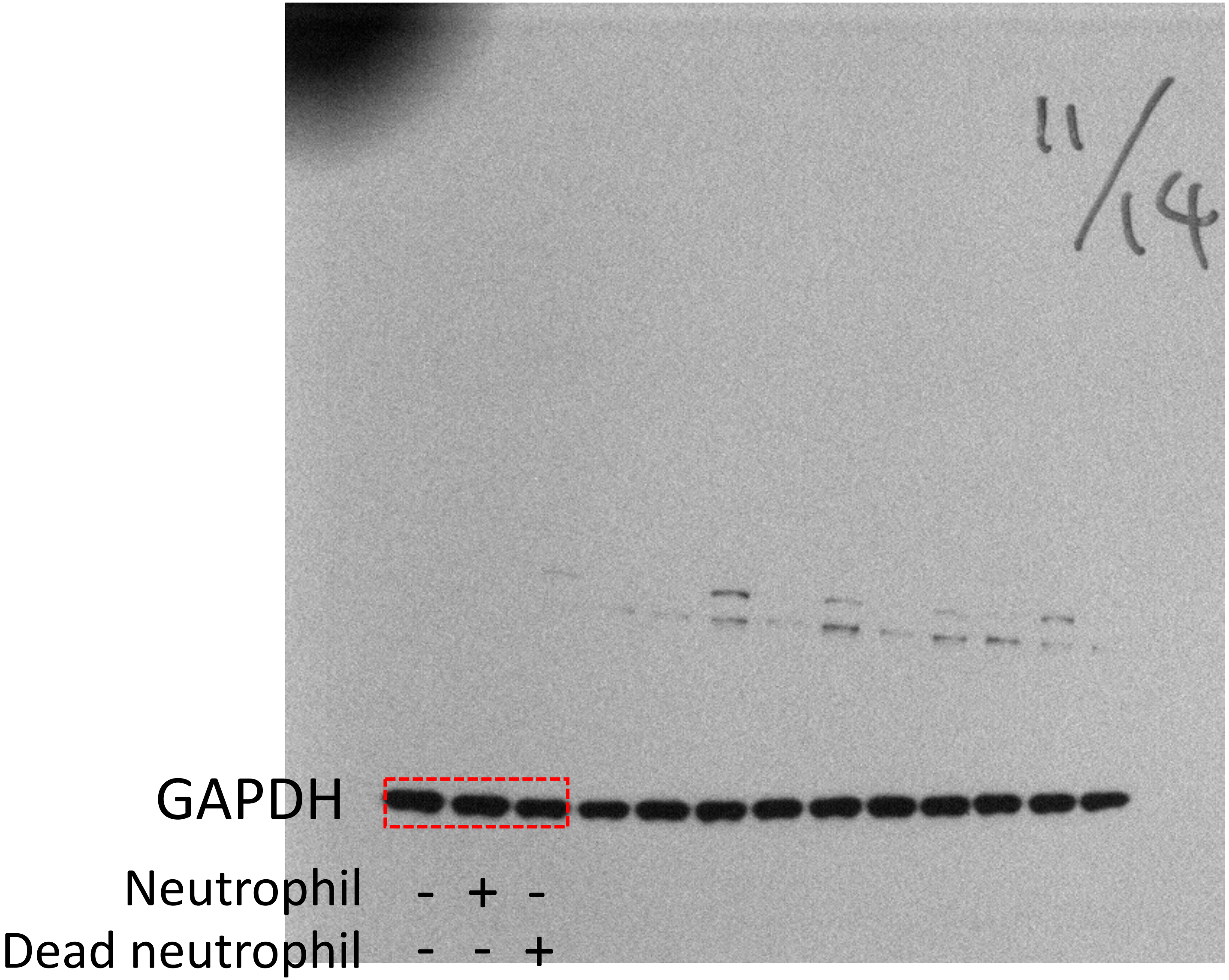

Full unedited blot for Figure 4B

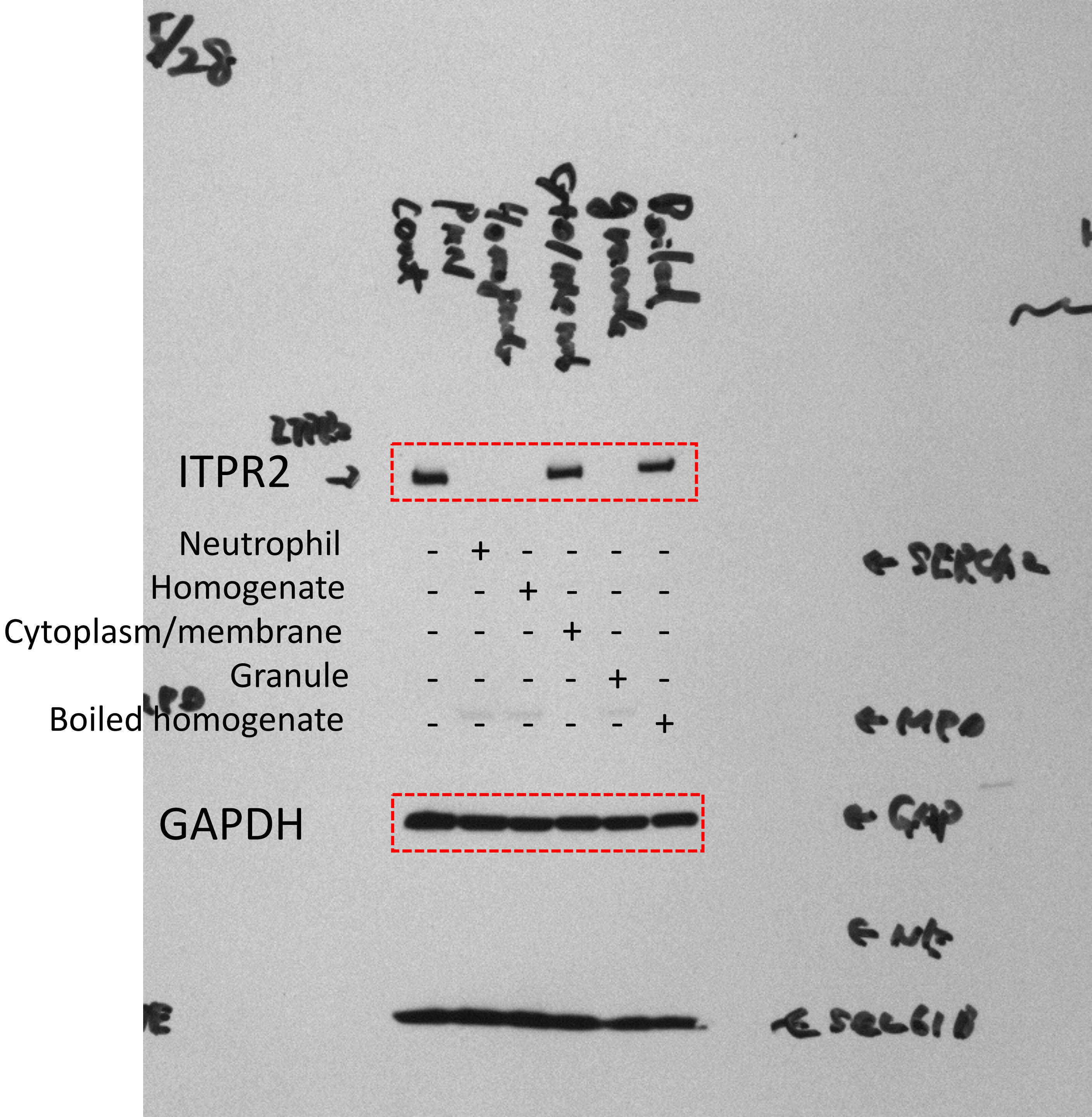

Full unedited blot for Figure 4F

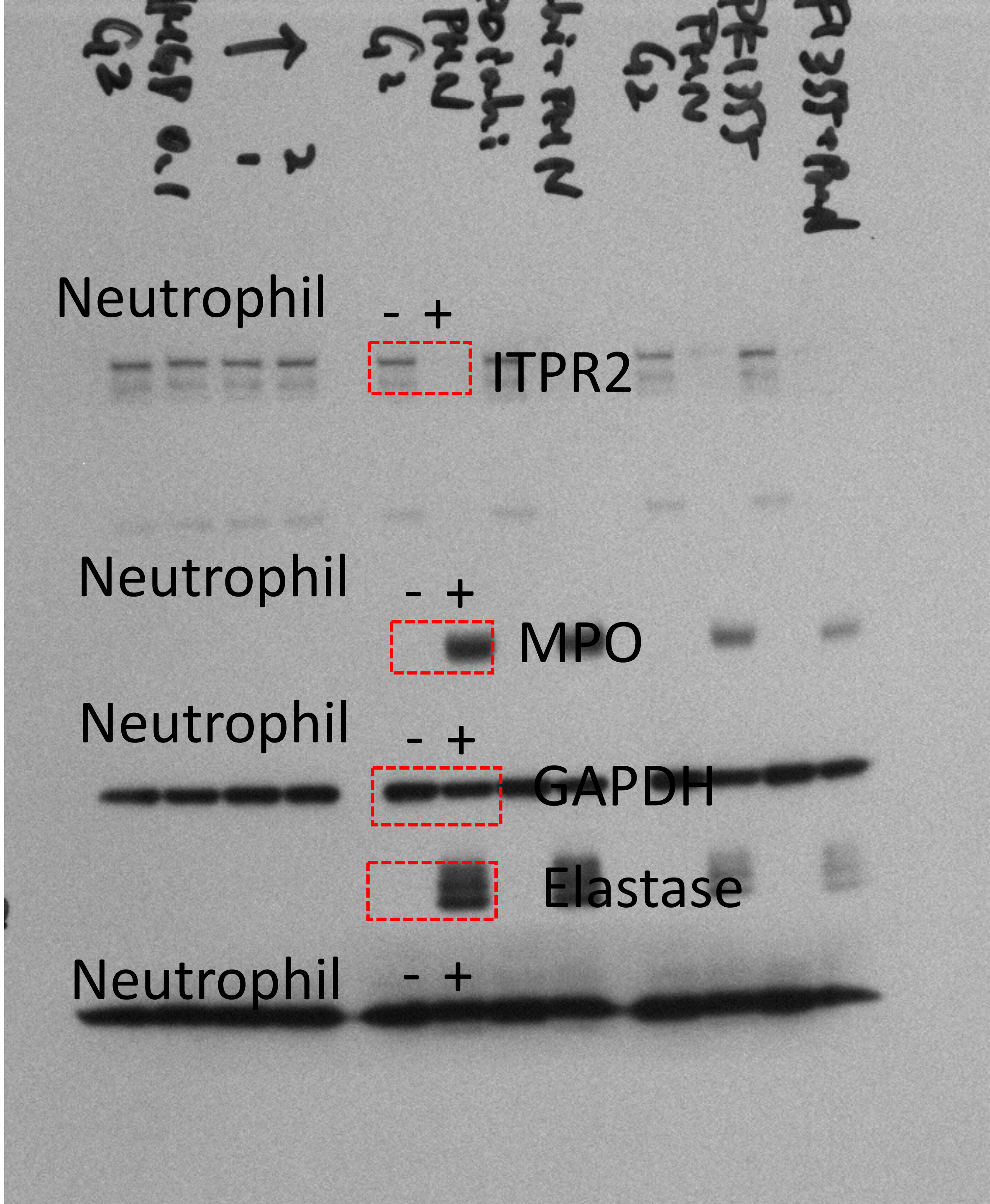

Full unedited blot for Figure S4A

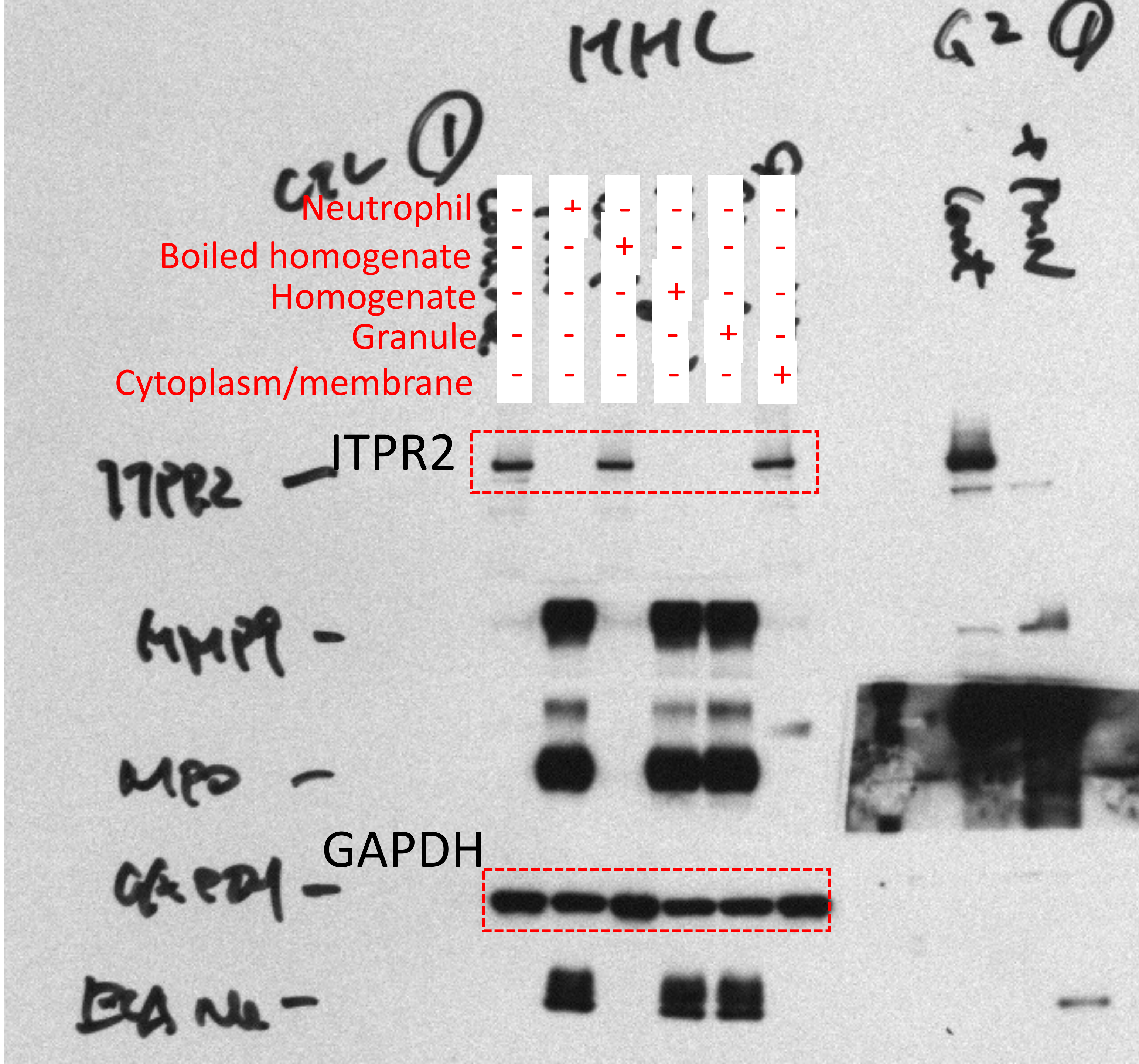

Full unedited blot for Figure S4E

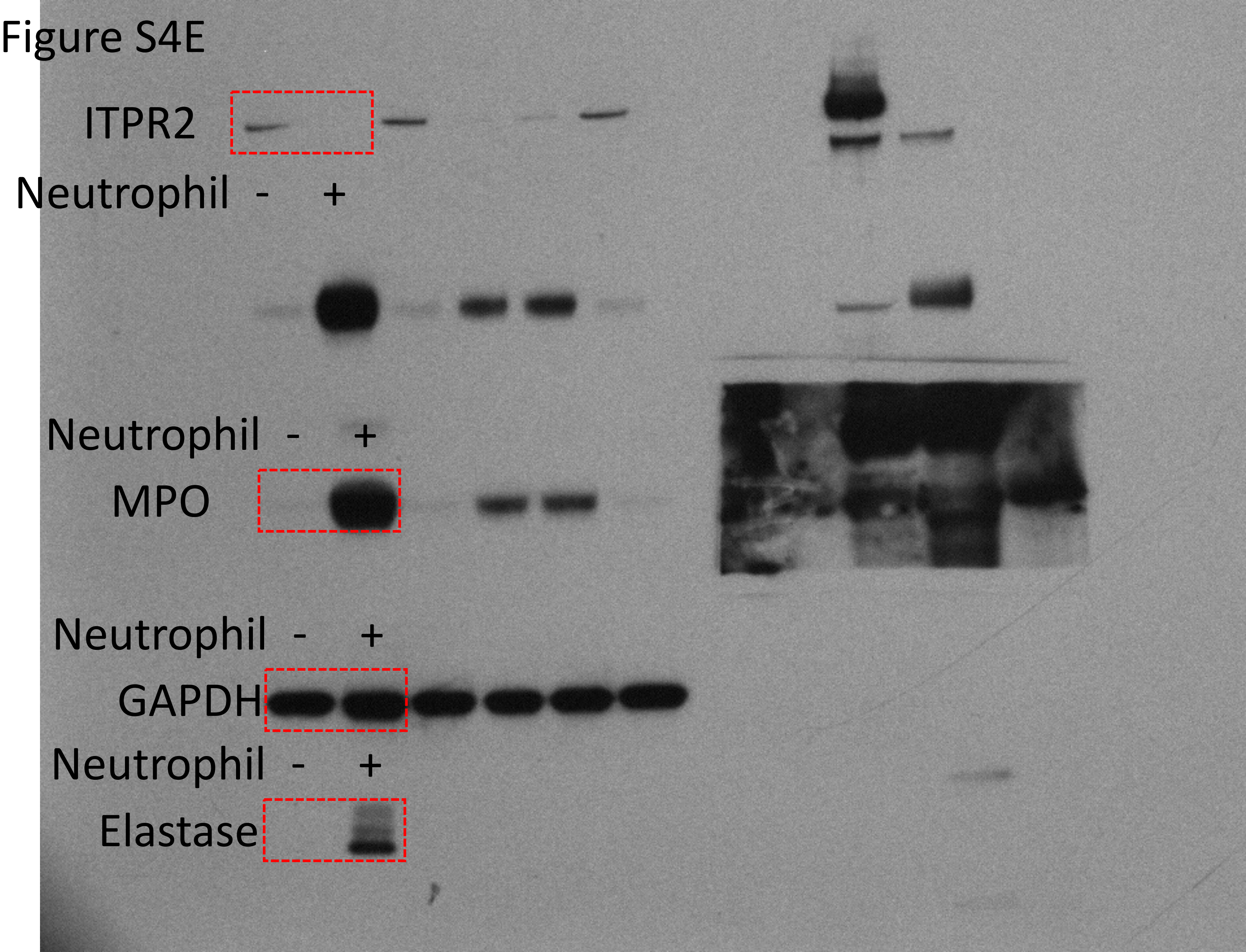

Full unedited blot for Figure S4I

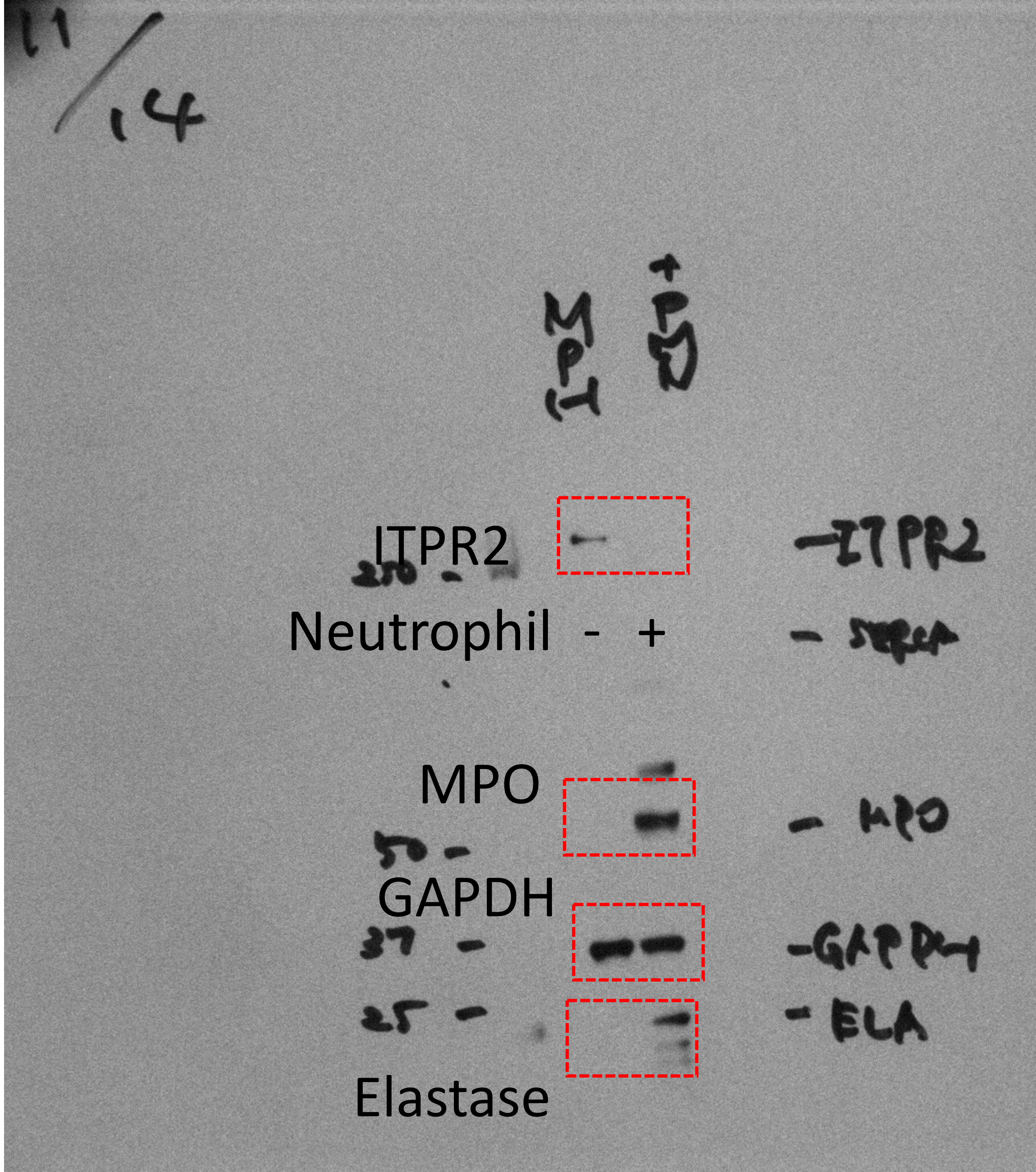

Full unedited blot for Figure 5A

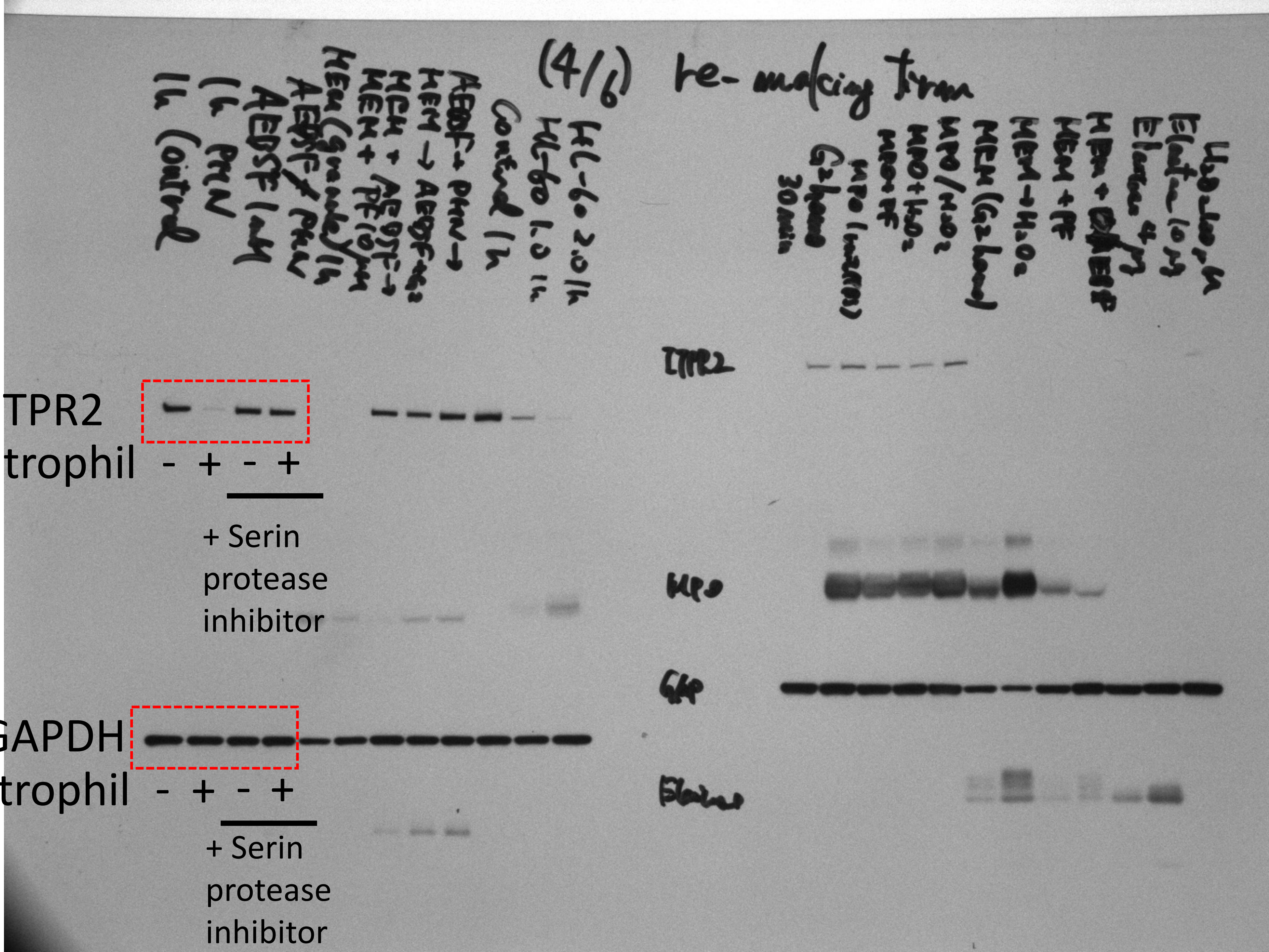

Full unedited blot for Figure 5C

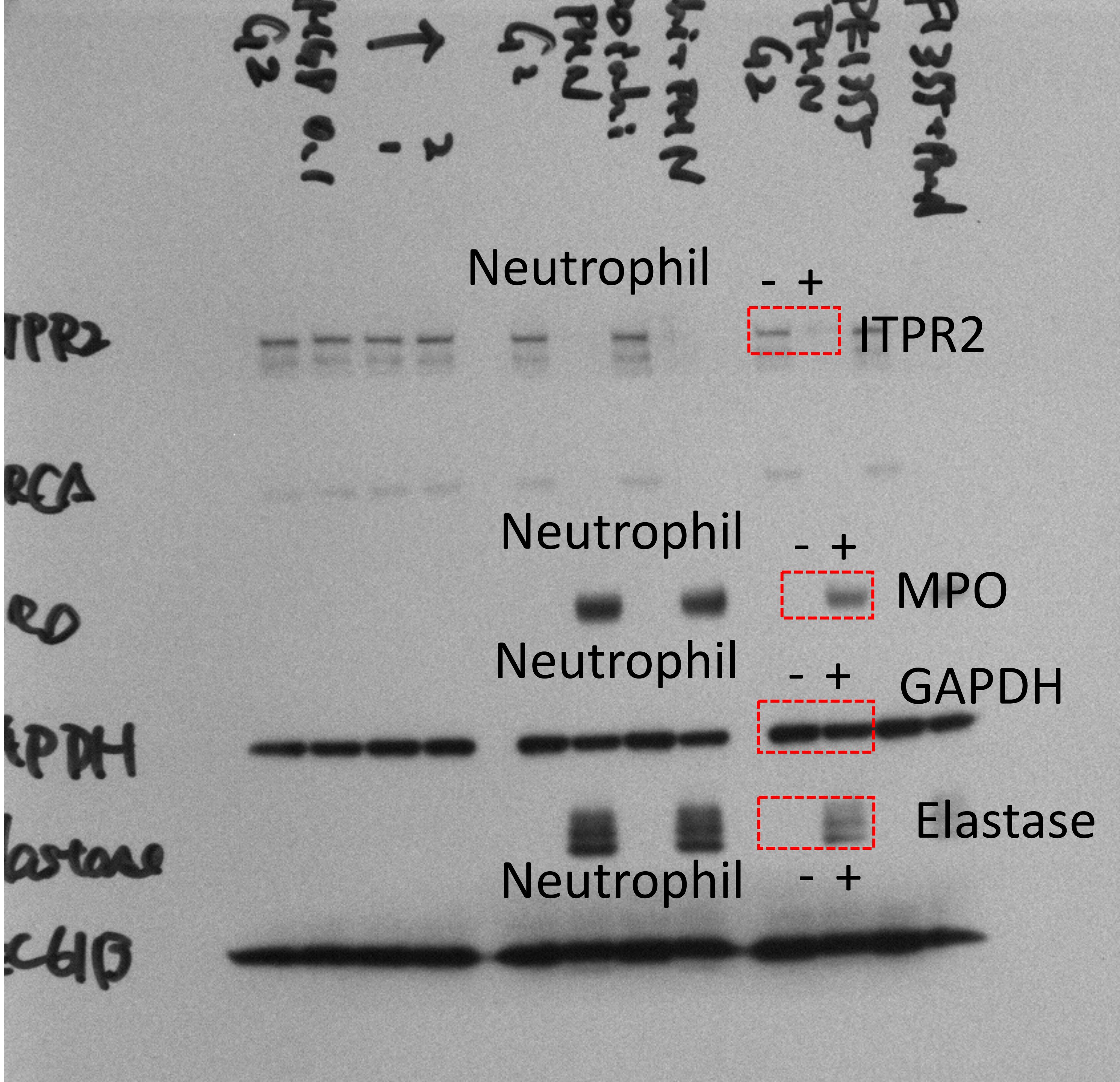

Full unedited blot for Figure 5E

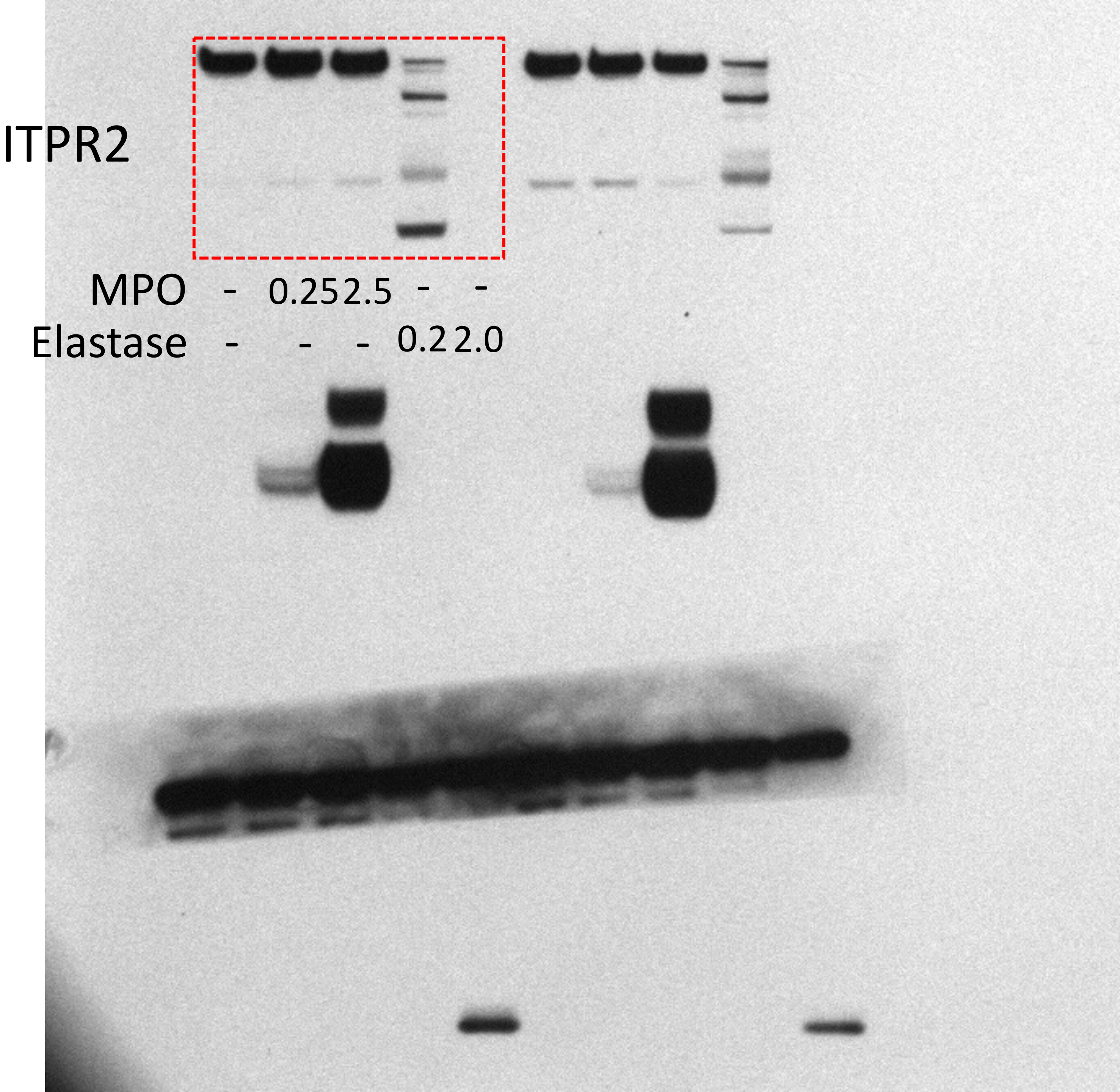

Full unedited blot for Figure 5E

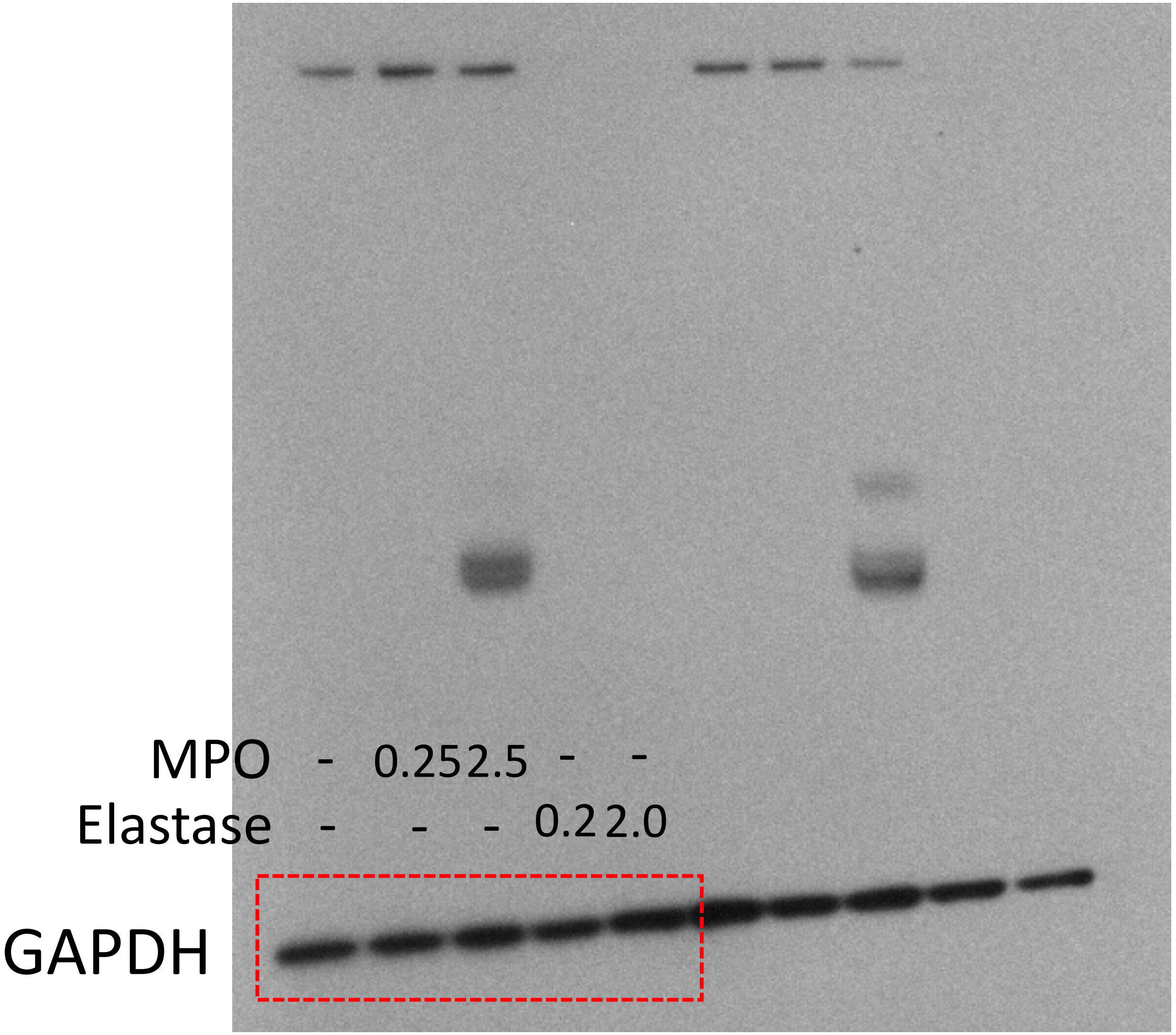

Full unedited blot
for Figure 5J

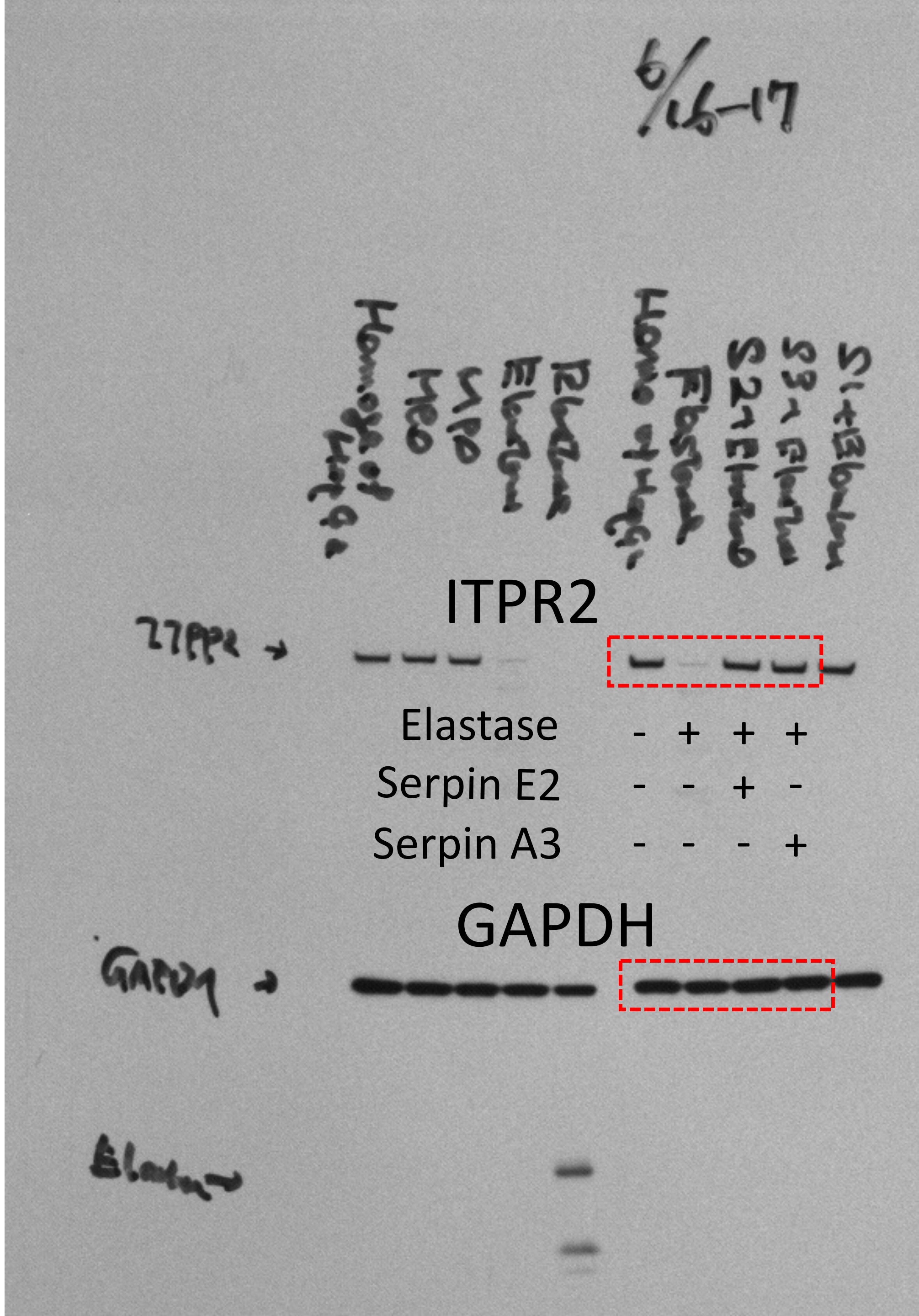

Full unedited blot
for Figure S5A

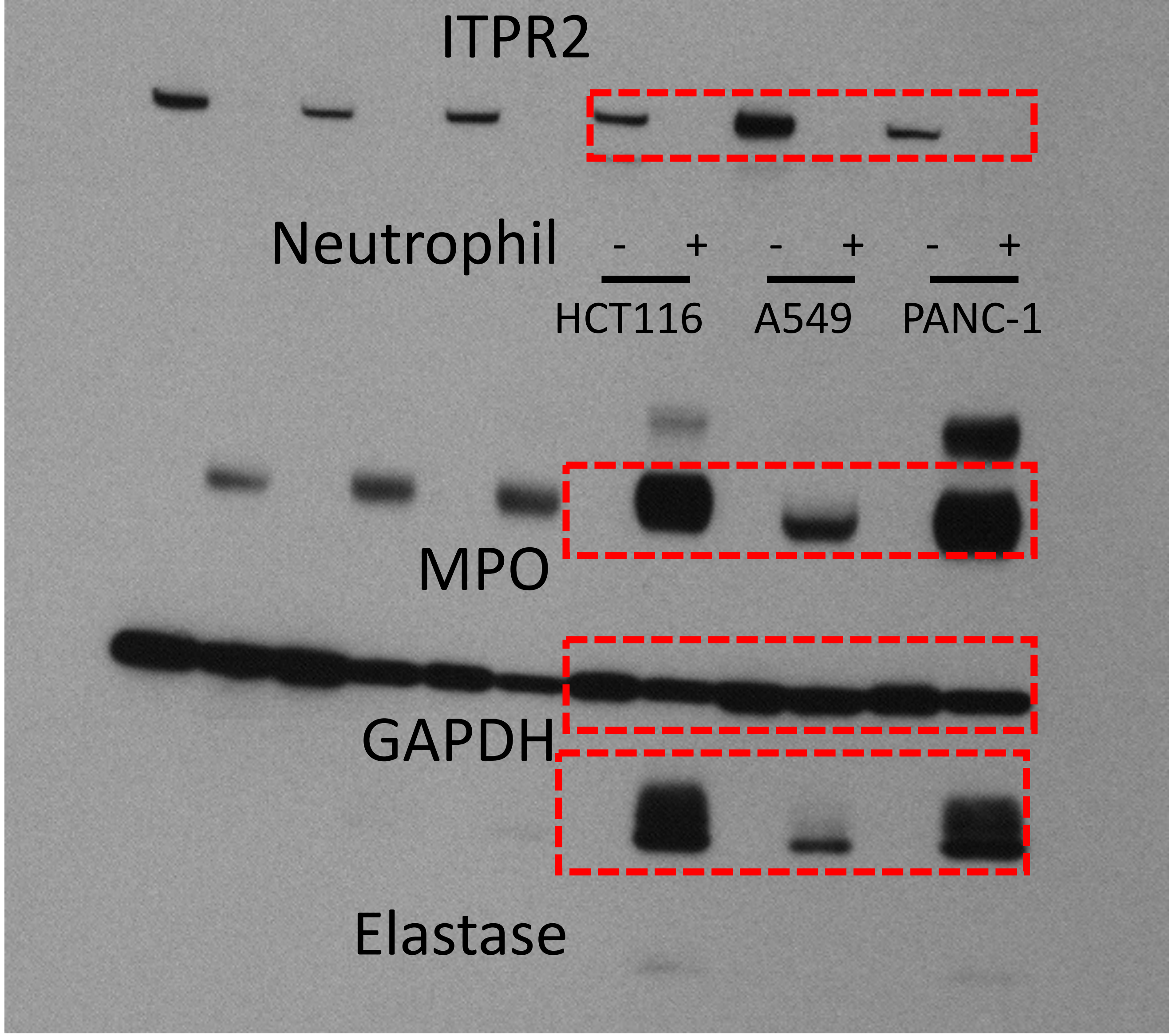

Full unedited blot for Figure S5C

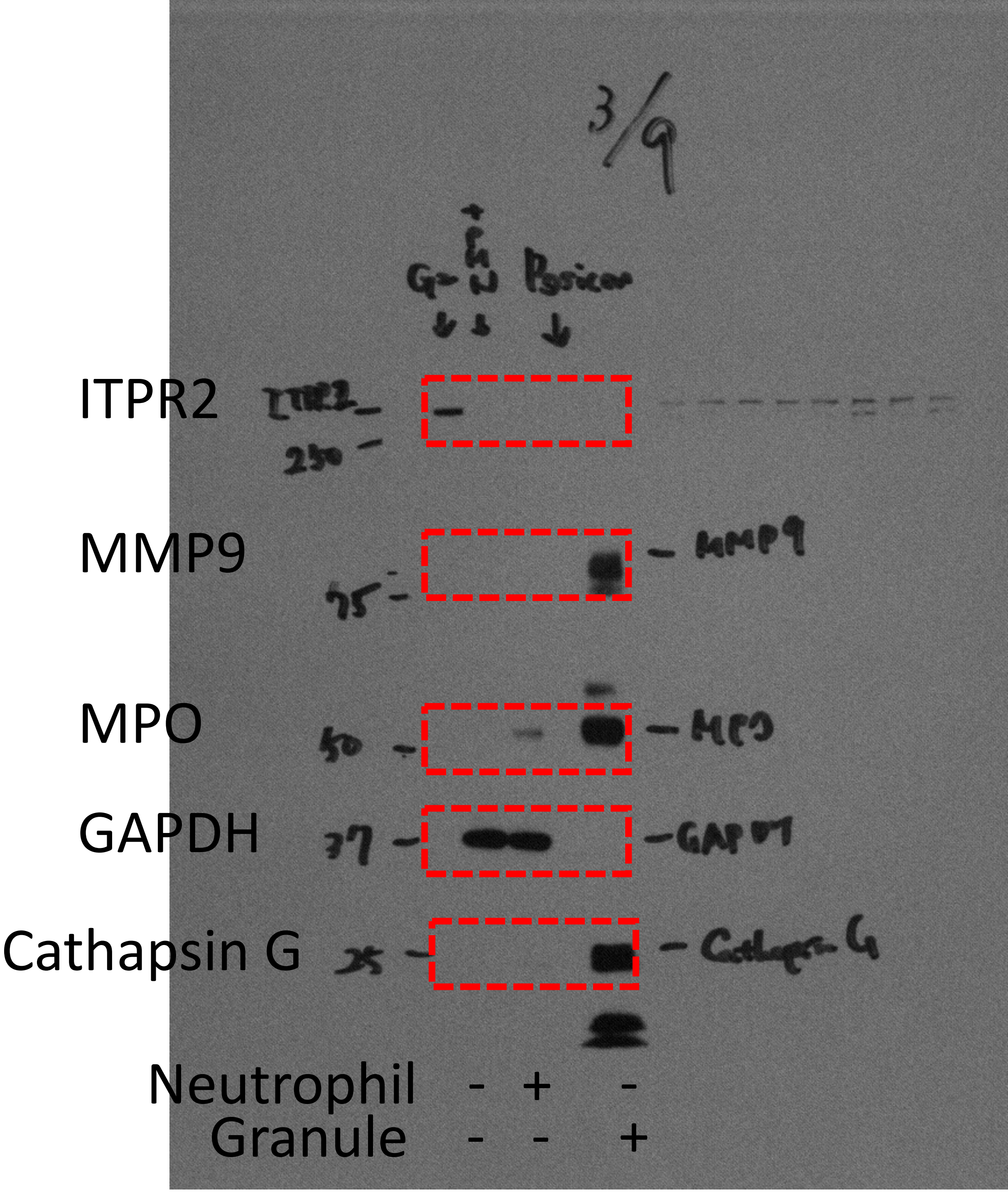

Full unedited blot
for Figure S5C

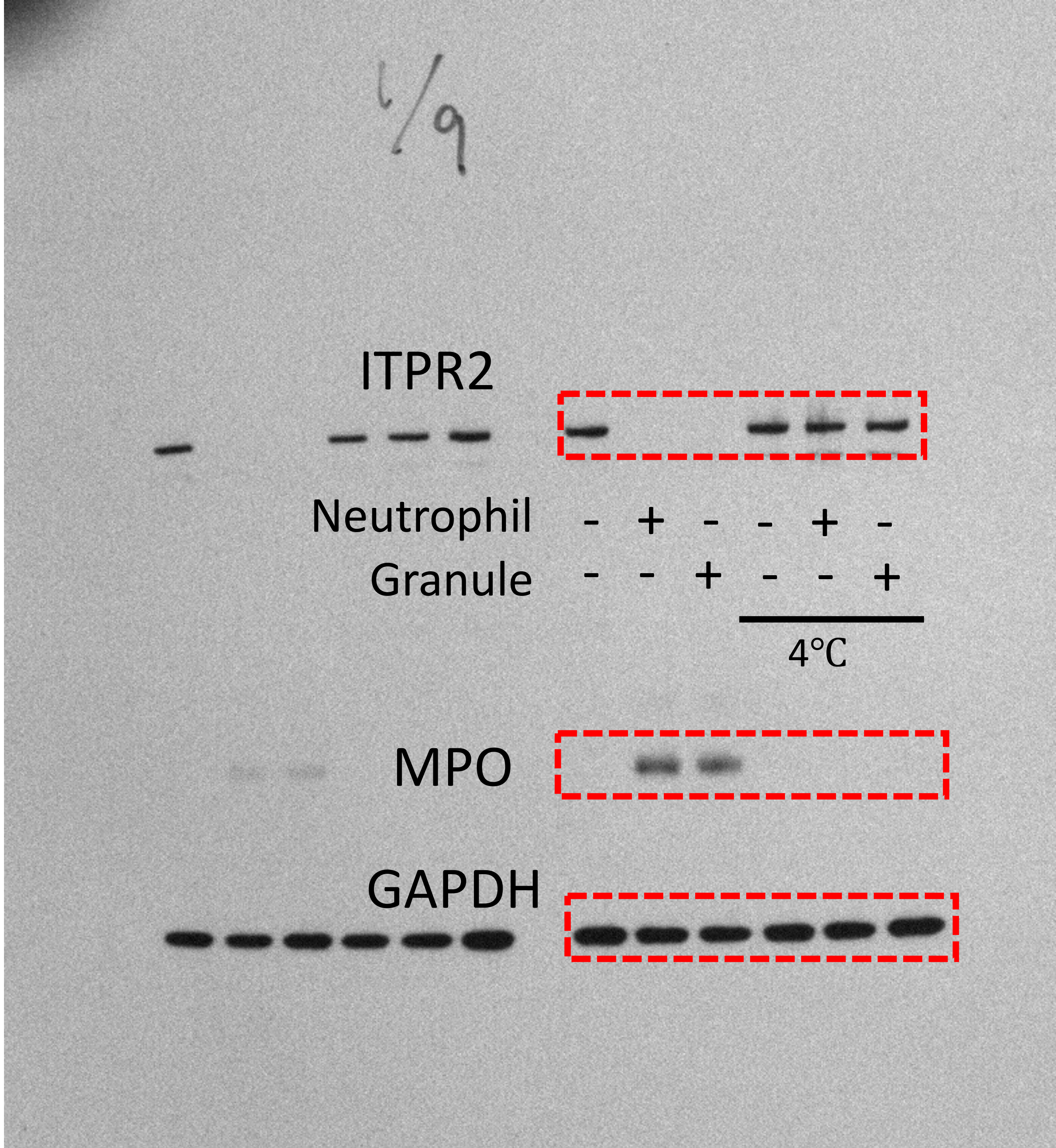

Full unedited blot for Figure 6E

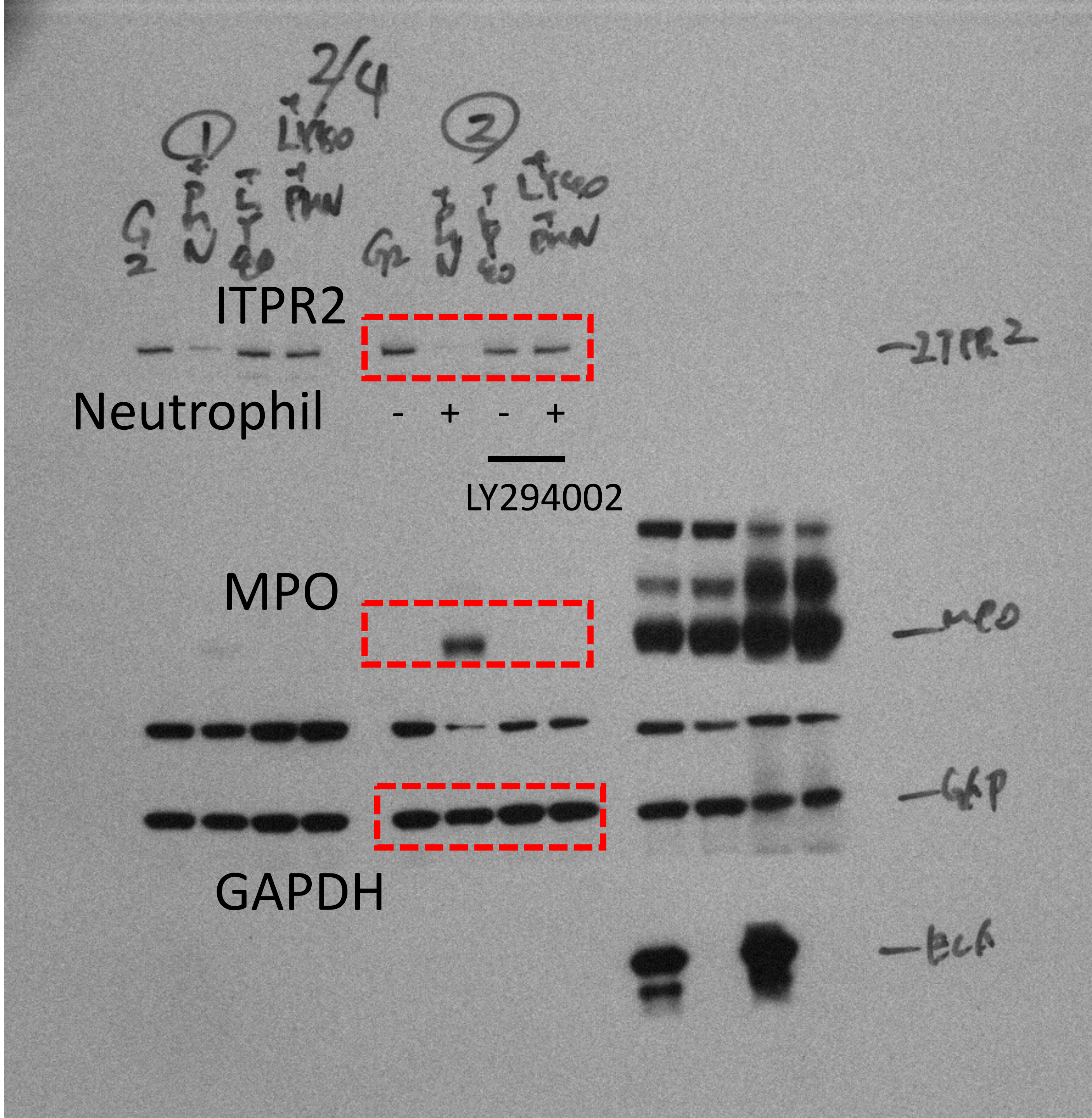

Full unedited blot
for Figure S6C

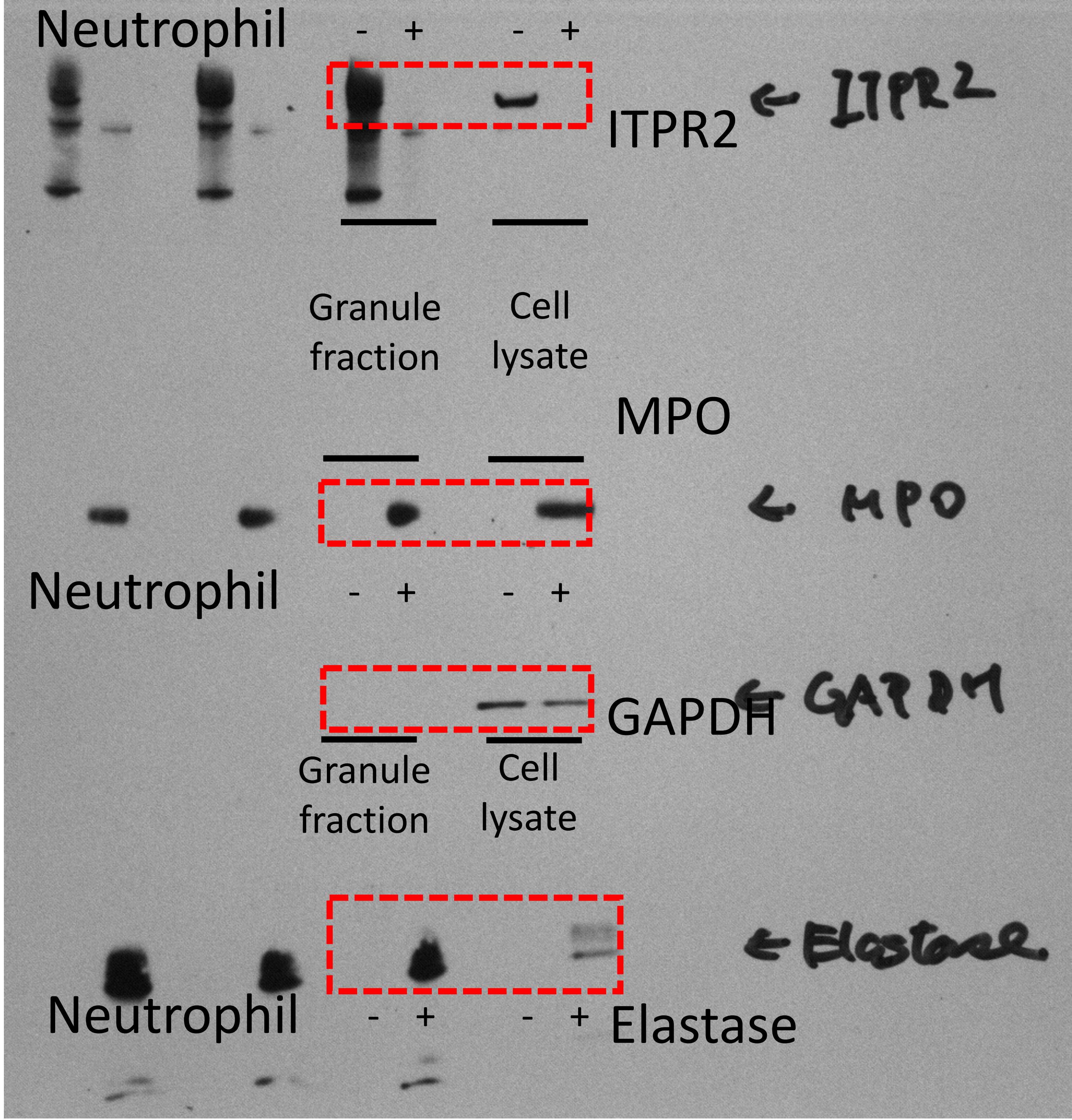

Full unedited blot for Figure S6D

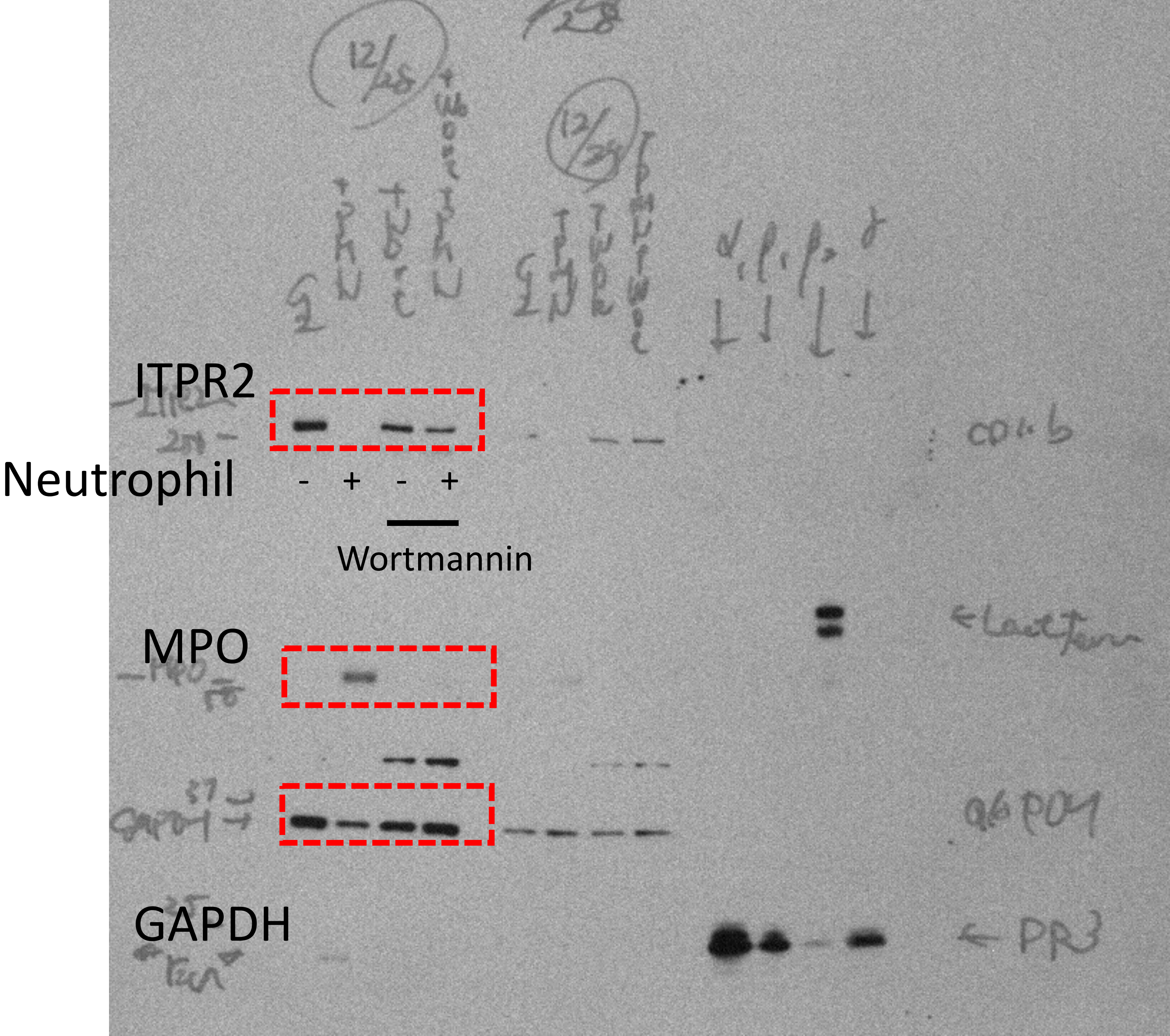

Full unedited blot for Figure S6J

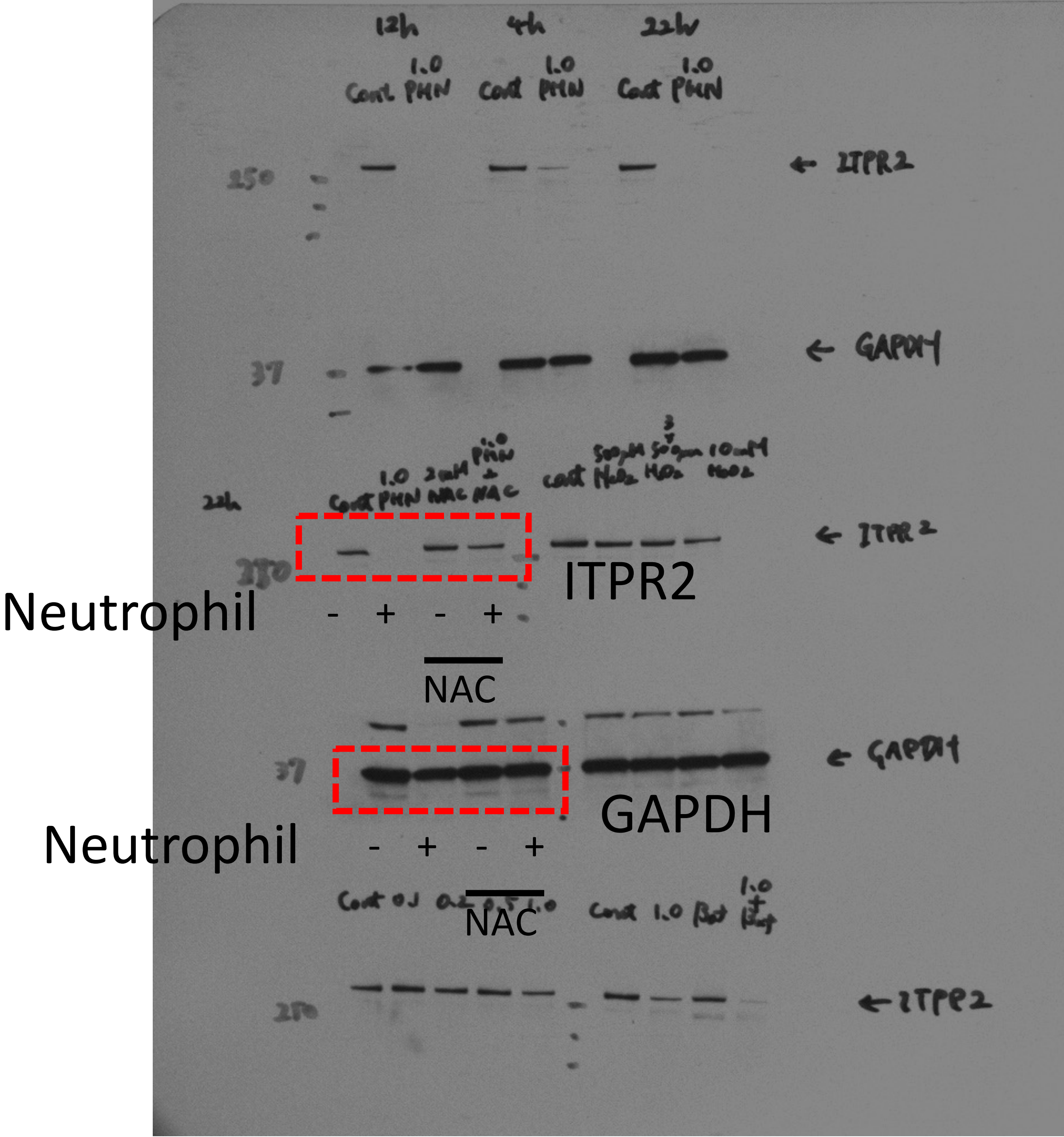

Full unedited blot for Figure 7A

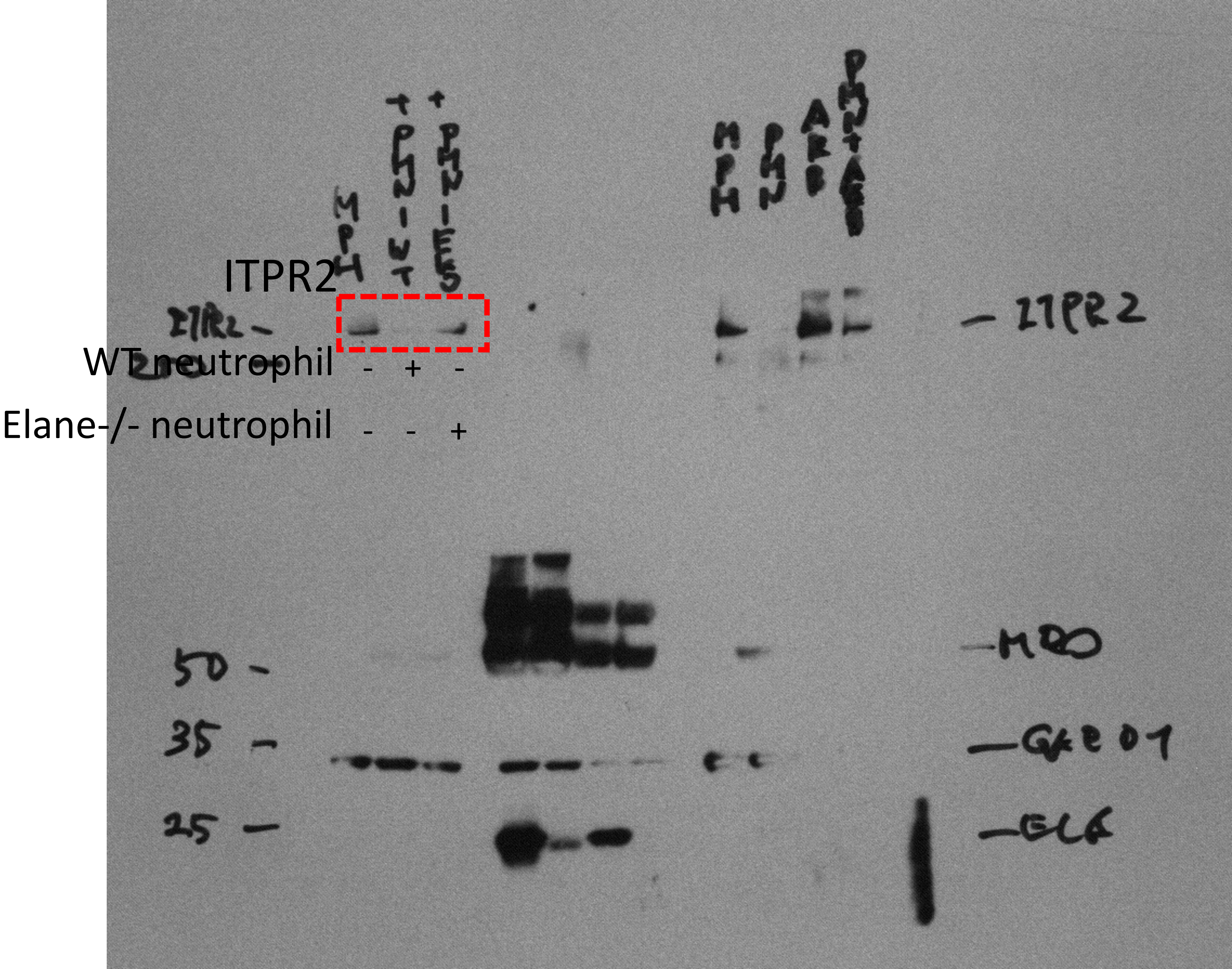

Full unedited for Figure 7A

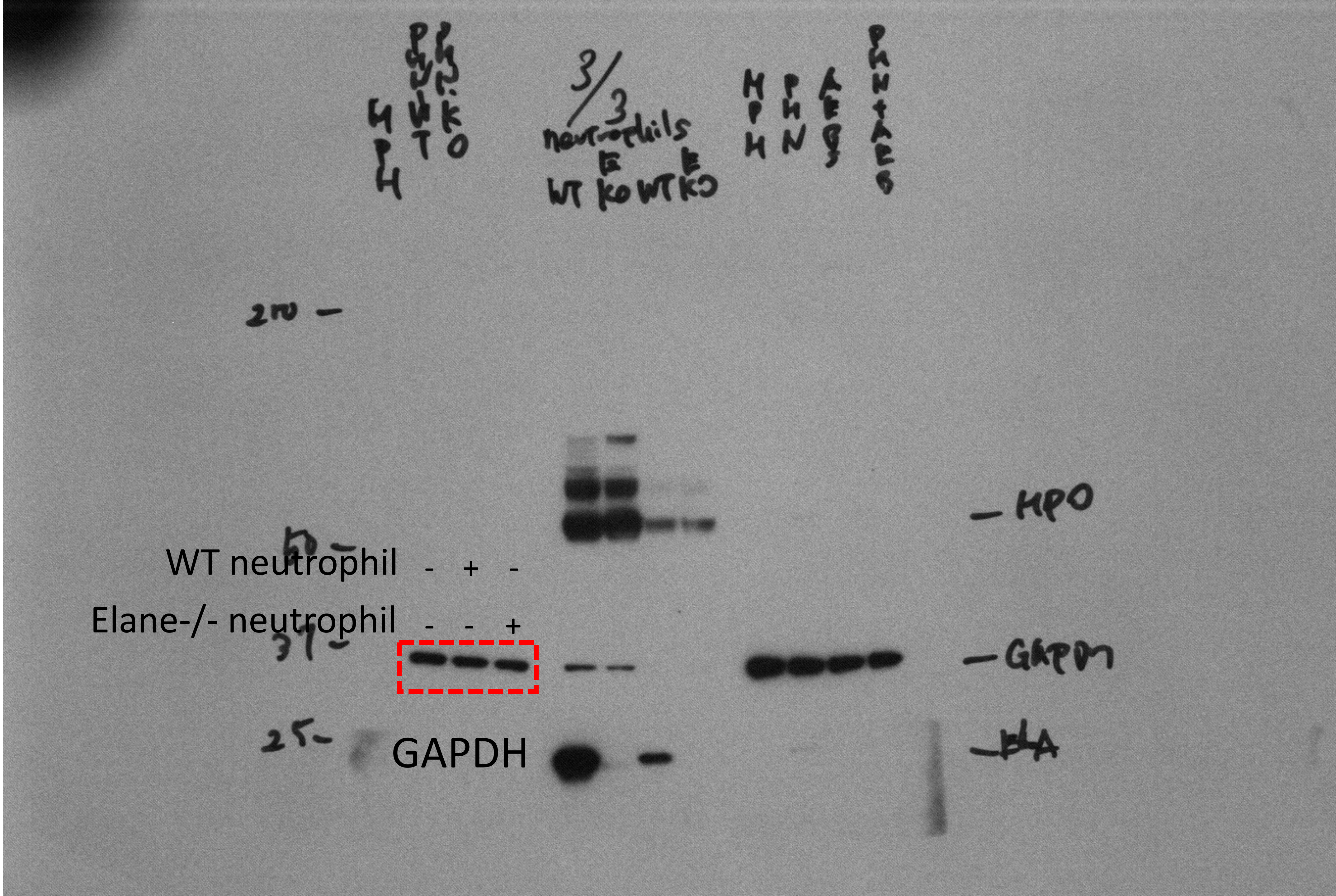

Full unedited blot for Figure 7C

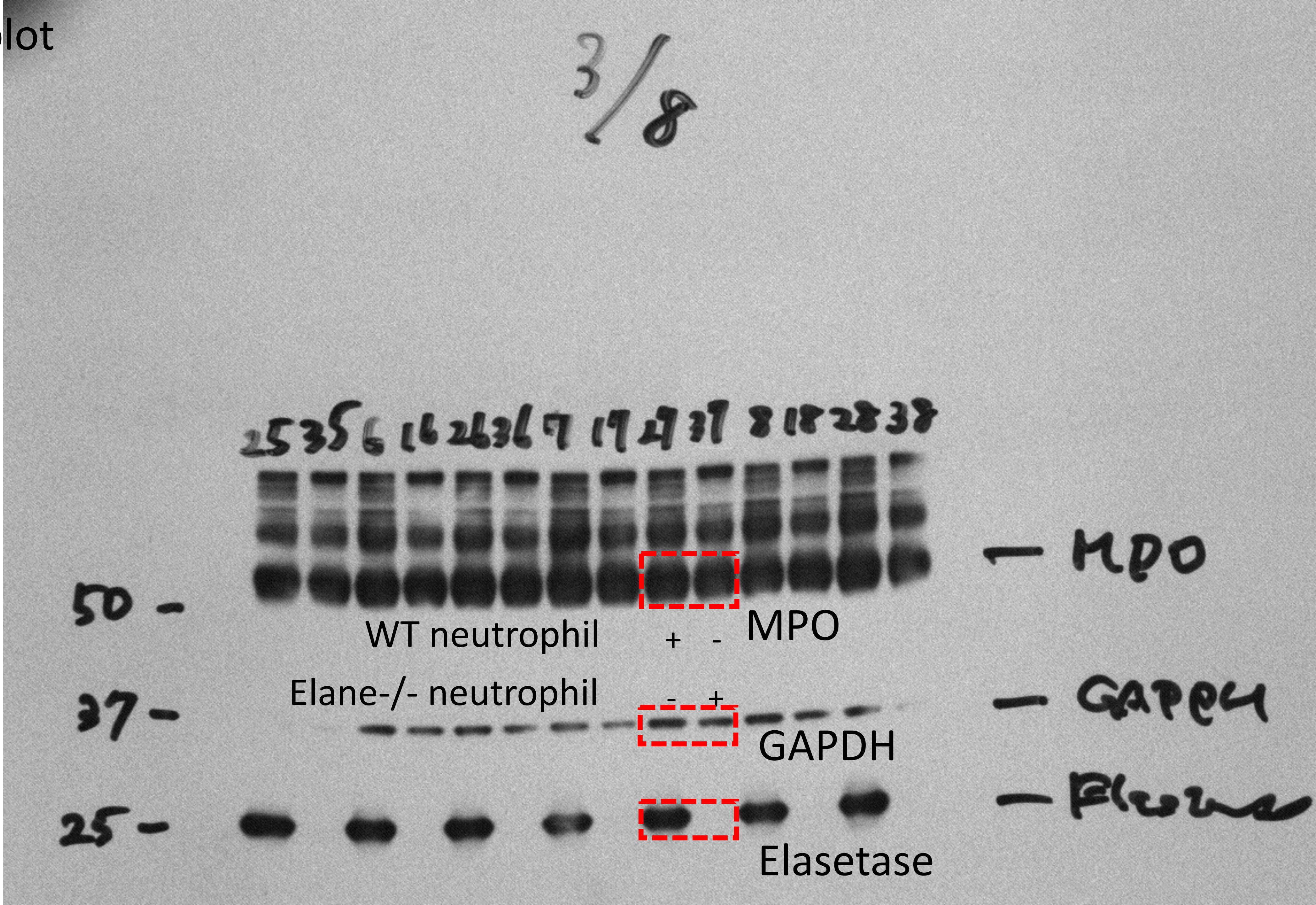

Full unedited blot
for Figure 7D

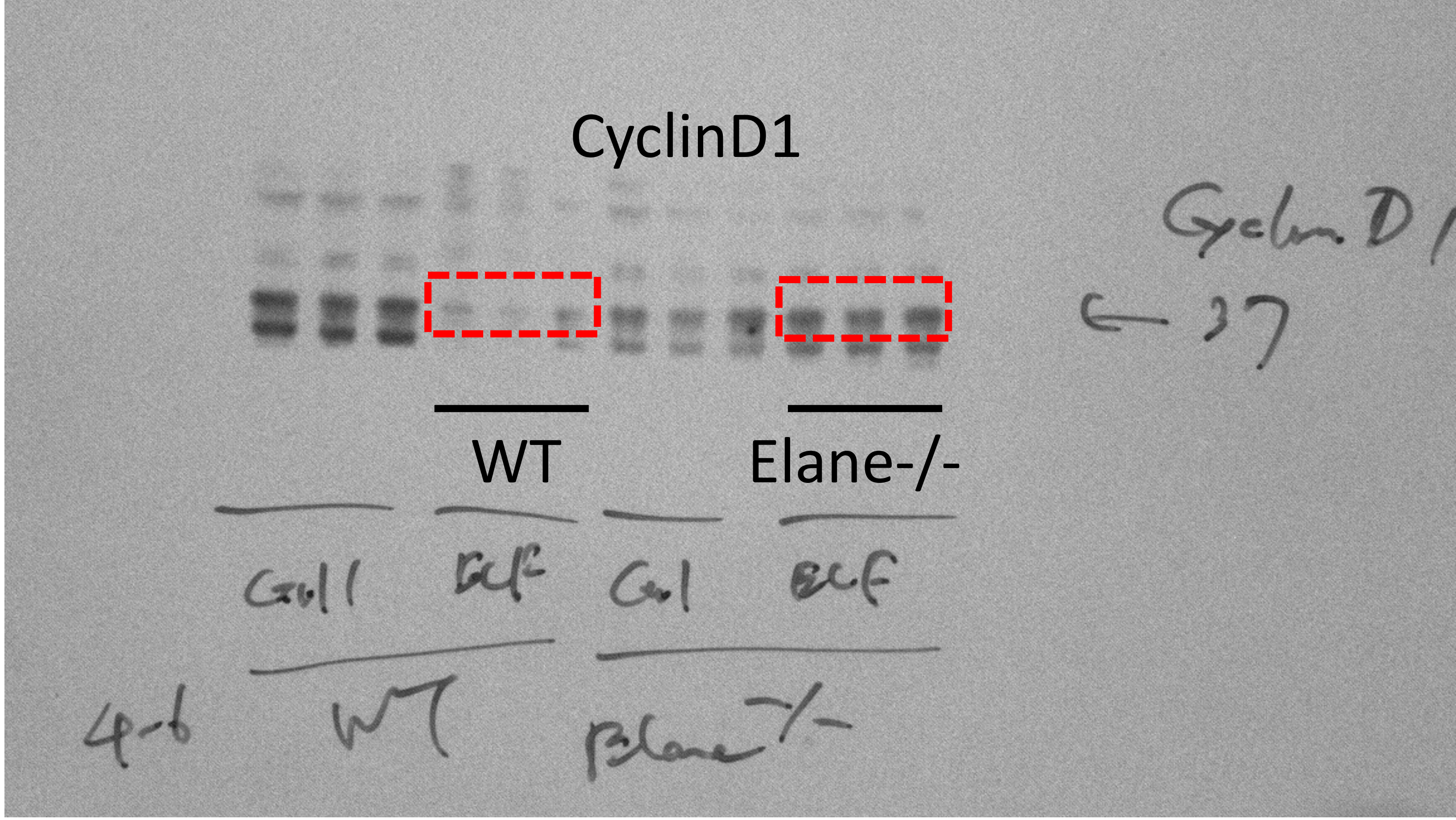

Full unedited blot for Figure 7D

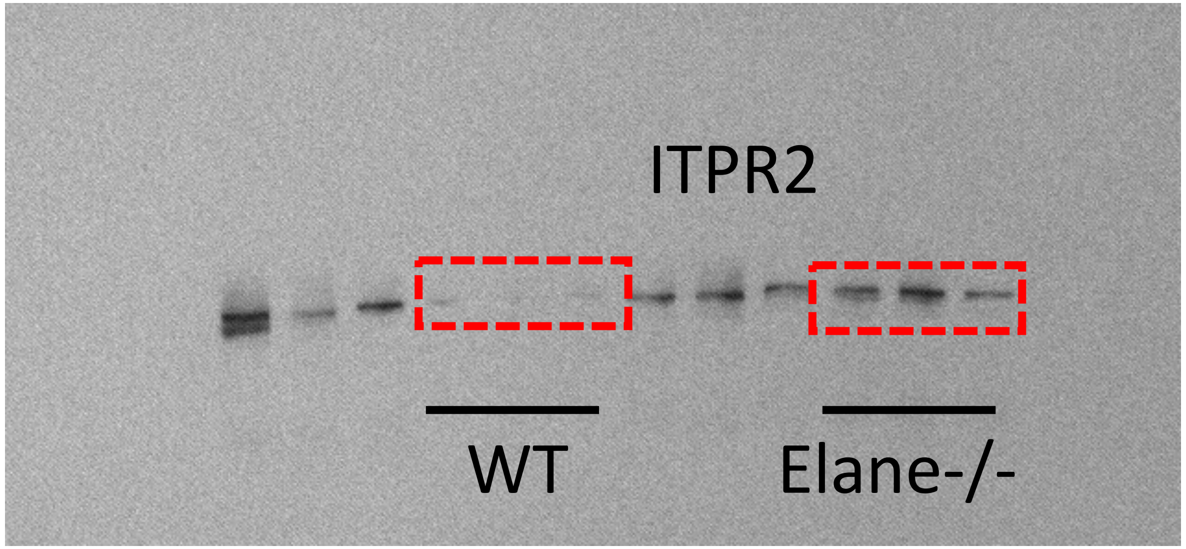

Full unedited blot for Figure 7D

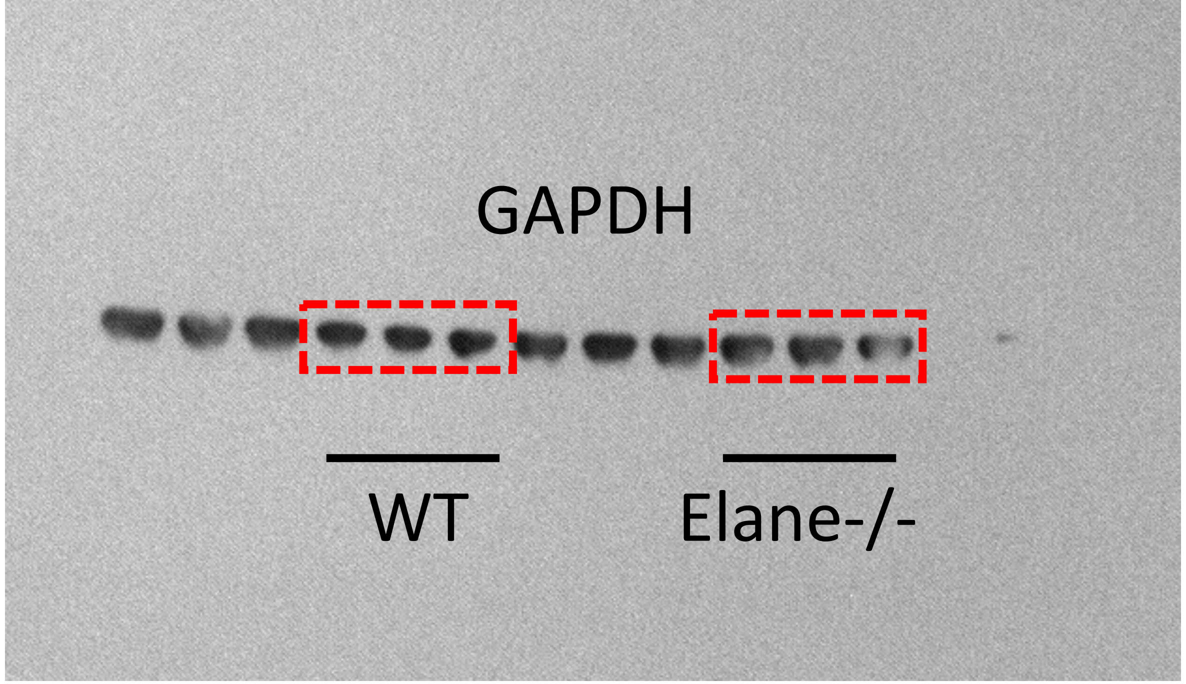

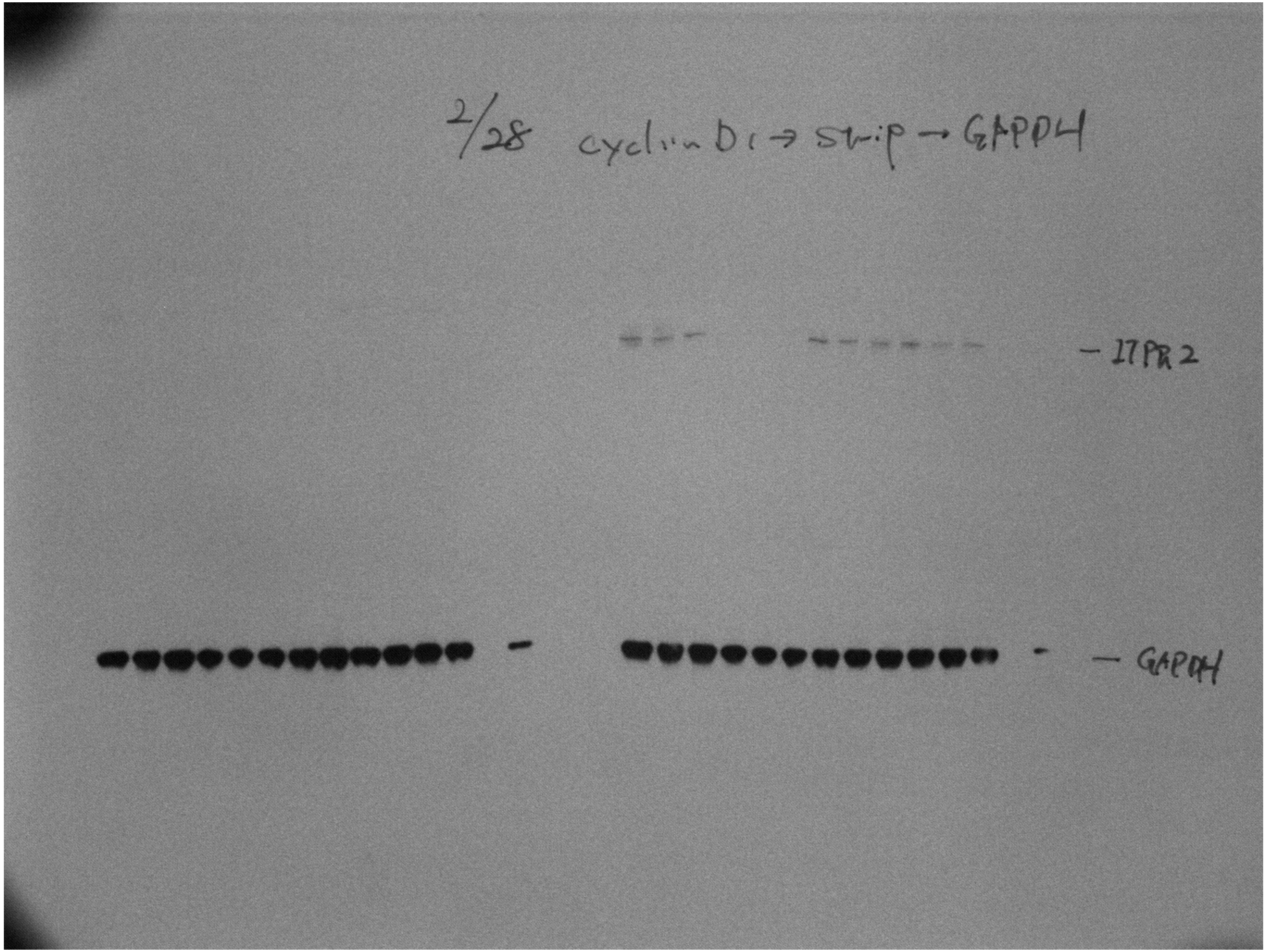
2/28 cyclin D1 → strip → GAPDH
— ITPR2
— GAPDH

Full unedited blot for Figure 7I

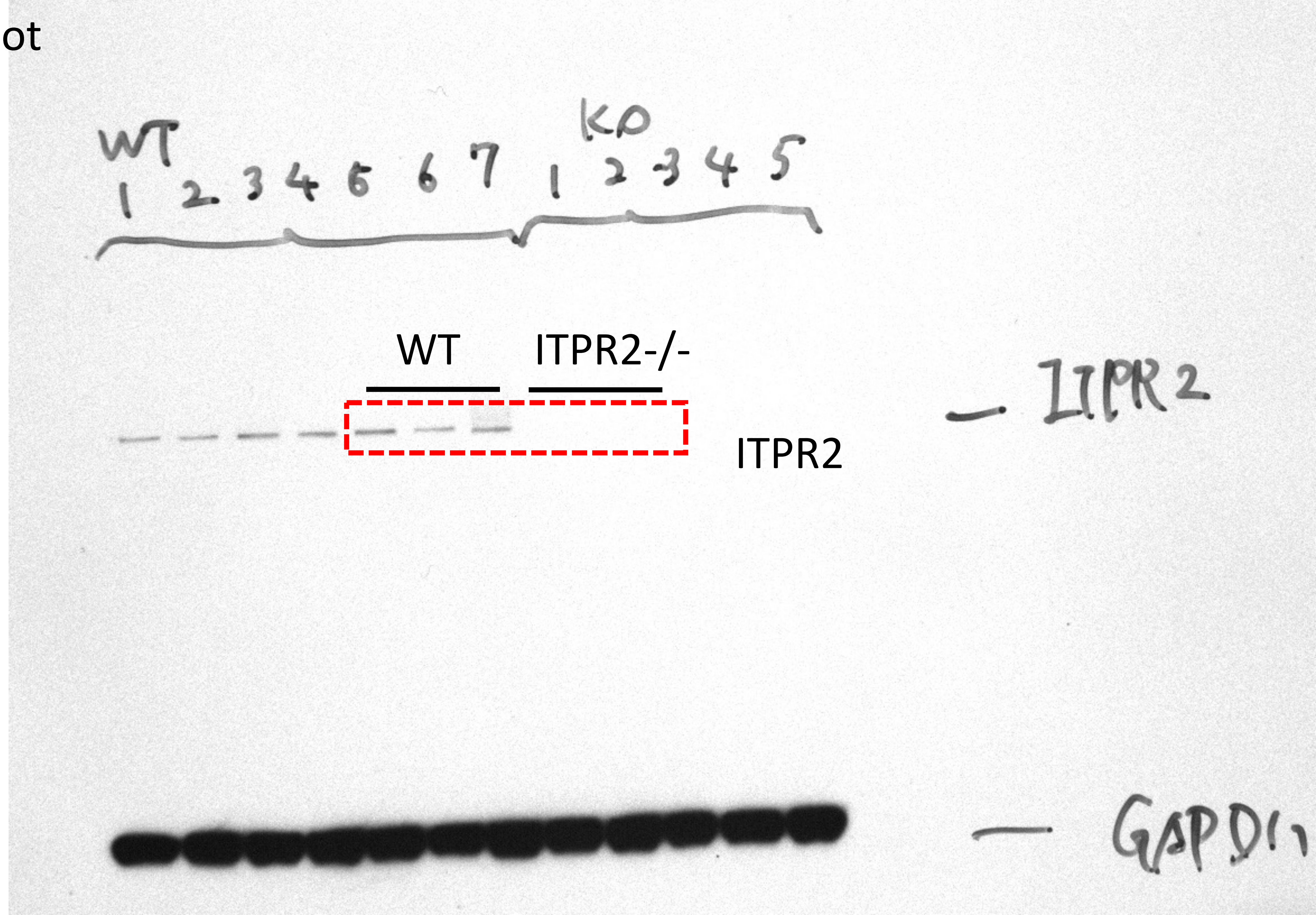

Full unedited blot for Figure 7I

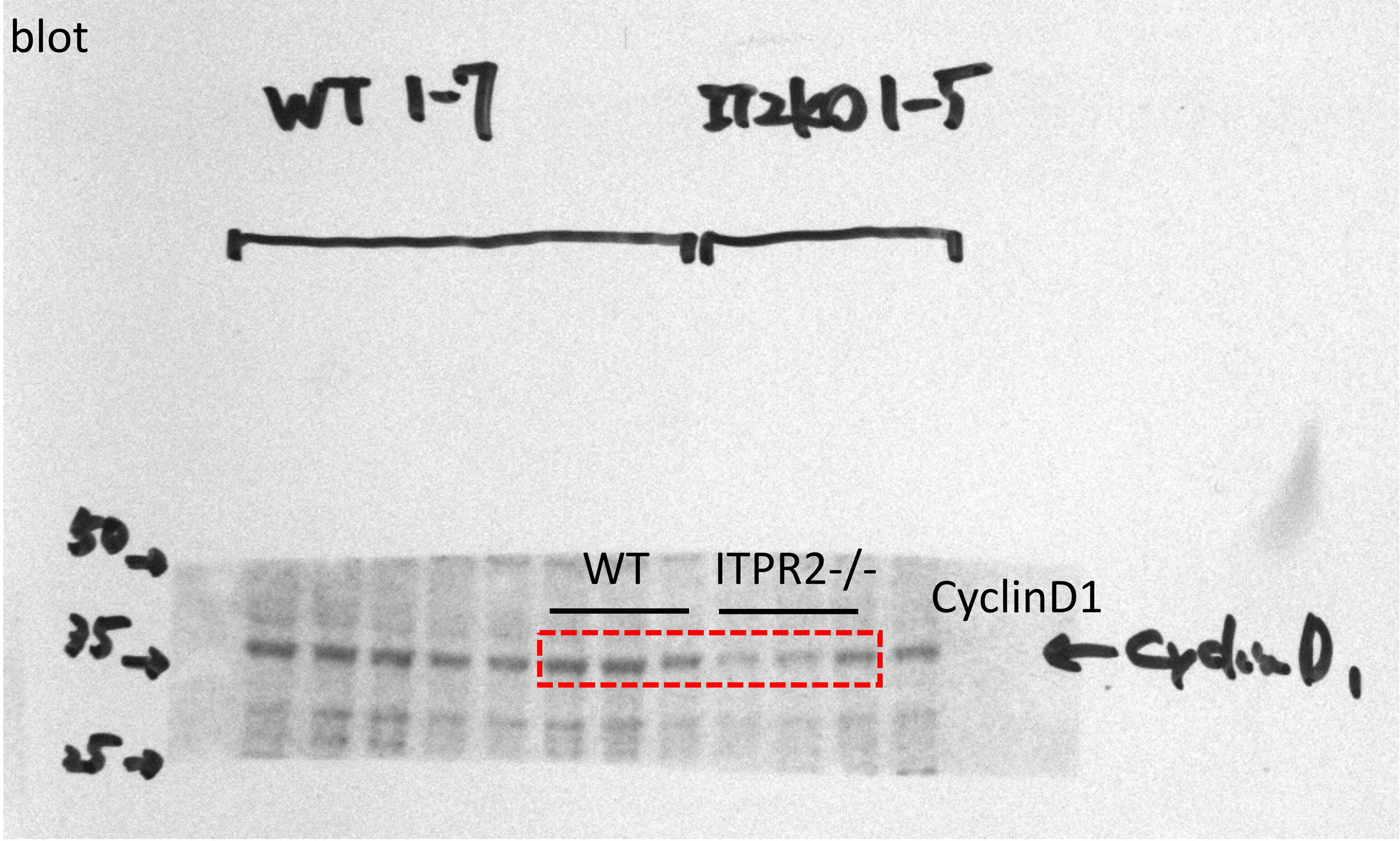

Full unedited blot for Figure 7I

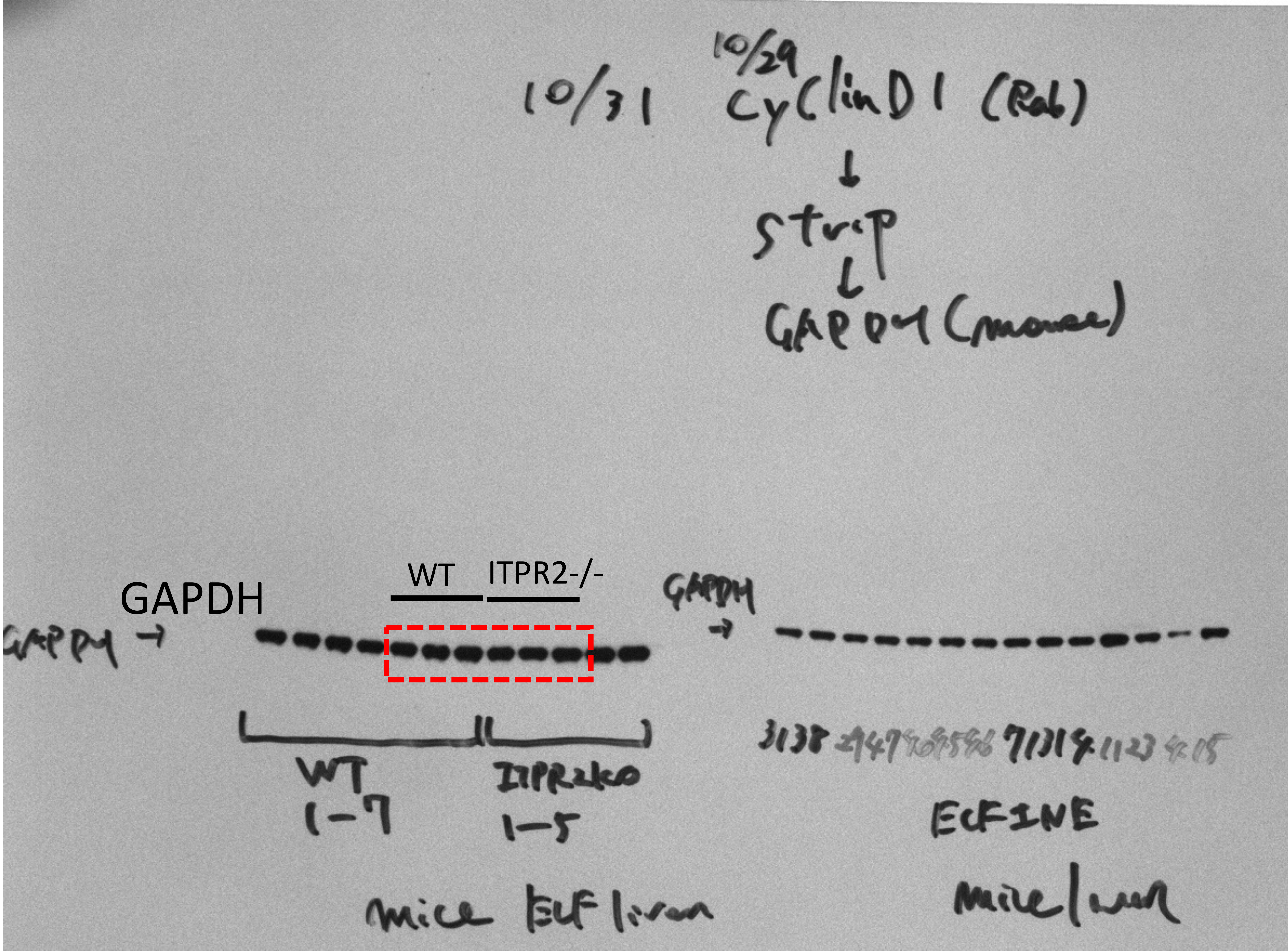

Full unedited blot for Figure S7A

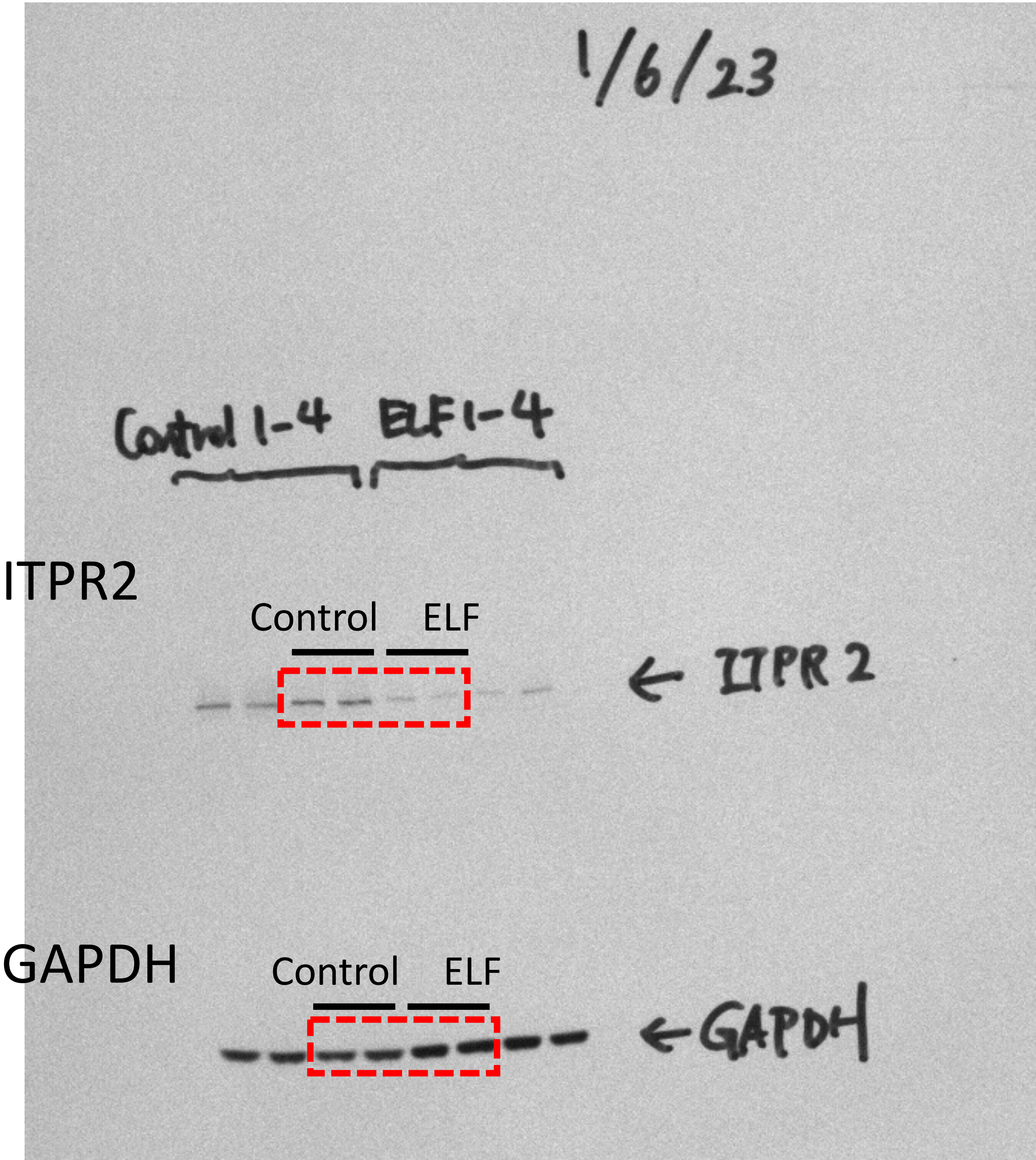

Full unedited blot for Figure S7G

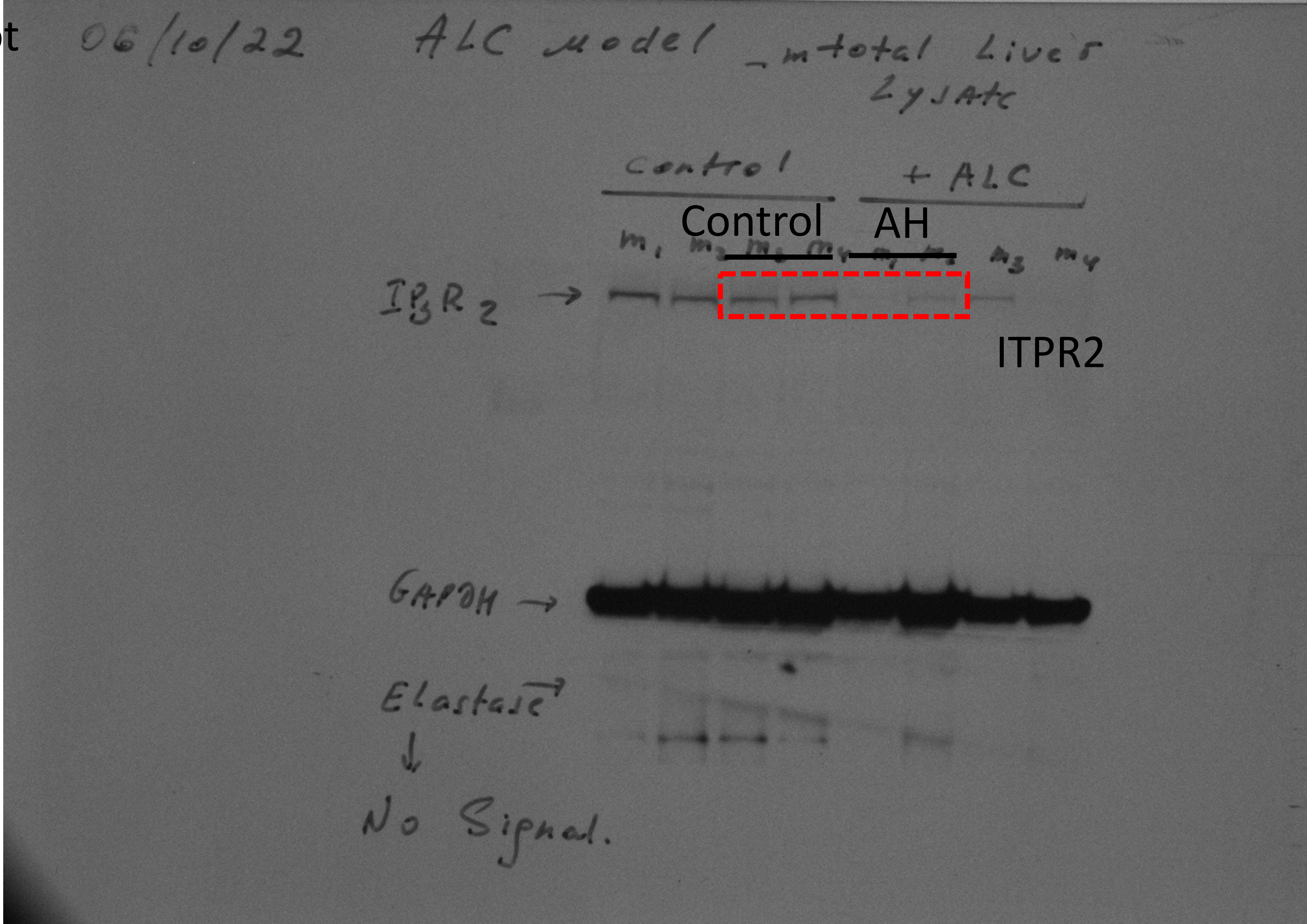

Full unedited blot for Figure S7G

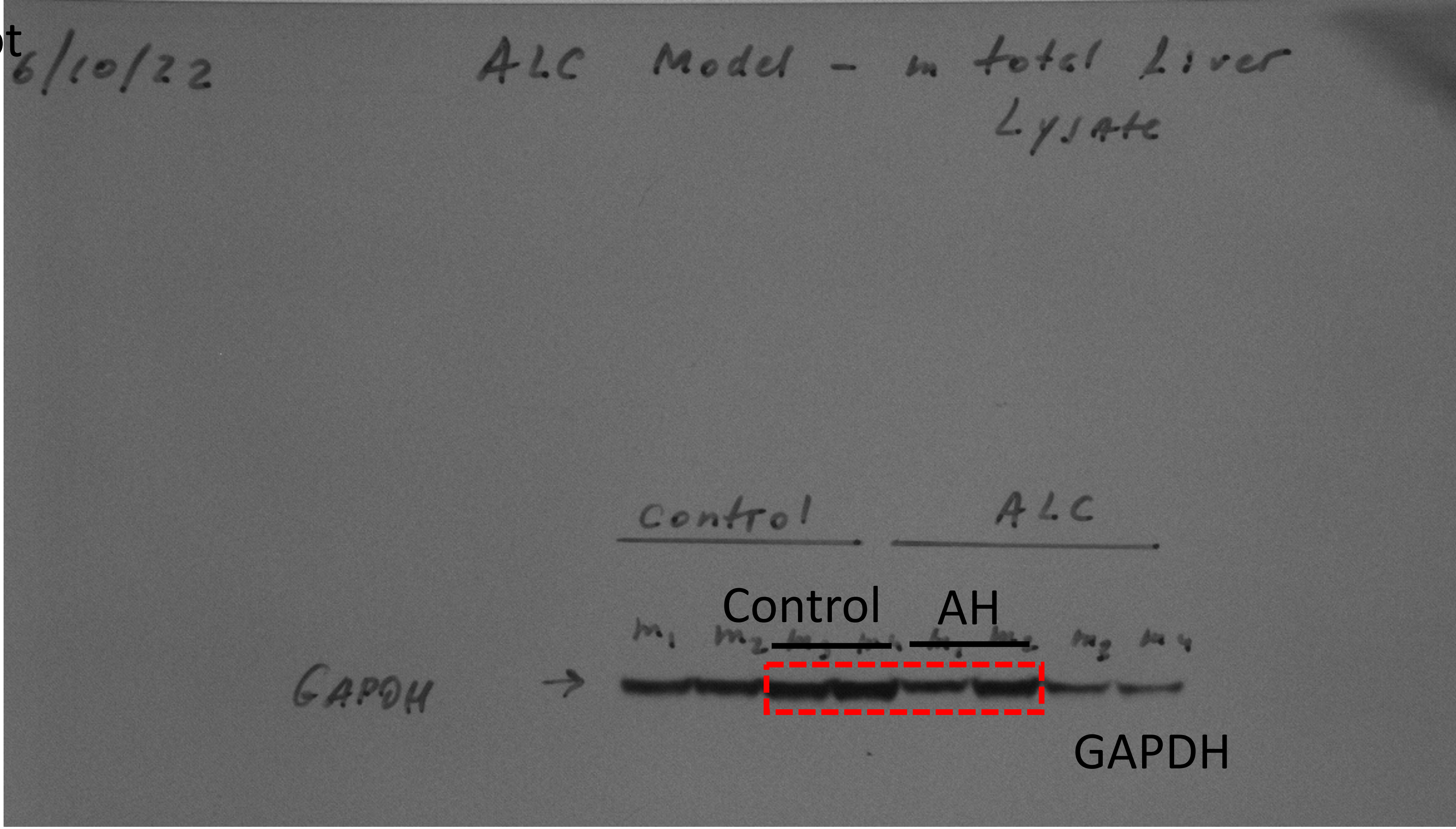

Full unedited blot
for Figure S7N

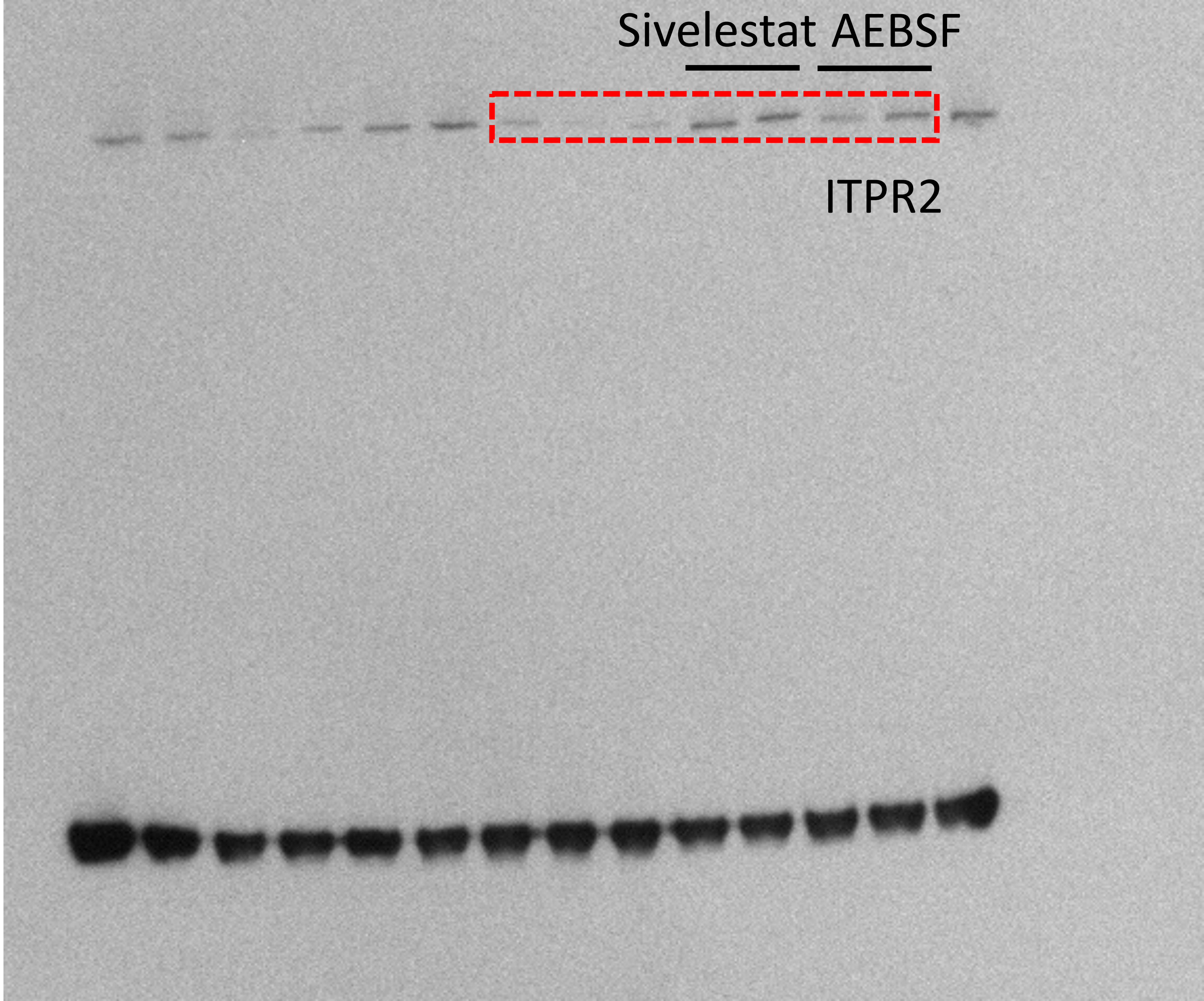

Full unedited blot for Figure S7N

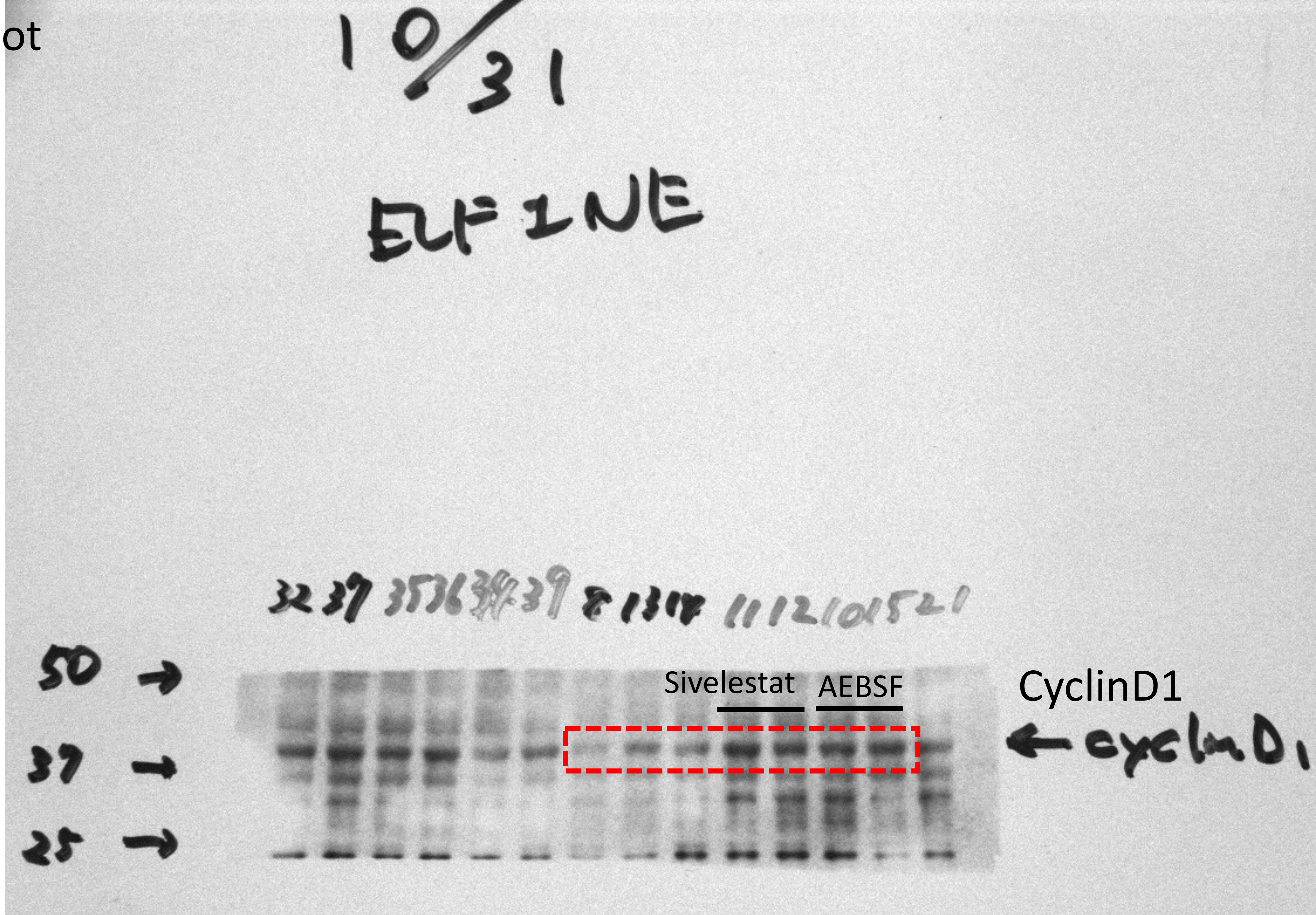

Full unedited blot for Figure S7N

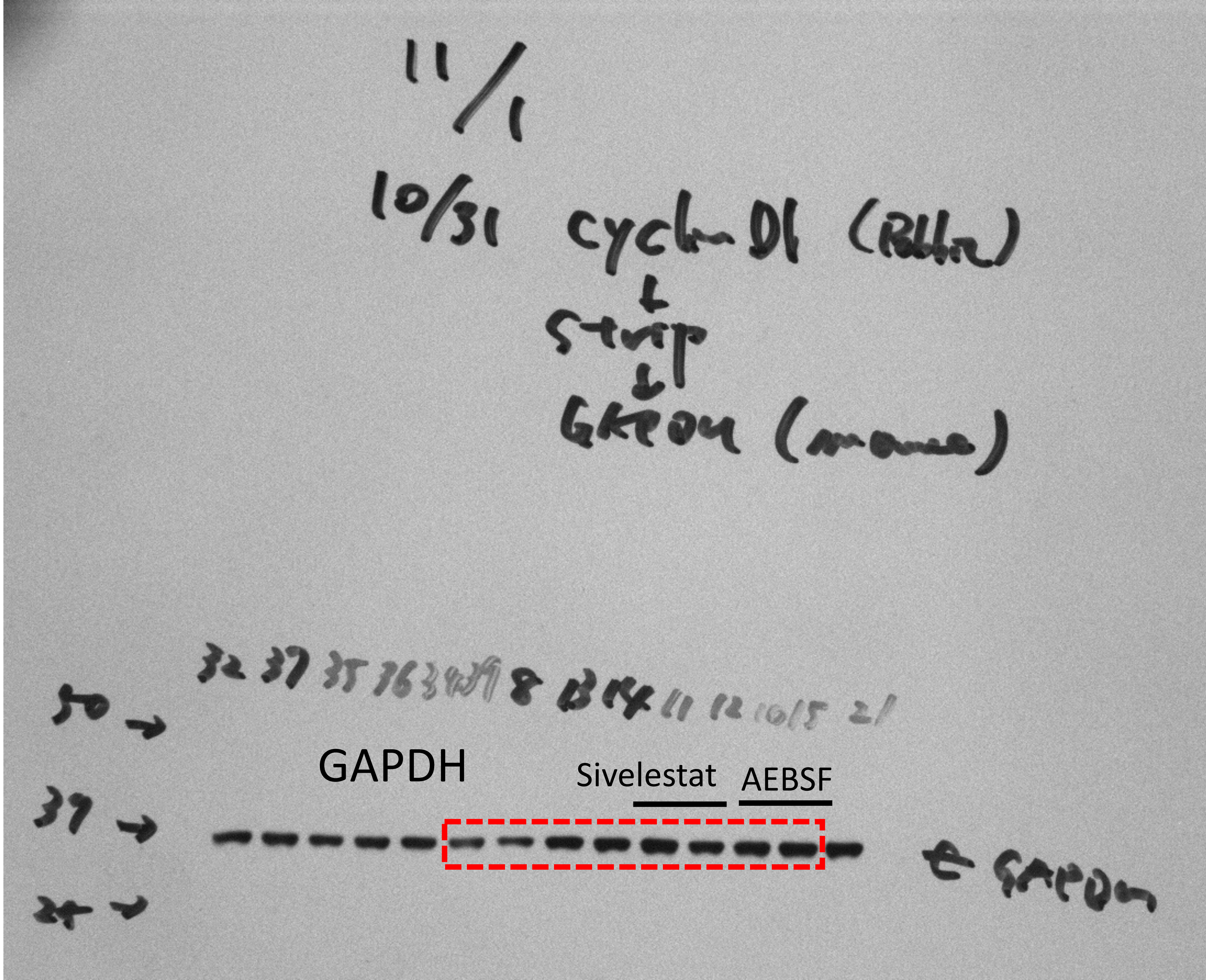

Full unedited blot
for Figure 8

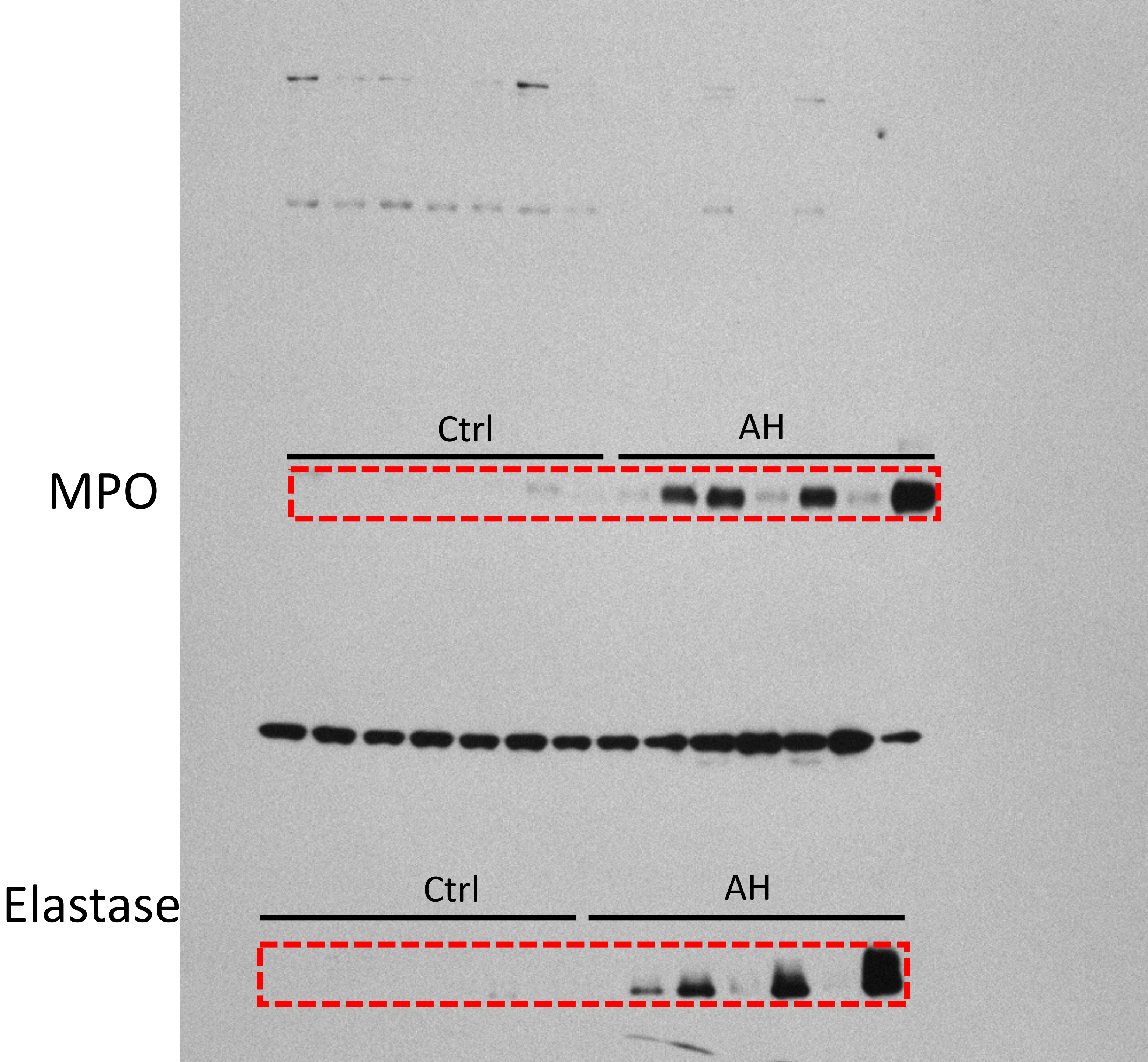

Full unedited blot for Figure 8

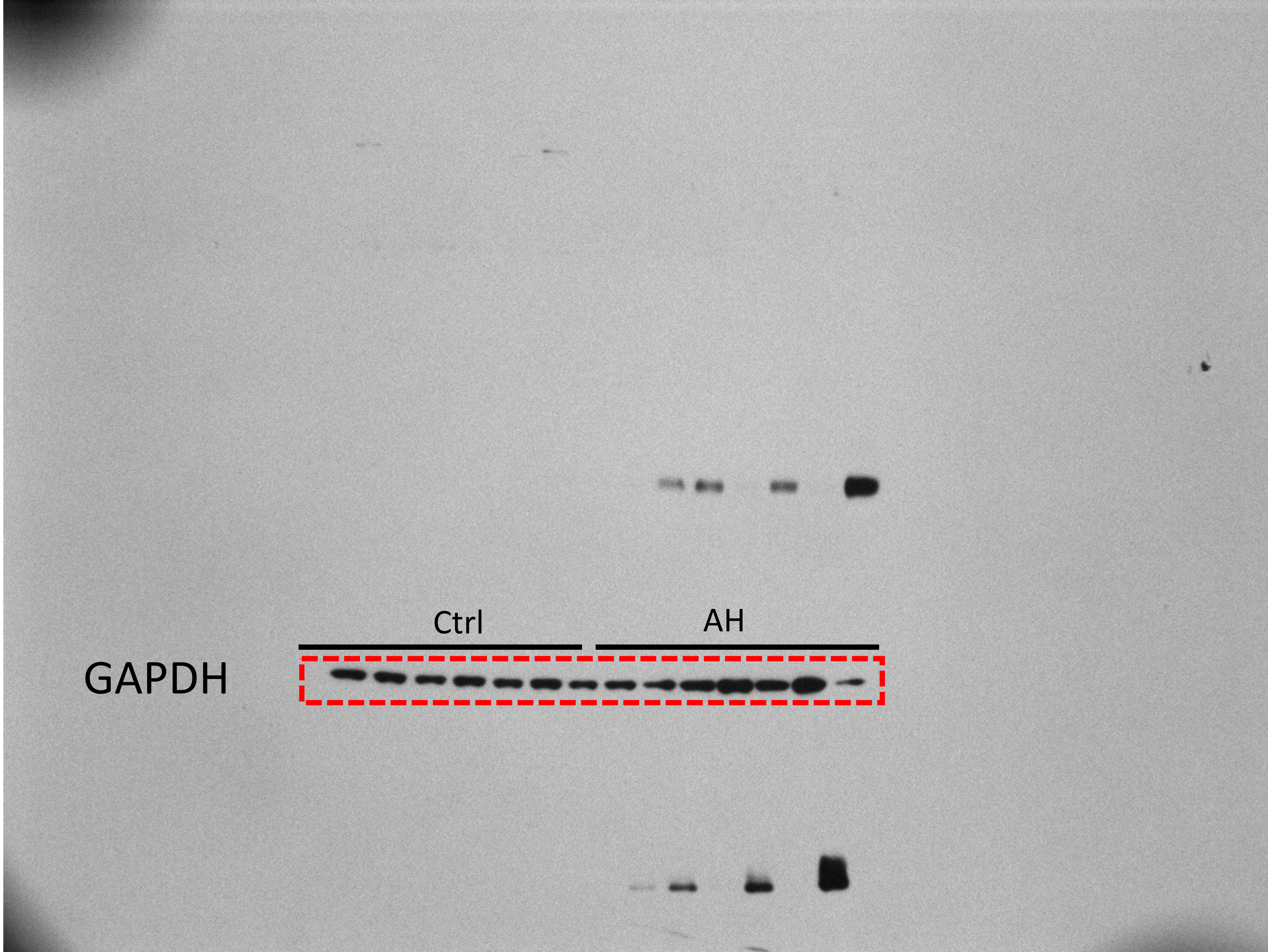

Full unedited blot
for Figure 8

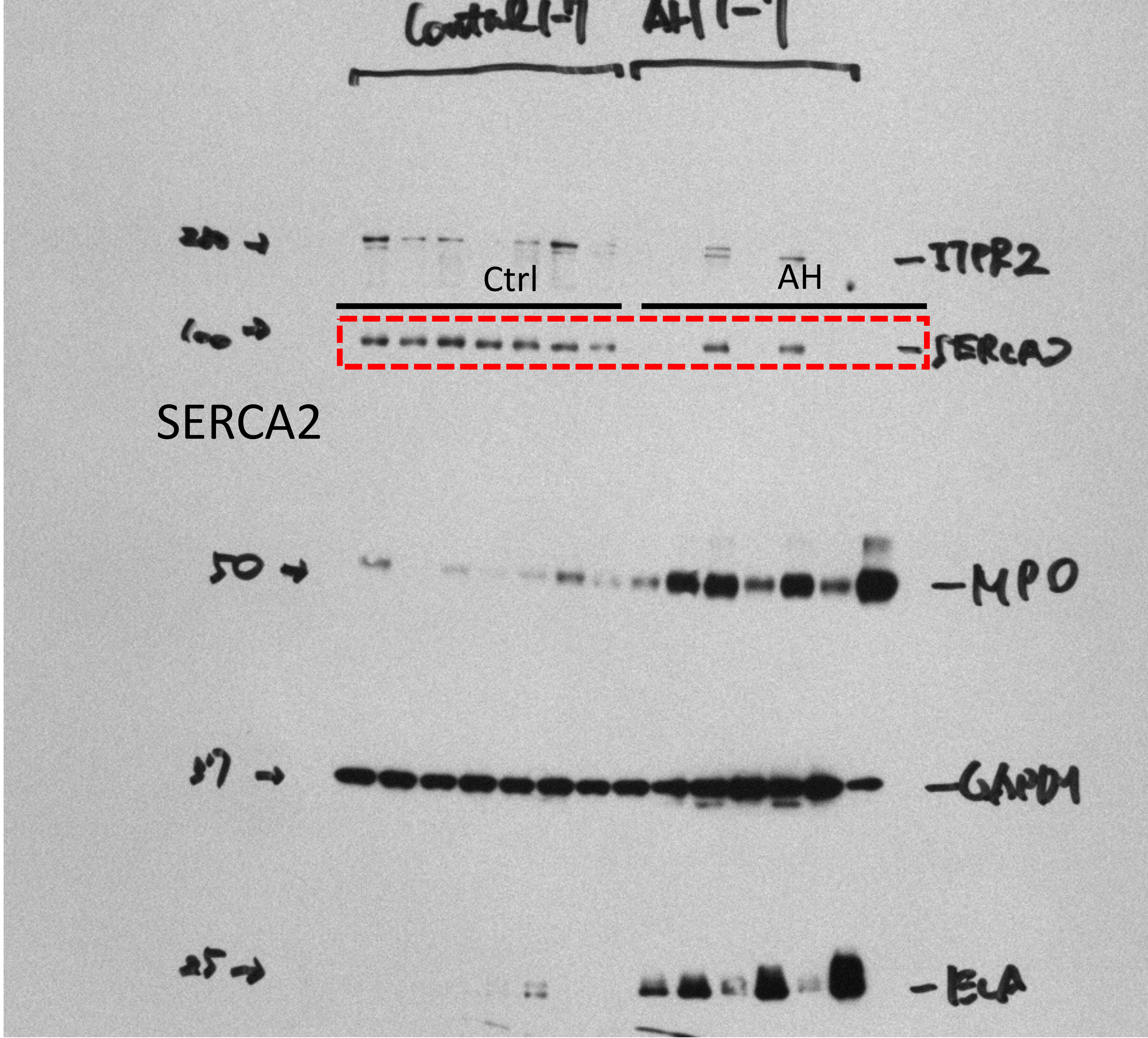

Full unedited blot
for Figure 8

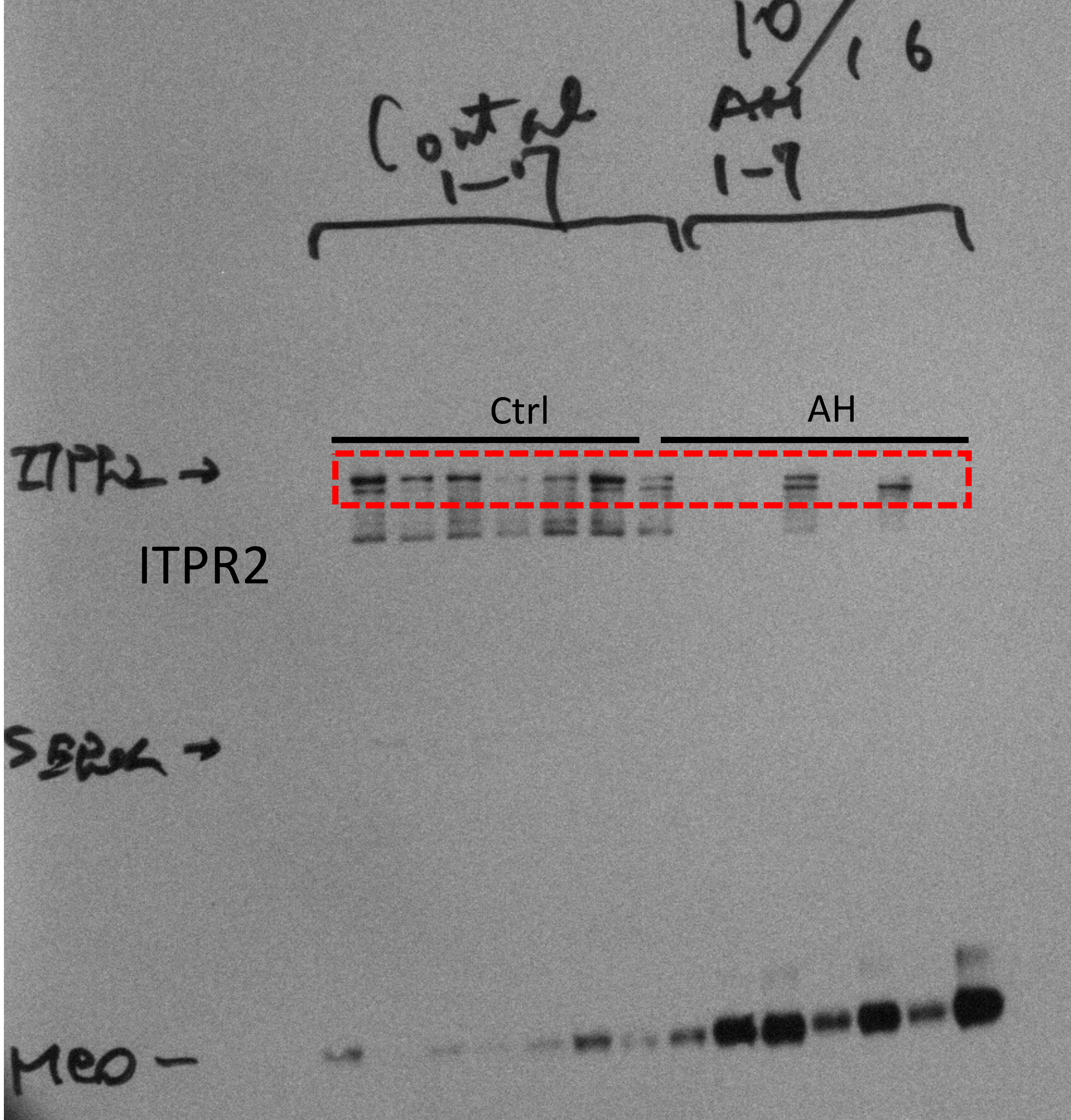

Full unedited blot
for Figure S8I

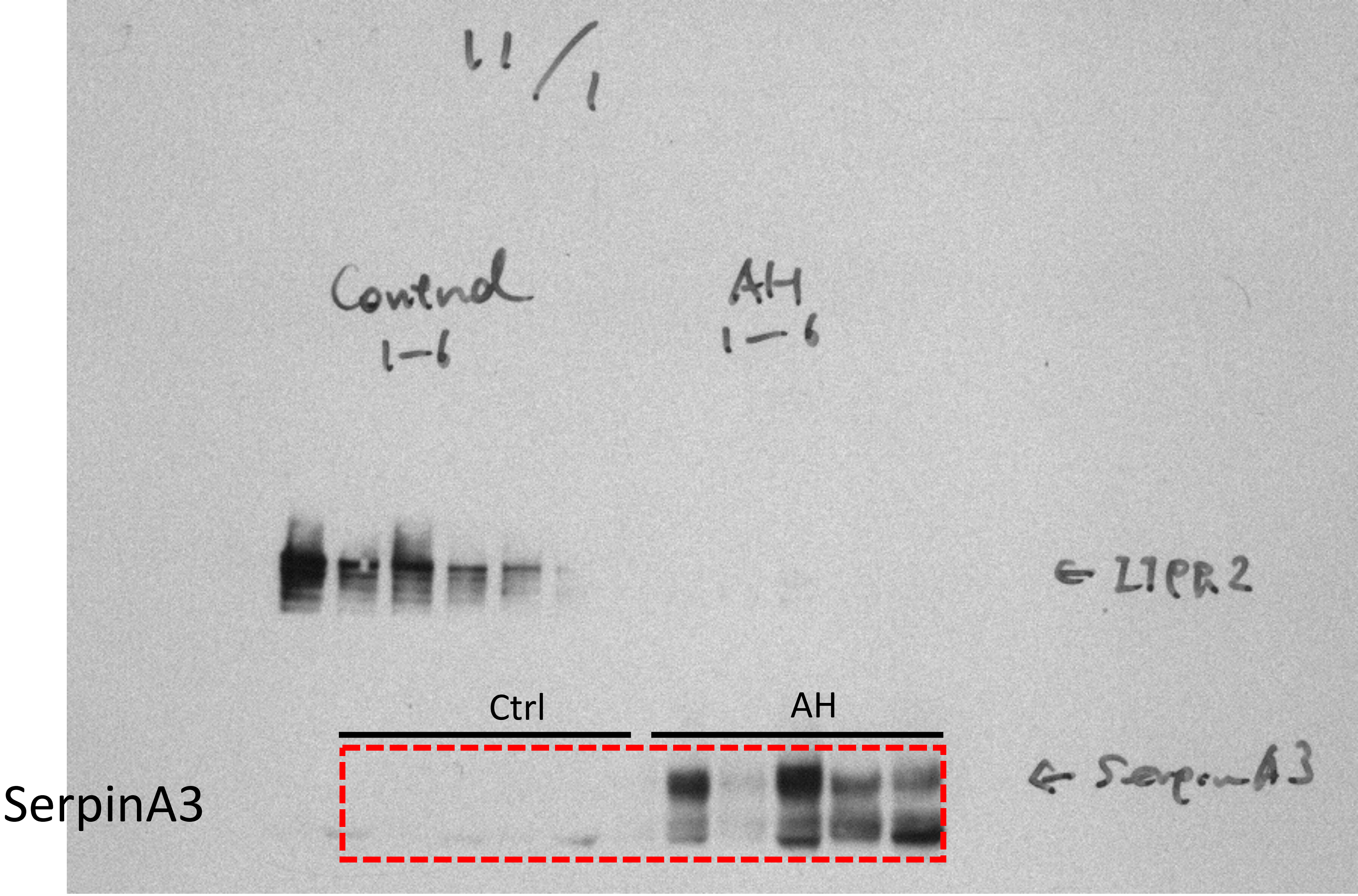

Full unedited blot for Figure S8I

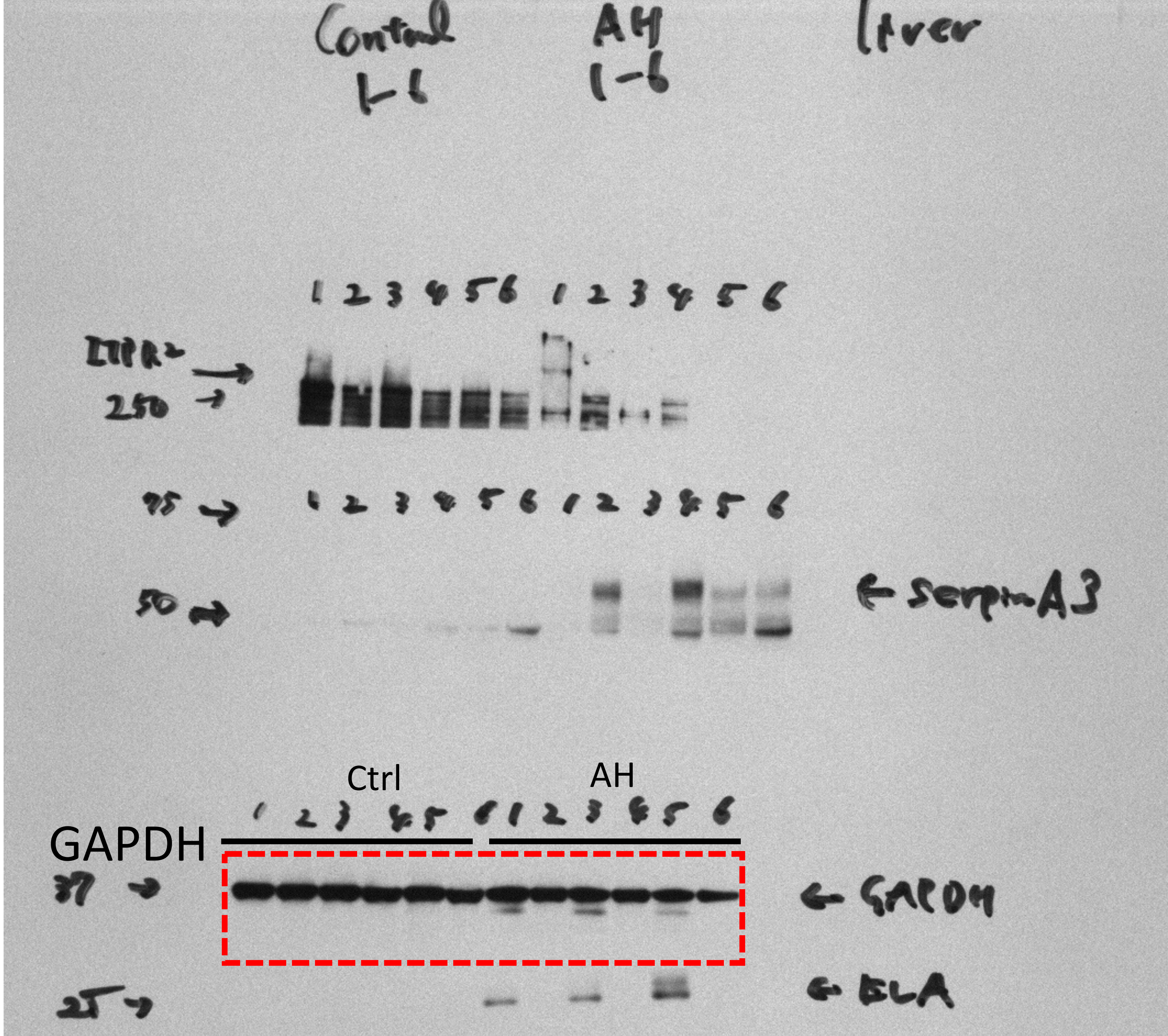

Full unedited blot for Figure 9A

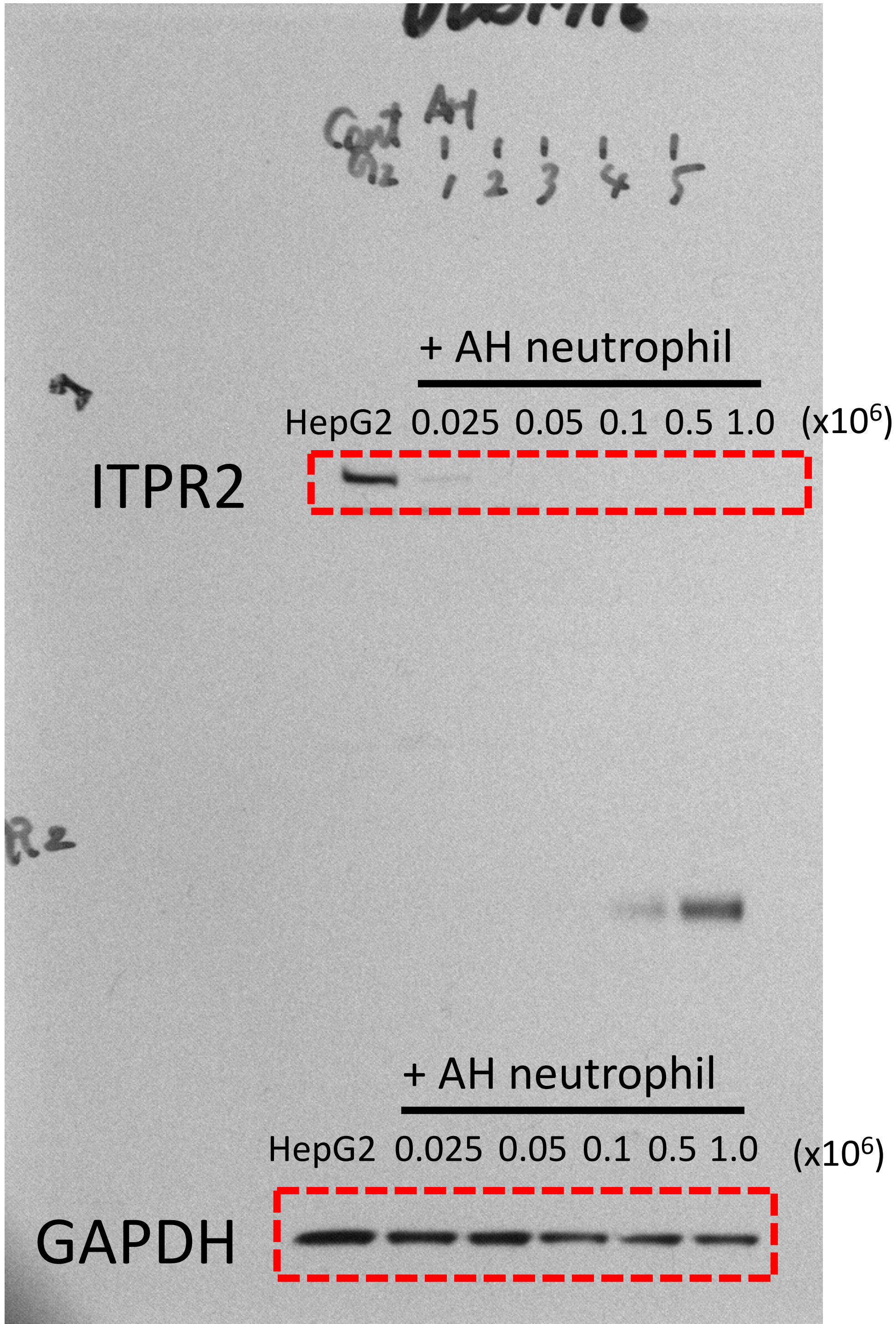

Full unedited blot
for Figure 9B

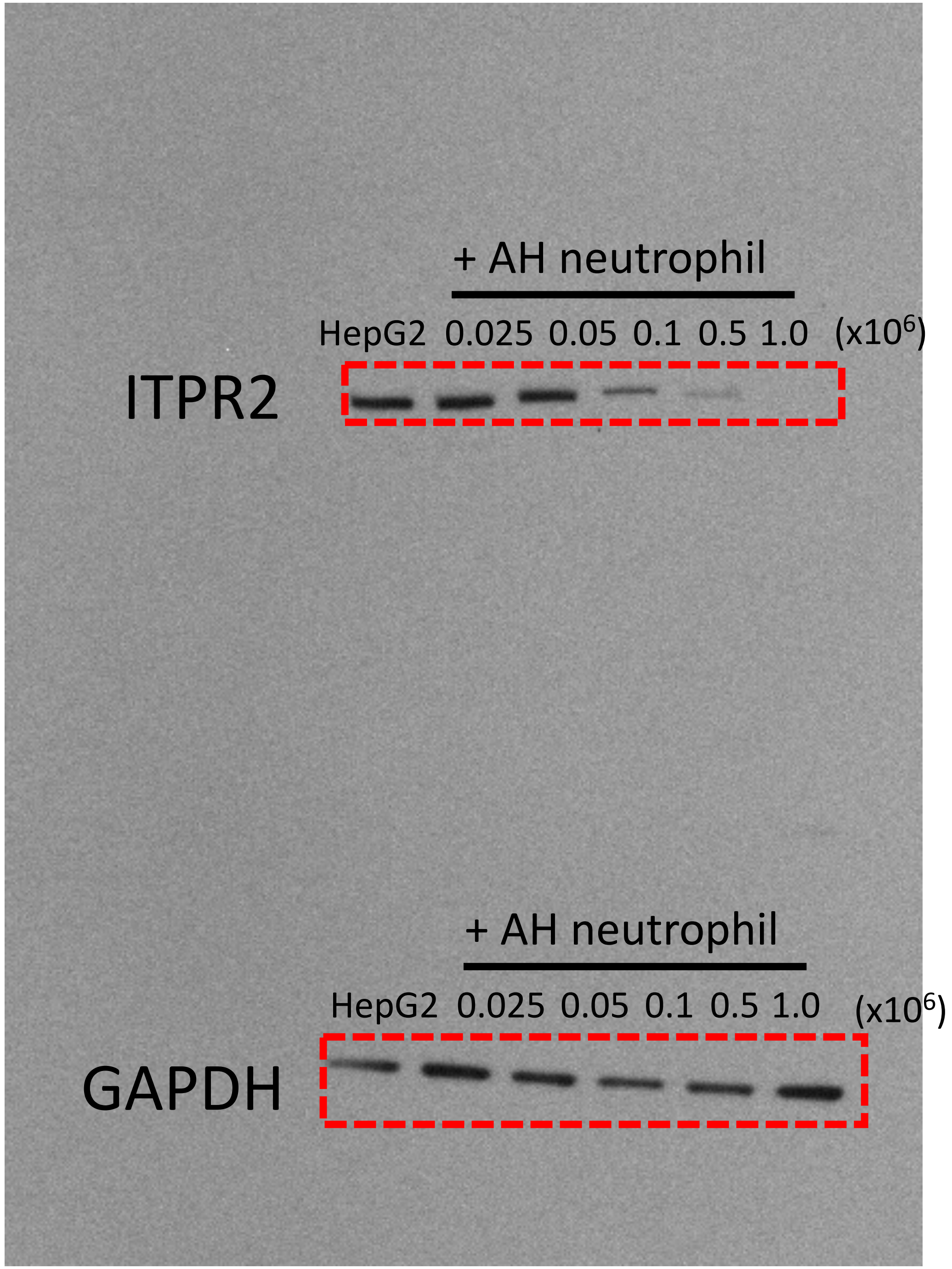

Full unedited blot for Figure 9H

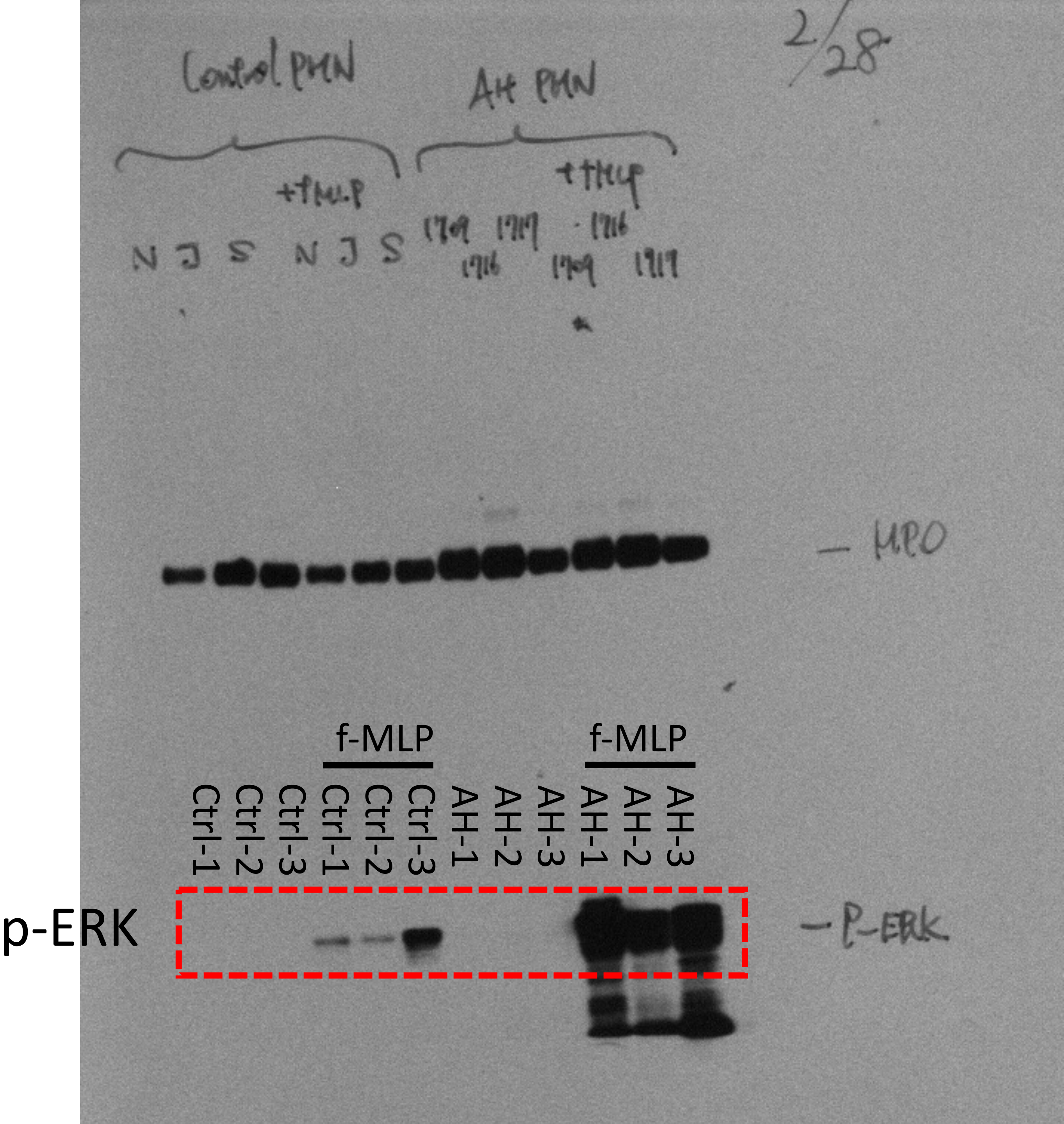

Full unedited blot for Figure 9H

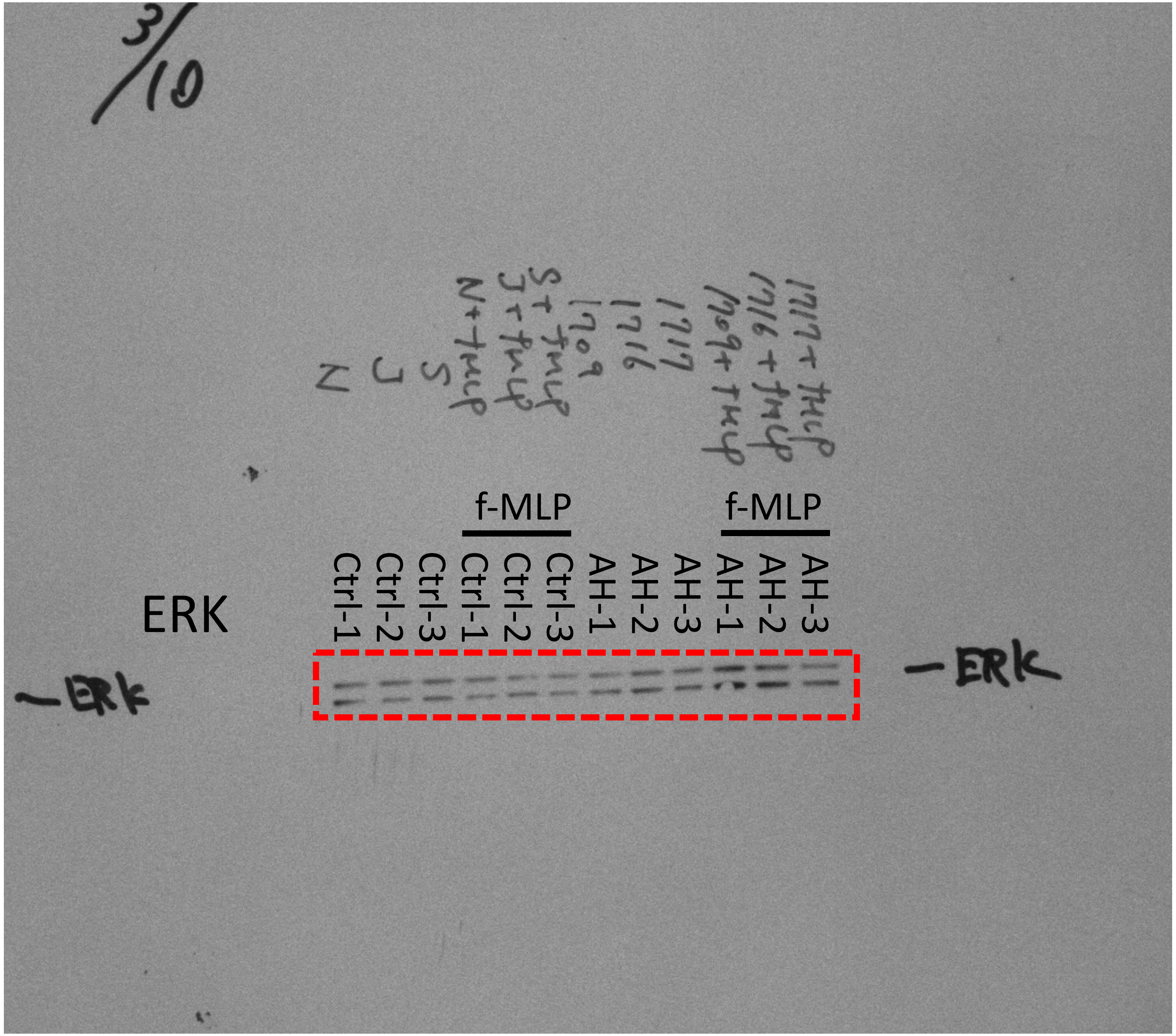

Full unedited blot for Figure 9E

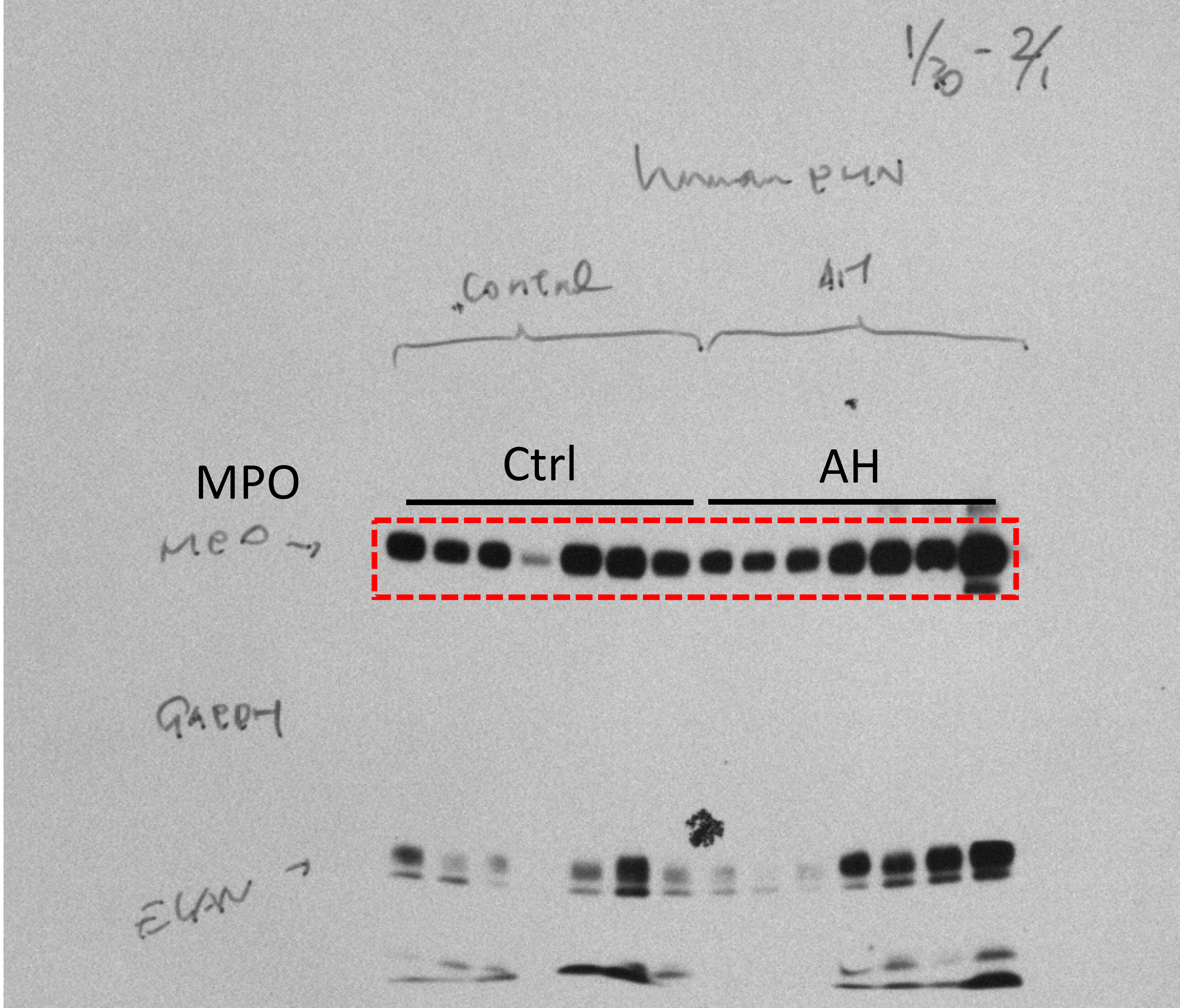

Full unedited blot
for Figure 9E

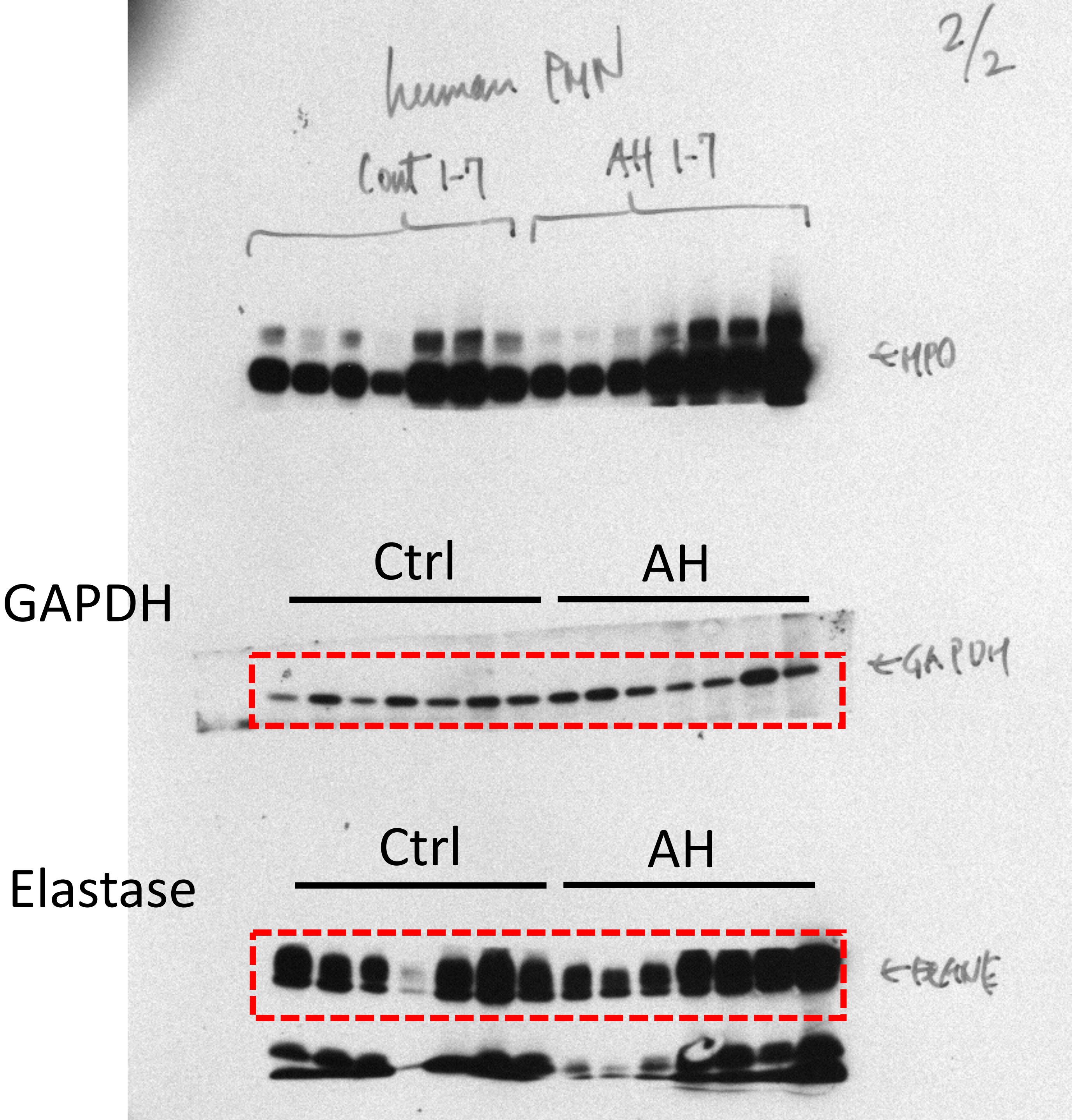

Supplement: Unedited blot and gel images [file jci-134-171691-s043.pdf]
